# Supplementary material for: Digestive contents and food webs record the advent of dinosaur supremacy
Source: Nature. 2024 Nov 27;636(8042):397–403. doi: 10.1038/s41586-024-08265-4 (PMC11634772; doi:10.1038/s41586-024-08265-4)
Supplement: Supplementary file 1 — Supplementary Notes 1–3 and additional references. These contain detailed information about the studied sites, specimens and bromalite inclusions. In addition, figures associated with these notes can be found on figshare (https://doi.org/10.6084/m9.figshare.26103031). [file 41586_2024_8265_MOESM1_ESM.pdf]

nature portfolio

https://doi.org/10.1038/s41586-024-08265-4

Supplementary information

Digestive contents and food webs record the advent of dinosaur supremacy

In the format provided by the authors and unedited

Supplementary Notes for:

Digestive contents and food webs record the advent of dinosaur supremacy

Martin Qvarnström[1]*, Joel Vikberg Wernström[1,2], Zuzanna Wawrzyniak[3], Maria Barbacka[4,5], Grzegorz Pacyna[6], Artur Górecki[6], Jadwiga Ziaja[5], Agata Jarzynka[7], Krzysztof Owocki[8], Tomasz Sulej[8], Leszek Marynowski[3], Grzegorz Pieńkowski[9,#], Per E. Ahlberg[1] & Grzegorz Niedźwiedzki[1,9]*

[1]Department of Organismal Biology, Evolutionary Biology Centre, Uppsala University, Norbyvägen 18A, 752 36 Uppsala, Sweden; [2]The Arctic University Museum of Norway (UMAK), UiT The Arctic University of Norway, Lars Thørings veg 10, 9006, Tromsø, Norway; [3]Institute of Earth Sciences, Faculty of Natural Sciences, University of Silesia in Katowice, Będzińska 60, 41-200 Sosnowiec, Poland; [4]Hungarian Natural History Museum, Botany Department, 1431 Budapest, Pf. 137, Hungary; [5]W. Szafer Institute of Botany, Polish Academy of Sciences, Lubicz 46, 31-512 Kraków, Poland,; [6]Institute of Botany, Department of Taxonomy, Phytogeography and Palaeobotany, Faculty of Biology, Jagiellonian University, Gronostajowa 3, 30-387 Kraków, Poland; [7]Institute of Geological Sciences, Polish Academy of Sciences, Research Centre in Kraków, Senacka 1, 30-002 Kraków, Poland; [8]Institute of Paleobiology, Polish Academy of Sciences, Twarda 51/55, 00-818 Warsaw, Poland; [9]Polish Geological Institute – National Research Institute, Rakowiecka 4, 00-975 Warsaw, Poland

#Deceased April 19, 2023

*Corresponding authors. Email: martin.qvarnstrom@ebc.uu.se; grzegorz.niedzwiedzki@ebc.uu.se

See figshare for all supplementary figures referenced in the supplementary information (Qvarnström et al., 2024)

1. Background

The earliest dinosaur fossils date to approximately 230-240 million years ago and derive from Gondwana, which constituted the southern part of the supercontinent Pangea (Rogers et al., 1993). This Gondwanan record constitutes the basis for most hypotheses concerning the early evolution and radiation of dinosaurs, but the rise of dinosaurs to global ecological dominance was complex, diachronous, and influenced by various palaeoecological factors over a long stretch of time (Brusatte et al., 2008, 2010; Irmis 2010; Nesbitt et al., 2013). To understand the evolutionary success of dinosaurs fully it is, therefore, crucial to study the fossil record across all Pangea, including its poorly explored northern parts.

The Polish basin, an eastern part of the Central European Basin (CEB) (Supp. Fig. 1a, b), constitutes an excellent place for studying the evolution of early dinosaurs due to its rich continuous terrestrial fossil record through the interval of interest. Housing some of the earliest dinosauromorphs/dinosauriforms and dinosaurs, it offers a unique glimpse into the first dinosaur-influenced ecosystems of the northern part of Pangea (Dzik, 2003; Gierliński et al., 2004; Brusatte et al., 2011; Niedźwiedzki et al., 2014; Pieńkowski et al., 2014).

Nine fossiliferous localities, grouped into five fossil assemblages, were used in this study (Supp. Figs. 1-10; Supp. Table 2). All localities are in relatively close geographic proximity (Silesia: Krasiejów/Woźniki, Poręba/Kocury and Lisowice/Marciszów; Holy Cross Mountains: Gromadzice-Rzuchów and Sołtyków/Hucisko; Supp. Fig. 1c; Supp. Tables 2 and 13) and found in a well-constrained sedimentological and stratigraphical context that span across the key interval: the mid-late Carnian (early Late Triassic) to the early Hettangian (earliest Early Jurassic), (Fig. 1 in main text).

We used feeding evidence inferred from more than 500 bromalites, bones with bite marks, comparative anatomy, functional morphology and presumed ecology of ichnotaxon producers to infer diets and feeding behaviours to elucidate the food webs of the vertebrate assemblages recorded in these sites. Bromalites (trace fossils related to food processing) carry fundamental data on the feeding strategies of extinct animals (e.g., Chin, 2002, 2007; Zatoń et al., 2015; Qvarnström et al., 2016; Bajdek et al., 2016, 2017; Niedźwiedzki et al., 2016; Loinaze et al., 2018) and are abundant throughout the Mesozoic.

We studied the bromalites using a multi-proxy approach. After macroscopic observations, the material was selected for more detailed research, which included: morphological description (shape, size, characteristic features), taphonomic description (general mineralogy and state of preservation), description of the contents based on observations of the surface and fractures, synchrotron micro-CT scanning, micro-CT scanning, scanning electron microscopy imaging (SEM), energy-dispersive X-ray spectroscopy (EDS) analyses, microscopic observations of thin sections (MTS), microscopic examinations of residual materials (ME) from dissolution and varied geochemical studies (Supp. Tabs. 3-13). Partial results of some of these studies were presented in previous reports (Bajdek et al., 2014; Zatoń et al., 2015; Qvarnström et al., 2017, 2019a,b; 2021; Barbacka et al., 2022).

Synchrotron microtomography of more than 100 of the most abundant and best-preserved bromalites revealed a wide range of remarkably well-preserved food remains (Fig. 2 in main text; Ext. Data Figs. 1-10). Organic molecules are preserved in many specimens as evidenced by diverse organic inclusions in the synchrotron scans, plant and arthropod cuticles, vertebrate bones, scales, teeth in acid residues/thin sections, and geochemical analyses (Supp. Tables 3-13).

2. Sites and geological sections

2.1. Krasiejów

The roughly 30-meter-thick deposits exposed in the clay-pit Krasiejów near Opole contain a rich record of plant and animal fossils (Supp. Figs. 1c, 2, 10a,f, 11, 14, 15, 32a-c). Bones and bromalites are mainly found in two fossil-bearing intervals, both approximately 1–1.5 m thick (Supp. Fig. 2). The lack of radiometric dates makes precise dating of the deposits difficult. However, the vertebrate fauna (e.g. temnospondyl and phytosaur), invertebrates (e.g., conchostracans, bivalves), fossils of freshwater algae (charophytes), and plant macrofossils are compatible with a mid to late Carnian age (Zatoń et al., 2005; Dzik and Sulej, 2007; Pacyna, 2014, 2019; Lucas, 2015). There are also different opinions regarding the stratigraphic position of these deposits (see Szulc et al., 2015). The Krasiejów section has been included to the lower Norian part of the recently definied the Patoka Marly-Sandstone Member of the Grabowa Formation (Szulc et al., 2015). Initial identification of the palynomorphs and plant cuticles from the bromalites collected from bonebeds at Krasiejów indicates that these represent palynotaxa widely known from the Carnian of the CEB (on-going study).

The fossil assemblage from Krasiejów represents a mix of terrestrial and lacustrine ecological communities (Fig. 1 in main text; Supp. Table 2). The freshwater community consisted of charophyte algae, molluscs (including abundant bivalves and rare gastropods), arthropods (including a crab-like cycloid crustacean, ostracods, conchostracans, other poorly-preserved arthropods), the phytosaur *Paleorhinus* sp., two temnospondyls (*Metoposaurus krasiejowensis* and *Cyclotosaurus intermedius*), the large lungfish *Ptychoceratodus roemeri*, the hybodont shark *Lonchidion* sp. and various actinopterygian fish that still await proper description (Pawlak et al., 2022). The terrestrial fauna includes insects (beetles), small diapsids (e.g., sphenodontids), the gliding archosauromorph *Ozimek volans*, the dinosauriform *Silesaurus opolensis* (Supp. Fig. 10f), the aetosaur *Stagonolepis olenkae*, and the large carnivorous ‘rauisuchid’ *Polonosuchus silesiacus*. The microvertebrate remains witness of a remarkably diverse faunal assemblage, including hybodont sharks, actinopterygians (palaeoniscids, semionotids, redfieldiids), dipnoans, temnospondyls, eucynodonts (*Polonodon woznikensis*), rhynchocephalian lepidosauromorphs, and various small archosauromorphs (Kowalski et al., 2019; Sulej et al., 2021). Ganoid, cycloid and ctenoid fish scales were found in the lower bone-bearing horizon (Antczak and Bodzioch, 2018). A few tetrapod tracks were also found in Krasiejów. These are poorly preserved but show ressemblance of the ichnogenera *Brachychirotherium* (pseudosuchian tracks) and *Atreipus* (dinosauriform tracks), (Supp. Table 2).

Numerous well-preserved bromalites (more than 200 specimens) were collected from the two fossiliferous intervals at Krasiejów (Supp. Figs. 14, 15; Supp. Table 3). Fossil bones with bite marks from predators or scavengers were also found (Supp. Fig. 32a-c). In the examined collection of over 300 bone specimens, only six specimens showed traces of such damage. Despite their rarity, they suggest that large predators, likely the apex predator *P. silesiacus*, exploited bone (on-going study), which is also suggested by the presence of a few bone-rich regurgitalites (Ext. Data Fig. 2).

Furthermore, a poorly preserved flora is known from the locality and it is dominated by voltzialean conifers, and transportable, resistant seeds (Supp. Fig. 11) (Dzik and Sulej, 2007; Pacyna, 2014, 2019). Other gymnosperms, ferns, and sphenophytes are rare and typically very poorly preserved (Pacyna, 2019). One sphenopsid species, *Neocalamites merianii*, was identified. It is preserved as impressions, flattened casts, moulds and isolated leaves. So far, only one small

fern specimen, determined as *Sphenopteris* sp., has been found. The major collection of plant macroremains of this site has not yet been described in detail, but was mentioned only on a preliminary basis (Dzik and Sulej, 2007; Pacyna, 2014, 2019). All plants were transported prior to deposition and thus did not grow at the immediate vicinity of the place of burial. So far, the host rocks from the fossil-bearing deposits have not yielded any palynomorph finds, but numerous spores and pollen grains have been found in the bromalites (on-going study), which can be explained by their unique preservational capacity of bromalites (e.g., Qvarnström et al., 2016, 2019, 2021).

2.2. Woźniki

The Woźniki clay-pit near Częstochowa (Supp. Fig. 1c) is historically one of the oldest known fossil vertebrate-bearing sites in Silesia (Sulej et al., 2011, 2020). The terrestrial deposits from the site are similar to other Middle Keuper deposits of the CEB, dated to the Carnian (e.g., Shukla et al., 2010) or Norian (e.g., Szulc et al., 2006, 2015). The strata exposed in the Woźniki clay-pit are composed of red and green claystones and siltstones (Supp. Fig. 3). Several layers of carbonate grainstones and carbonaceous sandstones occur between these fine-grained siliciclastics. Most of the carbonate-rich beds are very thin, apart from two thick lenticular layers. The sections belong to the recently definied Grabowa Formation, but there is currently no consensus on the exact position of this section within the formation. Szulc et al. (2015) correlated the fossiliferous section from the Woźniki clay-pit with those known from Poręba, Lisowice and Marciszów (Lisowice bone-bearing level) but the rich palaeontological record from these sites contradicts this interpretation (see below).

The Woźniki biota (Supp. Figs. 3, 10a, 16) (Sulej et al., 2011, 2020) is structurally very similar to that of Krasiejów (Supp. Table 2). It includes hybodont sharks (e.g. *Nemacanthus* sp.), the temnospondyl *Cyclotosaurus* sp., the archosauromorph *Ozimek* sp., a phytosaur, an aetosaur, a silesaurid (*Silesaurus* sp.), the eucynodont *Polonodon woznikiensis*, and an unidentified large predatory archosauriform (Sulej et al., 2011, 2020). The most complete tetrapod specimen from Woźniki, however, belongs to the medium-sized kannemeyeriiform dicynodont *Woznikella triradiata* (Sulej et al., 2011; Szczygielski and Sulej, 2023). In contrast, no dicynodont remains have so far been described from Krasiejów. Moreover, numerous tetrapod tracks and traces (including dinosauriform footprints) are associated with the skeletal fossils at Woźniki (Supp. Table 2). Sulej et al. (2011) initially studied these tracks but new material has been found since this study was published.

The age of the Woźniki assemblage remains partly unresolved due to a lack of data from palynomorphs and/or radiochronology. However, the age of this site can be assessed based on faunal finds. Sulej et al. (2011) considered the locality to be of mid-late Carnian age based on the presence of the conchostracan *Laxitextella* cf. *laxitexta* (see Olempska, 2004), and freshwater bivalves similar to *Silesunio parvus* from Krasiejów. The currently recognized temporal range of *Laxitextella laxitexta* covers major part of the Carnian in the CEB and does not stretch into post-Carnian strata (see Geyer and Kelber, 2018). In Woźniki vertebrate taxa such as *Polonodon, Ozimek, Cyclotosaurus* and *Silesaurus* occur, which indicates that it is a faunal assemblage similar to the one from Krasiejów, and therefore probably of the same age.

The presence of *Woznikella* demonstrates that dicynodonts were part of the mid–late Carnian terrestrial ecosystem in the CEB, which is also supported by findings from the Stuttgart Formation (middle Carnian) of Germany (Schoch, 2012). The geographical proximity and age similarity of

Woźniki and Krasiejów suggest that dicynodonts likely were a part of the fauna at Krasiejów as well, although no remains are found at the site so far.

Only 15 rather poorly preserved bromalites were found in Woźniki clay-pit (Supp. Table 4; Supp. Fig. 16). They are morphologically similar to the specimens from Krasiejów, and they also contain palynomorphs, which will be included in additional palaeobotanical and biostratigraphical research.

2.3. Poręba

The sedimentology and stratigraphy of the Poręba site near Zawiercie were described by Sulej et al. (2012) and Niedźwiedzki et al. (2014). The site is located in the northern part of the Poręba town, ca. 1 km N from the town (Supp. Figs. 1c, 4). This area represents the easternmost part of the Upper Silesian subbasin and the western margin of the Jura Krakowsko-Częstochowska region. The lithostratigraphical position of the section is essentially equivalent to that of mid–upper Middle Keuper strata found in the Niwki and Poręba boreholes, both located close to the site (Szulc et al., 2006). These sections belong to the recently definied Patoka Member of the Grabowa Formation, formerly known as Zbąszynek Beds (Sulej et al., 2012; Niedźwiedzki et al., 2014; Szulc et al., 2015; Zatoń et al., 2015; Szczygielski and Sulej, 2016; Bajdek et al., 2019).

Palynological studies support a mid–late Norian age for the Poręba fossil assemblage. More than 50 specimens of palynomorphs were identified in one organic carbon-rich sample from the layer located just above the uppermost bone bed (Niedźwiedzki et al., 2014). This palynoassemblage is characteristic for Subzone IVb of the *Corollina meyeriana* Zone (see Orłowska-Zwolińska, 1983, 1985) in the Polish part of the CEB and is characteristic of the lower–middle part of the Zbąszynek Beds and the Jarkowo Beds from Polish Lowlands (Orłowska-Zwolińska, 1983, 1985).

The deposits from Poręba differ remarkably from the strata in the Krasiejów and Woźniki sections. They consist of thick, interdigitated layers of grey mudstones and (grey, white, or yellow) conglomerates of fluvial origin, all of which contain numerous and well-preserved plant remains (Supp. Fig. 4). The claystone-rich intervals are enriched in organic remains (e.g., dispersed plant cuticles and charcoal). The dominant plant species are sphenophytes and conifers of *Brachyphyllum*- and *Pagiophyllum*-like leafy shoots (Supp. Table 2).

The Poręba site contains a diverse faunal assemblage (Supp. Table 2) with bivalves, fish (hybodont sharks, actinopterygians, sarcopterygians), temnospondyls (only a few finds), turtle (numerous bones), aetosaur (a few bones and dermal scutes), unidentifid archosauriform (a few fragmentary bones) and relatively diversified dinosauriform/dinosaur fauna including a silesaurid, a *Tawa*-like herrerasaurid and a neotheropod (Niedźwiedzki et al., 2014; and on-going study). Turtle remains (*Proterochersis*) dominate the fossil record of the site and represent over 80% of the tetrapod bone fossils (Sulej et al., 2012; Niedźwiedzki et al., 2014; Szczygielski and Sulej, 2016).

Numerous bromalites were found in a few fossiliferous horizon (Supp. Fig. 17; Supp. Table 5), and well-preserved specimens come from the mudstone-claystone intercelations. Only some of the collected bromalites have been analyzed (Bajdek et al., 2019), but an additional dozen or so specimens collected from the newly exposed rocks indicate that their diversity is even greater than what has been published so far. In total, this material includes 41 bromalite specimens (Supp. Table 5) and constitutes important material from the stratigraphic interval where the first dinosaurs (a *Tawa*-like herrerasaurid and a neotheropod) occur in the Late Triassic record of Poland.

2.4. Kocury

The Kocury site near Dobrodzień (Supp. Figs. 1c, 5) is located in Silesia, 40 km north-east of Opole (Skawiński et al., 2017; Czepiński et al., 2021). The recently excavated 3-meter-thick section (Supp. Fig. 5) is composed of (red, green, and grey) claystones and mudstones, which are interrupted by up to 1.5 m thick grey polymictic conglomerates, with organic remains, including bone fragments (Czepiński et al., 2021). The matrix preserved at the proximal tip of the theropod or neotheropod *Velocipes guerichi* von Huene, 1932, a specimen collected from Kocury in the 19th century (von Huene, 1932; Skawiński et al., 2017), is similar to the conglomerates exposed in a newly excavated section.

The stratigraphic position of the Kocury section is poorly determined, but lithostratigraphic correlation and finds of vertebrate remains indicate that it is probably an age equivalent to the Poręba assemblage, and thus probably mid-late Norian in age. Czepiński et al. (2021) argued, based on simple observations and lithostratigraphic criteria that the Kocury section possibly belongs to the Patoka Mudstone-Sandstone Member of the Grabowa Formation. The composition of vertebrate assemblage from Kocury highly resembles that of Poręba (see Supp. Table 2). Both assemblages yield remains of dipnoan fish, proterochersid turtles, theropod or neotheropod dinosaurs and typothoracin aetosaurs (Sulej et al., 2012; Niedźwiedzki et al., 2014; Czepiński et al., 2021), strongly suggesting that both sites represent records of equivalent faunas, perhaps very similar stratigraphic age.

So far, no bromalite fossils have been found in this site, but this fauna enriches our knowledge of the mid to late Norian vertebrate faunal complex in the region.

2.5. Lisowice

The abandoned Lipie Śląskie clay-pit is located at the village Lisowice near Lubliniec in Silesia (Supp. Fig. 1c). A 12-meter-thick section used to be exposed in the site but is today largely covered by water (Supp. Fig. 6). Vertebrate bones, tracks, and bromalites occured in numerous beds within two bone-bearing intervals. Most bones derive from the upper interval (several subhorizons) and are usually preserved in lenticular bodies of carbonate-rich siltstones and mudstones, or in carbonate nodules. The lower bone-bearing interval seem to show different type of fossil preservation and record a different fauna, but has not been sufficiently studied, and is thus not considered here.

Determining the stratigraphic position of the upper bone-bearing interval from the Lisowice section has sparked discussion (see Dzik et al., 2008a, b; Świło et al., 2014; Pieńkowski et al., 2014; Szulc et al., 2015; Fijałkowska-Mader et al., 2015; Kowal-Linka et al., 2019; Sulej and Niedźwiedzki, 2019). According to the published stratigraphical interpretation, the upper bone-bearing interval at Lisowice represent Woźniki Limestone/Woźniki Formation of Norian age (Szulc et al., 2006), the Middle Norian part (Patoka Mudstone-Sandstone Member) of the Grabowa Formation, a newly definied lithoformation (Szulc et al., 2015) or correlate with uppermost Norian/?lowermost Rhaetian upper part of Zbąszynek Beds and Rhaetian Wielichowo Beds from the Polish Lowlands (Pieńkowski et al., 2014; see also Deczkowski, 1997; Franz et al., 2007a, b; Franz, 2008; Franz et al., 2021).

A published U-Pb dating of a single detrital zircon crystal (Ion Microprobe SHRIMP IIe/MC), recovered from the conglomeritic bed (positioned below the upper fossil-bearing interval, but in the upper part of the lower bone-bearing interval; see Fig. 2), yielded an absolute age of 211 ± 3 Ma (Kowal-Linka et al., 2019). The boundary between the Norian and Rhaetian stages is currently defined at the age of ~208.5 million years (Cohen et al., 2023). The zircon date falls within the

208–214 Ma range, thus suggests not older than late Norian/earliest Rhaetian age for the upper part of the lower bone-bearing interval (see Sulej and Niedźwiedzki, 2019; on-going study). Alternativly, if the Norian–Rhaetian boundary age were to be taken as ~205.5 Ma, then the age of the youngest zircon, would point to the middle Norian (Alaunian–Sevatian boundary at ~209–210 Ma) or late Norian (Alaunian–Sevatian boundary at ~214–216 Ma) as the maximum depositional age of the upper part of the lower bone-bearing interval (Kowal-Linka et al., 2019).

The upper bone-bearing interval in Lisowice is rich in plant remains (Supp. Fig. 12; Supp. Table 2). These are mostly represented by palynomorphs (miospores, pollen grains and megaspores), dispersed plant cuticles, but include also more well-preserved plant macrofossils (wood fragmnents, twigs, whole leafs of fragments, cones). The occurrence of plant fossils in Lisowice was first noted in the early 1980s (Fuglewicz and Śnieżek, 1980; Marcinkiewicz, 1981) but only megaspores were preliminarily described. Recent studies on this locality have yielded many new specimens and undescribed observation (Staneczko, 2007; Ociepa et al., 2008; Dzik et al., 2008a, b; Marynowski and Simoneit, 2009; Wawrzyniak and Ziaja 2009, 2010; Wawrzyniak, 2010a, b, c, d, 2011; Jarzynka and Wawrzyniak, 2012; Świło et al., 2014; Pieńkowski et al., 2014; Warzyniak, 2023; on-going study). The dominant plant fossils in Lisowice are conifers. Collected conifer material includes numerous wood fragmnents (*Agathoxylon*), sterile leafy twigs and male and female cones. In general, four types of foliage can be distinguished – scale-leaf (*Pagiophyllum*, *Brachyphyllum* and *Hirmeriella* type), and needle-leaf type resembling *Elatocladus*. There has been no natural attachment found so far between sterile shoots and reproductive organs, therefore their certain taxonomical identification is hard to determined. Additional plant fossils from Lisowice are represented by other conifers, cycadophytes, ginkgophytes, and pteridosperms (Supp. Table 2; on-going study). Many collected fossils have no cuticle preserved or only residual cuticle which is brittle and difficult to obtain for microscopic studies. Sediments contain also a diversity of isolated generative plant parts - seeds and seed scales of uncertain affinities and isolated cone-like structures. A few putative Lycophyta shoots were found in the assemblage.

There is much greater morphological and taxonomical diversity present among dispersed cuticles. A major component of *cuticulae dispersae* are conifers. Separated scale leaves like *Pagiophyllum*, *Brachyphyllum* and *Hirmeriella* are very abundant, but they show bigger variance in cuticle morphological details than what can be observed from macrofossils. Most of the cuticles are fragmentary, i.e. cuticles of needle-like conifer very rarely have preserved leaf base, which makes it difficult to reconstruct their attachment to a stem, a feature use for taxonomical recognition. Preliminary observations reviled the presence in *cuticulae dispersae* material of cuticles resembling ginkgoalean, cycads and seed ferns. A few cuticles were distinguished resembling *Lepidopteris ottonis* (Staneczko, 2007; Wawrzyniak, 2010d), seed fern characteristic for Rheatian, but the cuticles are rare and very fragmentary, which make identification rather ambiguous.

Comparable floras are known from other middle Norian to late Rhaetian and earliest Jurassic assemblages of the southern Poland (Pacyna, 2014). The flora from Lisowice is surprisingly diversified (Supp. Table 2), is still being studied, and some finds indicate its similarity to younger Rhaetian or even Early Jurassic floras from Greenland and Skåne (Harris, 1932, 1935, 1937). Rich and well-preserved conifer fossils from Lisowice, show obvious affinity to some taxa from other Late Triassic Silesian floras (Pacyna, 2014).

The Lisowice site has been subject to several palynological investigations. Staneczko (2007) identified a miospore assemblage resembling the Subzone IVb *Corollina meyeriana* Zone. Subsequent studies by Dzik et al. (2008a, 2008b) noted the presence of *Brachysaccus*

neomundanus, *Monosulcites* cf. *M. minimus* and *Ricciisporites* cf. *R. tuberculatus*, indicating a Rhaetian age for the site. Świło et al. (2014) listed palynotaxa from Lisowice and based on that determined strata as the uppermost Norian–lower Rhaetian, equivalent of the uppermost Zbąszynek Beds and lower Wielichowo Beds from the Polish Lowlands. This conclusion was supported by Pieńkowski et al. (2014), who suggested that this palynoassemblage would represent the Subzone IVc of the *Corollina meyeriana* Zone (defined by the appearance of *Rhaetipollis germanicus* in Orłowska-Zwolińska 1983, 1985), and the *Ricciisporites tuberculatus* Zone, which was incorporated into the Rhaetian *Rhaetipollis germanicus* Zone (Kürschner and Herngreen, 2010). A palynological investigation conducted by Fijałkowska-Mader et al. (2015) assigned the miospore spectrum from Lisowice to the Subzone IVb *Corollina meyeriana* representing middle to late Norian age (Fijałkowska-Mader, 2015). Based on presence of the *Rhaetipollis germanicus* Fijałkowska-Mader (2015) have correlated the subzone IVc with lower part of the Rhaetian *R. germanicus* zone (see Kürscher and Herngeen, 2010).

An earliest Rhaetian age of the upper fossil assemblage is indicated by the presence of the conchostracans *Gregoriusella polonica* Kozur, Niedźwiedzki and Sulej, 2010 (Kozur and Weems, 2010; Pieńkowski et al., 2014), the occurrence of isoëtalean macrospores *Trileites* cf. *pinguis* and *Horstisporites bertelseni* (Fuglewicz and Śnieżek, 1980), the first occurence of *Rhaetipollis germanicus* pollen (Świło et al., 2014; and on-going study), finds of numerous cuticle and leaf fragments of the seed-fern *Lepidopteris ottonis* (Staneczko, 2007; Ociepa et al., 2008; Dzik et al., 2008a, b; Wawrzyniak and Ziaja 2009; Wawrzyniak, 2010a, b; Świło et al., 2014; Zatoń et al., 2015; Pieńkowski et al., 2014) or its fructification (*Peltaspermum rotula*), as well as the presence of a diversified dinosaur fauna (bones and footprints; see Sulej and Niedźwiedzki, 2019 and on-going study) and double-rooted, early mammaliaform (*Hallautherium* sp.) teeth (Świło et al., 2014).

The vertebrate fossil assemblage from the upper bone-baring interval consists of many terrestrial tetrapods, aquatic temnospondyls and numerous fish (Dzik et al., 2008b) (Supp. Tab 2; Supp. Fig. 10). During ten seasons of excavations at this site, several thousand fossil specimens were collected. The most common bones are of a giant dicynodont (*Lisowicia bojani*), followed by archosauromorph and temnospondyl bones (Dzik et al., 2008a,b; Niedźwiedzki et al., 2012; Sulej and Niedźwiedzki, 2019). The diversity of small to medium-sized terrestrial (or semi-aquatic) reptilians is relatively high as indicated by the presence of numerous isolated bones of 1) two species of ?pterosaurs/flying reptiles – known from cranial elements, limb bones, vertebrae and teeth (pterosaur ?Pterosauria and gliding archosauromorphs, ?Sharovipterygidae); 2) two species of early dinosaurs, including a supposed early ornithischian – represented by cranial elements, vertebrae, limb and pelvic bones (Saurischia and ?Ornithischia); 3) two species of small predatory dinosaurs – known from cranial elements, limb and pelvic bones (Theropoda A and B); 4) small crocodylomorph limb bones (Crocodylomorpha); 5) a pachystropheus-like reptile – identified from vertebrae and limb bones (Thalattosauria); 6) lepidosauromorphs – known from skull bones, limb bones and teeth (Sphenodontia) and numerous isolated bones and teeth of other small, but still unidentified, diapsids (Diapsida indet.). This assemblage also includes the remains of therapsids, in form of isolated teeth and postcranial elements of Mammaliaform (*Hallautherium* sp.) and eucynodont (Cynodontia). Temnospondyls (*Cyclotosaurus* sp. and *Gerrothorax* sp.) are known from isolated skull bones, partial and complete jaws and numerous limb bones or bony dermal elements. The top predator in this assemblage is represented by the 5 to 6-meter long theropod-like archosaur *Smok wawelski* (Supp. Fig. 10e; see Niedźwiedzki et al., 2012). The fish fauna is identified based on numerous disarticulated fossils and includes a medium sized hybodont

shark (fin spines), a large coelacanth (skull elements and isolated scales), medium to large dipnoan fish (mainly skull bones and tooth plates of *Ptychoceratodus* sp.) and diverse small sized actinopterygians (skull bones and numerous scales, lepidotrichia). The vertebrate microfossils and small fossils are dominated by remains of aquatic vertebrates and comprises primarily actinopterygian scales (or other dermal elements) and teeth, along with teeth and dermal denticles of the hybodont (Hybodontiformes) and rhomphaiodon (Synechodontiformes) sharks (Zalewski et al., in prep.). In addition, there are rare tiny teeth of different diapsids/archosauromorphs as well as fossils of early anurans in the fossil material of the site (Niedźwiedzki et al., 2015).

A rich tetrapod track material (comprising more than 100 specimens) is known from the locality and it bears evidence of dinosaur dominated fauna with small to large therapsid and small diapsid/crocodylomorph tracks (on-going study). The dinosaur fauna inferred from the tracks (Supp. Fig. 10d) include theropods of different sizes (*Grallator*, *Anchisauripus*, *Kayentapus*, *Eubrontes*, cf. *Eubrontes* ichnogenera) and small ornithischian dinosaur (*Anomoepus*). This track assemblage reflects notable dinosaur diversity, not known from Upper Triassic localities in the Upper Silesia subbasin.

Numerous bromalites (Supp. Figs. 18, 19) were found in several fossiliferous horizon in the upper bone-bearing interval (Bajdek et al., 2014; Zatoń et al., 2015; Qvarnström et al., 2019a) and well preserved and rich material comes from the mudstone-siltstone-claystone intercelations (Supp. Fig. 6; Supp. Table 4).

2.6. Marciszów

The Marciszów site (also known as Zawiercie-Marciszów) is located near Zawiercie in the easternmost part of the Upper Silesian subbasin, close to the margin of the Jura Krakowsko-Częstochowska (Supp. Fig. 1c). The geological section that once outcropped there is now inaccessible since it was buried during the development of the landfill. The section represents the Patoka Mudstone-Sandstone Member of the Grabowa Formation (Szulc et al., 2015) and is dominated by fluvial fine-grained siliciclastics, mainly grey or red mudstones and siltstones (Supp. Fig. 8b). Poorly sorted conglomeratic layers occur as lenses and contain oncoids, coalified wood fragments, charcoal accumulations, plant remains, bivalves, and isolated vertebrate bones and teeth. These erosional-bound layers are intimately linked to the preservation of fossil bones at Marciszów section (Budziszewska-Karwowska et al., 2010; Szulc et al., 2015; Niedźwiedzki and Budziszewska-Karwowska, 2018). Carbonate concretions and carbonate-rich mudstones and conglomerates often contain rich plant and vertebrate remains, as well as palynomorphs (e.g., *Corollina meyeriana*, *Ovalipolis ovalis*, *Brachysaccus neomundanus*, *Enzonalasporites* sp.; see Sadlok and Wawrzyniak, 2013; Fijałkowska-Mader et al., 2015). The Marciszów vertebrate assemblage (Supp. Table 2) is relatively rich and includes remains of fish (sharks, actinopterygians, sarcopterygians), a large dicynodont (cf. *Lisowicia*), a large theropod-like archosaur *Smok* sp. (Budziszewska-Karwowska et al., 2010; Niedźwiedzki and Budziszewska-Karwowska, 2018), tetrapod trace fossils (Sadlok and Wawrzyniak, 2013), and bones with preserved bite marks (Budziszewska-Karwowska et al., 2010; Sadlok, 2022). The similar biotas from Marciszów and Lisowice indicate the existence of a specific fauna with large herbivores (giant dicynodonts) and predators (theropod-like archosaurs) in the Upper Silesian subbasin in the latest Triassic (see Niedźwiedzki and Budziszewska-Karwowska, 2018). The sections in Lisowice and Marciszów represents records of equivalent faunas, and bone beds from Marciszów are equivalents to the upper bone-bearing level exposed at Lisowice (Dzik et al., 2008a,b; Niedźwiedzki et al., 2012).

Recent studies of plant fossils from Marciszów (Wawrzyniak and Filipiak, 2023) proved the presence of gymnosperms, including the arborescent conifer *Agathoxylon* cf. *keuperianum*, sphenophytes represented by stems of *Equisetites* sp., and *Neocalamites* sp., but analysis of cuticles, macerated from different plant-bearing lithologies, revealed a variety of conifer foliage (e.g. needle-leaf *Elatocladus*-type and scale-leaf *Pagiophyllum-Brachyphyllum*-like).

A few bromalites were found in Marciszów in *ex situ* material accumulated after the large-scale digging operations (Supp. Table 6).

2.7. Gromadzice-Rzuchów

Tracks and bone-bearing beds were identified in two uppermost Triassic sites in the Kaminonka River Valley in the north-western margin of the Holy Cross Mts. (Supp. Figs. 1c). These poorly exposed sections are located in deep ephemeral stream valleys, representing very similar sedimentological sequences and are considered herein as a collective Gromadzice-Rzuchów site (Supp. Fig. 7). The fossiliferous beds are a few meters thick and composed of white to yellowish sandstones with dark, brown to grey, siderite-rich mudstone intercalations, and local beds of grey to greenish claystone and brownish sandy mudstone with thin coal layers. These deposits have been considered as Rhaetian *sensu polonico* or were included to the so-called Rhaeto-Liassic combaine sequence (Samsonowicz, 1929; Jurkiewiczowa, 1953; Kopik, 1970; see also Fijałkowska, 1999). The study of similar lithostratigraphic units from other parts of the Mesozoic margin of the Holy Cross Mts. suggests an early to mid Rhaetian age for these deposits (Pieńkowski, 2004a). Early Jurassic sedimentation in this region was preceded by conspicuous erosion (Pieńkowski, 2004a), which removed much or all of the Rhaetic sediments. Jurkiewiczowa (1967) reported buried palaeovalleys of rivers from the Przedbórz area, which were cut down to 60 m into Upper Triassic (Norian) red bed deposits. However, near the depocenter of the basin (the area near Parszów between Starachowice and Skarżysko-Kamienna) the hiatus in the uppermost Triassic embraces only late Rhaetian, as shown by palynological data (Marcinkiewicz, 1957; Orłowska-Zwolińska, 1962; Fijałkowska, 1989) and sedimentological interpretations (Pieńkowski, 2004a). Several observations and some paleontological finds from the eastern part of the Mesozoic margin of the Holy Cross Mts. (Ostrowiec Świętokrzyski area) indicate that the situation there is slightly different, and that a quite large package of Rhaetian sediments is preserved locally under the erosional boundary of the Lower Jurassic. This grey and greenish sequence reaches several meters thickness and is unconformably resting on red and brownish Norian deposits. This sequence is fossiliferous, contains plant remains and is interpreted here as the middle to late Rhaetian in age. This is supported by palaeontological finds (see below).

The two track-bearing levels recognized in the Gromadzice-Rzuchów section (Supp. Table 2) yield large sauropodomorph tracks (*Evazoum* and *Tetrasauropus*-like tracks), archosauromorph or crocodylomorph tracks (*Chirotherium*-like tracks), small ornithischian dinosaur tracks (cf. *Anomoepus*) and small to large-sized tridactyl theropod dinosaur tracks (*Grallator*, *Anchisauripus*, *Kayentapus* and *Eubrontes*). Rare finds of bone fragments suggest that some temnospondyls were still present (cf. *Cyclotosaurus*), but no other characteristic Late Triassic elements (rauisuchians, phytosaur, aetosaur or their tracks) are known from the middle to late Rhaetian deposits of the Gromadzice-Rzuchów site.

The coal-bearing mudstones and debris-rich, grey claystones contain plant macrofossils (Supp. Table 2). The dominant plant remains in the Gromadzice-Rzuchów sections are dipteridacean ferns (*Dictyophyllum*), seed ferns (*Lepidopteris*), ginkgophytes (*Czekanowskia*), and conifers (*Hirmeriella*), and *Podozamites*-like forms. Comparable floras are known from other

middle to late Rhaetian assemblages in the CEB. This sequence represents a poorly understood Upper Triassic interval in the Polish Basin and has a high correlation value. Already planned studies of this sequence will allow us to better understand the position of the Triassic-Jurassic boundary in the Holy Cross Mts.

Only seven bromalite specimens (Supp. Table 7) have been found in this site.

2.8. Hucisko

A small outcrop of Triassic-Jurassic transitional alluvial deposits was discovered in Hucisko in 2014 (Supp. Fig. 1c). The fossiliferous beds in this site are located just above an erosional boundary with the Upper Triassic (?late Norian) reddish to brown mudstones/siltstones and are represented by a one a half meter-thick section of grey to greenish mudstone, locally interrupted by white, soft claystone intercalations and with beds of brownish sandstones with numerous charcoal fragments (Supp. Fig. 8a). A latest Rhaetian-earliest Hettangian age is indicated by basic sequence stratigraphic correlation of the basalmost part of the Snochowice beds which are covering the sequence (Pieńkowski, 2004a), palaeobotanical finds (e.g., *Hirmeriella muensteri*, *Thaumatopteris brauniana*, *Czekanowskia* sp.), and fossils of conchostracans *Bulbilimnadia kilianorum* (see Kozur and Weems, 2010) collected from the dark, grey claystone intercalations locaited above the bone-bearing interval in Hucisko. The plant fossils and the especially presence of *B. kilianorum* (see Lucas and Tanner, 2015) indicate that this fossil assemblage represents the latest Rhaetian-earliest Hettangian biota and that this short sequence is similar in age to the strata exposed in the lower part of the Sołtyków clay-pit (Gierliński et al., 2004; Niedźwiedzki, 2011; Pieńkowski et al., 2014; Barbacka et al., 2022). Field observations from the region indicate that it may be a locally preserved, non-eroded patch of Triassic-Jurassic transitional strata. Palynological studies of these deposits are planned, it is also possible that these strata represent the earliest Hettangian, which has interesting implications for determining the age of the Snochowice beds.

The recovered tetrapod material includes over 30 bones, bone fragments, and teeth. Several finds of bones and teeth from Hucisko (Supp. Table 2) indicate the presence of large-sized theropod and sauropodomorph dinosaurs (on-going study). The material is fragmentary, but it is the first location of this type in the Holy Cross Mts. region where an accumulation of bones was found in sediments from the Triassic-Jurassic transitional interval.

There are rare, but well preserved bromalites in the bone-bearing layers at Hucisko, and several (16) specimens were obtained for study (Supp. Table 8).

2.9. Sołtyków

The outcrop at Sołtyków near Skarżysko-Kamienne (Supp. Fig. 1c) is an abandoned clay-pit, nowadays protected as a nature reserve (geology, palaeontology and nature). It is located on the north-central Mesozoic margin of the Holy Cross Mts. and comprises a sequence of alluvial-plain deposits formed in the uppermost Rhaetian-lower Hettangian interval (Pieńkowski, 2004a,b; Niedźwiedzki, 2011; Pieńkowski et al., 2014). The strata represent a siliciclastic coal-bearing lithofacies formed in an alluvial wetland, with deposition controlled by high-sinuosity, anastomosing streams (Supp. Fig. 9). The strata witness of a climate that was warm to temperate, with wetter and drier seasons (Pieńkowski, 2004a,b; Pieńkowski et al., 2014). An earliest Hettangian age of the middle to upper part of the section is indicated by sequence stratigraphic correlation (Pieńkowski, 2004a, b), plant fossils (Reymanówna, 1992; Wcisło-Luraniec, 1991; Barbacka et al., 2010, 2022), palynomorphs (Ziaja, 2006), and the conchostracan *Bulbilimnadia kilianorum* (Pieńkowski et al., 2014). The Sołtyków section can be correlated with the Niekłań

PGI-1 borehole (200 m depth) located some 4 km to the north-east (Pieńkowski et al., 2014; Brański, 2014), which yielded a core spanning the Norian-Rhaetian to Hettangian section (Pieńkowski et al., 2014). From 158.9 m depth and upwards, a rich assemblage of characteristic earliest Jurassic palynomorphs appears, including a FAD (first-appearance datum) of the index pollen grain *Cerebropollenites thiergartii* Schulz, 1967 at 158 m depth. The strata between ~165 m and ~135 m approximately correspond to the Sołtyków outcrop (Pieńkowski et al., 2014; Barbacka et al., 2016), and the interpretation of clay mineral data from this interval (Pieńkowski et al., 2014; Brański, 2014) can be correlated from Niekłań to Sołtyków.

A rich ichnological record of vertebrates (comprising more than 500 tetrapod tracks and 300 vertebrate bromalites) is known from the locality and it bears evidence of a terrestrial fauna dominated by various dinosaurs. The dinosaur fauna inferred from the footprints (Supp. Fig. 10b,c) include theropods of different sizes (cf. *Stenonyx*, *Grallator*, *Anchisauripus*, *Kayentapus*, *Eubrontes*, cf. *Megalosauripus*), two ornithischians (*Anomoepus*, *Delatorrichnus*), and large sauropodomorphs (cf. *Tetrasauropus*, cf. *Otozoum*, *Parabrontopodus*), (see Gierliński et al., 2004; Niedźwiedzki, 2011; Pieńkowski et al., 2014). The trace fossil assemblage reflects notable dinosaur diversity, not known from pre–Early Jurassic localities in the region (Pieńkowski et al., 2014). Body fossils of vertebrates are rare in Sołtyków and occur mainly in the lowermost beds in the section (Niedźwiedzki, 2011). Actinopterygian fish scales and teeth were collected from clayish, organic-rich lake deposits. Bones of a basal crocodylomorph (found in a large bromalite), turtle carapace fragment and some dinosaur remains (numerous fragmentary bones) were found in sandy facies in the lower and upper part of the exposed section (Niedźwiedzki, 2011; on-goin study). Some bones from the upper part of the section are preserved as natural casts.

The Sołtyków locality provides a unique ichnological data for determining biodiversity of small non-dinosaurian tetrapods including early mammals and eucynodonts (cf. *Ameghinichnus* isp., and *Dicynodontipus* isp. tracks), turtles (unnamed tracks), lepidosauromorphs (*Rhynchosauroides* isp.), pterosaurs (cf. *Pteraichnus*) and early crocodylomorphs (*Batrachopus* isp. and unnamed tracks). Numerous dinosaur nests with eggs have been found at the site, including eggs with embryo remains and accumulations of eggshells, nests with eggs, rare finds of bones, and abundant bromalites (Pieńkowski, 1998, 1999, 2004a, b; Niedźwiedzki, 2011; Qvanström et al., 2022).

Plant fossils indicate that the richness of the vegetation associated with the main vertebrate-bearing deposits in Sołtyków was moderate (Barbacka et al., 2007, 2010, 2022), with so far about 33 recognised species (Supp. Fig. 13; Supp. Table 2). The species *Hirmeriella muensteri*, *Podozamites* sp., and *Neocalamites lehmannianus* dominate the assemblage. Lycophytes are rare and only one species was described based on macroremains (*Odrolepis liassica*). Ferns are uncommon but taxonomically diverse, with the following recognised taxa: dipteridaceous ferns - *Thaumatopteris brauniana*, *Goepertella microloba* and *Dictyophyllum* sp., osmundaceous *Todites princeps*, matoniacaeous *Phlebopteris angustiloba.* Fern rhizomes are common, but their precise affiliation is uncertain. Seed ferns are represented by leaves of *Pachypteris lanceolata* and *Sagenopteris nilssoniana* and seeds of *Caytonia* sp. Bennettitaleans are represented by *Otozamites brevifolius* and *Pterophyllum* sp.; cycads by *Paracycas minuta*; conifers, besides dominant *Hirmeriella muensteri*, by *Podozamites* cf. *schenkii*, *Podozamites* sp. and *Swedenborgia* sp. Rare ginkgoaleans are known only from the female cone *Schmeissneria microstachys*, probable gnetaleans by the male cone *Piroconites kuespertii.* The numerous plant cuticles were found in bromalites (Barbacka et al., 2022). These finds complement our knowledge about Sołtyków flora and represent seed ferns (*Komlopteris distinctiva*), cycadophytes, ginkgophytes and conifers. A

fragment of a needle leaf, *Aciphyllum triangulatum*, very similar to the leaves of *Pinus*, was also found in a bromalite (Barbacka et al., 2022).

The palynomorph assemblage from Sołtyków described based on samples collected from the middle part of the exposed succession is dominated by *Classopollis*, *Aratrisporites*, *Concavisporites* and *Cyathidites* (Ziaja, 2006). The Sołtyków palynoassemblage is very similar to the composition of the *Concavisporites-Duplexisporites-Aratrisporites* (= Lias α1 and α2) zone from Franconia in Germany and *Pinuspollenites-Trachysporites* zone from northwestern Germany and south Scandinavia (Lund, 1977; Achilles, 1981; Ziaja, 2006). Nuerous palynomorphs were found in bromalites from Sołtyków (Barbacka et al., 2022). More than 20 palynotaxa of fossil spores and pollen grains were determined in herbivore and carnivore bromalites.

Numerous bromalites, more than 300 specimens, were found in four fossiliferous horizon in Sołtyków section (Supp. Figs. 20-26; Supp. Table 9; see also Barbacka et al., 2022).

3. Bromalites

3.1. Bromalites and feeding traces from Krasiejów

Bromalite specimens were collected *in situ* from the two fossil-bearing stratigraphical intervals (Supp. Fig. 2) across several sections that were well exposed (in the artificial exposure) at Krasiejów from 2009 to 2012. All specimens, collection of 127 specimens (Supp. Table 3), were photographed and are housed in the Insitute of Paleobiology, Polish Academy of Sciences, Warsaw under the collection series ZPAL AbIII. In total, more than 100 specimens were selected for additional more detailed studies. These bromalites were compared and grouped into morphotypes (M1-M7) based on shape, size and appearance (Supp. Table 3). All specimens were screened for inclusions at outer and broken surfaces. Sixty well-preserved bromalites (Supp. Table 3) were scanned using propagation phase-contrast synchrotron microtomography (PPC-SRμCT) at the European Synchrotron Radiation Facility (ESRF) in Grenoble, France.

A few dermal armour plates and long bones belonging to the aetosaur *Stagonolepis* bear evidence of bite marks produced by a large predator/scavenger (Supp. Fig. 32a-c). Although aetosaur bones co-occur with remains of several other tetrapod taxa in this site, preliminary observations suggest that bite marks are restricted to aetosaur remains, which suggests that these archosaurs were relatively often preyed on by a large predator. A presumed regurgitatlite with mass accumulation of highly fragmented dermal elements, possibly belonging to *Stagonolepis*, was also found in this site. Whether predated or scavenged, these damaged elements indicate that *Stagonolepis*, despite being well armoured, constituted prey for a relatively large carnivore. The most likely candidate to have produced all the bite marks and the regurgitalite is the large rauisuchian *Polonosuchus*.

The results of the examination (SEM, EDS, MTS, ME, and results from dissolution) of all collected specimens are summarized in Supp. Table 3.

3.1.1. Synchrotron-scanned bromalites from Krasiejów

Six relatively big coprolites (Supp. Fig. 15; Ext. Data Fig. 1) appear to have faint large-scale spirals (one or two big coils), and at least one of the specimens (ZPAL AbIII/3401) has inclusions that are internally organized in a spiral-like fashion (Ext. Data Fig. 1a). This group of bromalites is comprised of six complete specimens, as well as possibly a fragmentary one. Coprolite ZPAL AbIII/3401 contains crushed bivalves, a semi-articulated actinopterygian fish (probably a redfieldiid) including hundreds of scales, fins, and a pelvic girdle in approximately life position

(Ext. Data Fig. 1) (Qvarnström et al., 2017). Specimen ZPAL AbIII/3413 contains numerous bivalves (with preserved umbo, ligament and growth lines), a few ostracods, a poorly-preserved fish scales and many unidentifiable inclusions (Ext. Data Fig. 1). Bromalite ZPAL AbIII/3412 contains numerous nodules, but not any identifiable inclusions (Ext. Data Fig. 1b). Specimen ZPAL AbIII/3415 contains various fish remains, mostly scales, and a highly (secondary) mineralised system of cracks (Ext. Data Fig. 1e). ZPAL AbIII/3414 is relatively devoid of identifiable inclusions, although there are a few scattered fish bones and a flat, possible plant cuticle (Ext. Data Fig. 1f). Specimen ZPAL AbIII/3416 contains numerous fish scales, one beetle elytron, bivalves (although mainly found in the adjacent matrix) and many fragments of round seed-like structures with complex internal structure (Ext. Data Fig. 1d). The producer of these bromalites was a relatively big durophagous animal that fed on benthic bivalves and fish. The most likely producer known from the locality is the big lungfish *Ptychoceratodus roemeri*, which is known from numerous tooth plates associated with vomerine teeth and scales (Skrzycki, 2015). Tooth marks matching the dental plates of dipnoans have been previously described from bivalve shells in Lisowice (Gorzelak et al., 2010).

Two fragments of a relatively big specimen (Ext. Data Fig. 1g) are composed of various rounded “clasts”, and many bone fragments. The matrix does not appear to be calcium phosphatic in contrast to other coprolite specimens. Instead, the matrix is similar to that of the host rock, suggesting it might be a regurgitalite rather than a coprolite (Gordon et al., 2020). It contains plant cuticles (Ext. Data Fig. 1h,i), tooth-bearing temnospondyl skull fragments, vertebrae, enigmatic bones with big vascular canals, fish remains (some appearing only in specific clasts) and many rounded clasts (some could be degraded bones, other contains small inclusions including elytra, and others fish remains). The producer of this bromalite targeted various aquatic and terrestrial prey (fish, temnospondyls and tetrapods) and judging by the size of the inclusions (and the entire accumulation), it was a fairly large predator. The most likely producer is the rauisuchian *Polonosuchus*.

Eleven spherical specimens that range from 19 to 36.5 mm by their largest diameter constitute a relatively big part of the synchrotron-scanned bromalites from Krasiejów (Ext. Data Fig. 2). Despite their similar morphology and appearance, these bromalites contain quite different inclusions. Fish remains are present in at least four of the specimens. Other recognisable inclusions comprise two articulated beetles, an isolated elytron, unidentifiable arthropod fragments, scattered small bones, an ostracod, plant remains, and many abundant spherical structures. The spheres are very similar to those find in bromalites of other morphologies (e.g., bromalites attributed to *Silesaurus*) and may represent mineralised voids after gas escape. It cannot be excluded, however, that some spherical structures represent parasite eggs although the resolution does not permit a proper identification of these. The producers of the spherical bromalites were feeding on fish, arthropods and, perhaps, also plant material in aquatic environments. Temnospondyls represent likely candidates to have produced the droppings as 1) both spherical bromalites and body fossils of temnospondyls are very common in the locality, 2) oval fish-bearing bromalites have been found associated to temnospondyl material in other localities (e.g. Niedźwiedzki et al., 2016), and, 3) temnospondyls are aquatic to semi-aquatic and functional morphology suggest that they were piscivorous.

There are numerous elongated bromalites from Krasiejów (Ext. Data Figs. 3-9). These are of various size and the length-to-width ratio differ much between specimens. Some are post-depositionally flattened, and others are round in cross section. Elongated droppings are produced

by a large number of animals (Hunt and Lucas, 2012a, b), and these bromalites were likely produced by different animals.

Five complete or near-complete elongated specimens (ZPAL AbIII/3402, 3408–3411) have characteristic irregular surface structures – the specimens are grey to brown and have a thin, smooth coating. They are 31 to 54.5 mm in length and 16 to 22 mm in maximum diameter. These specimens have previously been described and assigned to the dinosauriform *Silesaurus opolensis* (Qvarnström et al., 2019b, 2021). One of the bromalites, specimen ZPAL AbIII/3402, contains many beetle remains including lots of small elytra, two large ones, several pronota and two tibiae (Fig. 2; Ext. Data Fig. 5c) (Qvarnström et al., 2019b). Small elytra and partial beetle bodies were found in the other bromalites of the morphotype, altogether indicating that the bromalite producer(s) voluntarily ingested relatively small insects. The size of the bromalites suggests that the producer itself was not a small animal. The best candidate from the body fossil record of the same site (the Carnian locality) Krasiejów is the dinosauriform *Silesaurus opolensis*. *S. opolensis* displays some interesting feeding adaptations, including protrusions of the dentary (and probably premaxilla) that formed a beak. Qvarnström et al. (2019b) hypothesized that the beak was used to peck insects of the ground and/or to root in the litter much like modern birds. An insect diet has previously been hypothesised for non-dinosaurian dinosaurimorphs, but not silesaurids (Langer et al., 2013). At least one of the bromalites assigned to *Silesaurus* contains a fish scale (Ext. Data Fig. 5e). Fish remains are also found in morphologically quite similar bromalites. Thus, it seems likely that the diet of *Silesaurus* also incorporated fish. Although this morphotype only contains bromalite with insect remains as the only identifiable inclusions, there are other bromalites in the analysed assemblage with insect remains as well, and some with fish and elytra.

Another big elongated specimen (ZPAL AbIII/3440) contains fish scales, various tooth plates, bones, and spherical egg-like structures (Ext. Data Fig. 3e). This specimen, along with another big fish-bearing elongated one (Ext. Data Fig. 3a) were likely made by the phytosaur *Paleorhinus*. The rest of the big elongated and/or flat specimens were likely produced by different animals, although it is difficult to say which. ZPAL AbIII/3429 and ZPAL AbIII/3431 contain fish and beetle remains (Ext. Data Fig. 2). ZPAL AbIII/3432 contains scarce fish remains and a structure with denticles (Ext. Data Fig. 2m). In contrast, ZPAL AbIII/3430 has a peculiar matrix, devoid of major inclusions, which contains numerous invertebrate trace fossils in form of burrows (Ext. Data Fig. 2o). Some were likely produced by *Silesaurus* and *Paleorhinus* but perhaps also by *Stagonolepis*, *Polonosuchus*, or animals not yet known from the body fossil record.

Two elongated and anisopolar (one end is blunt and the other slightly tapered) spiral specimens contain an abundance of fish remains. These include numerous fish scales and fin lepidotrichia still in articulation ZPAL AbIII/3509 (Ext. Data Fig. 6a). Specimen ZPAL AbIII/3508 contains lots of fish remains, but also a few arthropod remains, including beetle elytra and a possible arthropod pincer or mandible (Ext. Data Fig. 6b). Possible producers for these two bromalites are quite big aquatic animals with a spiral gut valve, perhaps the coelacanths which are known from the body fossil record (Dzik and Sulej, 2007).

There are additionally three small spiral bromalites (ZPAL AbIII/3510-3512) that range from six to ten millimeter in maximum diameter. Two of the bromalites are elongated, and the third is bullet shaped (Ext. Data Fig. 6). The inclusions are completely dominated by various scales and bones of actinopterygian fish (Ext. Data Fig. 6). The bromalites also contain a few ostracods, and specimen ZPAL AbIII/3511 what seems to be a seed with internal structures preserved (Ext. Data Fig. $6h_v$). These objects were probably accidentally ingested during predation events. A segmented structure very similar to tapeworm proglottids is present in ZPAL AbIII/3510, which could

represent the oldest cestode in the body fossil record (Ext. Data Fig. 6i). The producer of these bromalites was an animal that fed on actinopterygians and which possessed a spiral gut valve. The spiral valves of extant non-teleost actinopterygian fishes generally feature a higher number of turns than those of lungfish do and also exhibit a greater range (Argyriou et al., 2016). Considering the small size of the bromalites (compared to the scroll specimens) and relatively large number of spirals, actinopterygians, or possibly hybodont sharks, which both are known from body fossils (Dzik and Sulej, 2007) are likely producers of these bromalites. It is unclear if the ornamented bone in specimen ZPAL AbIII/3511 derives from a small temnospondyl or an actinopterygian fish. If the former is true, it suggests that juvenile individuals of *Metoposaurus* or *Cyclotosaurus* were vulnerable to attack from large ganoid fishes or hybodonts.

There are two small elongated specimens without spiral structure in the collection that are morphologically very similar (Ext. Data Fig. 6). Bromalite ZPAL AbIII/3513 contains numerous ostracods and specimen ZPAL AbIII/3514 contains ostracods, fish scales, a charophyte gyrogonite, bivalves, and possible arthropod cuticle (Ext. Data Fig. 6f). Two other small specimens. Two small bromalites are very small and slightly droplet or bullet-shaped. It contains a lot of small inclusions although they are not identifiable. ZPAL AbIII/3516 contains numerous invertebrate burrows and ZPAL AbIII/3515 hardly any identifiable inclusions except an ostracod shell (Ext. Data Fig. 6e).

3.1.2. Amber-like preservation of insects

In recent years, it has become evident that bromalites may act as microenvironments in which inclusions can be more well-preserved than in the host rock itself (Seilacher, 2001; Qvarnström et al., 2016). Degradation-prone inclusions such as soft tissues can occur in bromalites, and in deposits with generally 2D-preserved fossils, bromalites can host 3D-preserved inclusions. Much of the fossil record of insects consists of more or less 2D-preserved specimens, and exceptionally preserved ones in amber (Smith and Marcot, 2015). The extent of amber in the geological record, however, is strongly biased towards certain intervals. Here, we describe a number of beetles from Triassic bromalites and show that insects can be as well preserved in bromalites as in amber. Numerous beetle elytra, appendages, and articulated or semi-articulated beetle specimens are preserved in the bromalites from Krasiejów. The preservation of insects in bromalites is also biased, in terms of these being targeted as prey (or accidently ingested, post-colonisation), and especially small specimens will have the best chance of surviving the gastrointestinal digestive system tract of the producer (e.g. Fig. 2d in main text; Ext. Data Figs. 2-7). Nevertheless, these extraordinarily-preserved specimens are important since they provide information on the ecology of the beetle-eaters, but also of the occurrence and evolutionary history of beetles. An extraordinarily preserved insect head has been described from the Late Triassic of Australia (Northwood, 2005) and are present in Late Permian bromalites from Russia (unpublished data).

3.2. Bromalites from Woźniki

In total, 15 registered bromalites have been collected from the Woźniki clay-pit, and all specimens are poorly preserved making their contents difficult to study. All specimens are housed in the Insitute of Palobiology, Polish Academy of Sciences, Warsaw (ZPAL Woźniki Cop 1-15). All specimens were collected from the bone-bearing lower and upper intervals, which was exposed at the clay-pit from 2007-2009 (Supp. Fig. 3). The bromalites were divided into three morphological groups (M1-M3) based on size and internal content (Supp. Table 4). Bromalites from Woźniki have not been synchrotron or CT scanned. Their contents are known from visual inspection. The

likely bromalite makers are sharks (small spiral bromalites), sarcopterygians (larger spiral bromalites) and small to medium-sized tetrapods (oval-shaped non-spiral bromalites), which suggests that the diversity of aquatic vertebrates was higher than known from the body fossil record. The results of the examination (SEM, EDS, MTS, ME, and results from dissolution) of selected specimens are summarized in Supp. Table 4.

3.3. Bromalites from Poręba
Bromalite specimens in Poręba, in total 46 specimens, have been found in the conglomerate and mudstone intervals and are associated with micro- and macroremains of plants and bone fragments. All bromalites were catalogued (see collection ZPAL V.39), are housed in the Insitute of Paleobiology, Polish Academy of Sciences, Warsaw and selected specimens were photographed (Supp. Fig. 17). Several bromalite specimens from this locality, classified in the different morphotypes, were recently studied (Zatoń et al., 2015; Bajdek et al., 2019). Here we are taking into consideration both this already known material and new specimens that have not been studied so far (20 additional specimens). Bromalites collected from Poręba were grouped by Bajdek et al. (2019) within four morphotypes (A, B, C, and D) attributable to sharks, medium-sized omnivorous or carnivorous tetrapods (likely turtles), sizable carnivorous archosaurs (likely theropods) and an indeterminate big herbivorous tetrapod (possibly dicynodonts or aetosaurs), respectively. In our studies, we use new terminology (M1-M6) and this previously proposed scheme by adding two additional morphotypes of carnivore tetrapods (Supp. Table 5). Bromalites from Poręba have not been synchrotron or CT scanned. The results of the examination (SEM, EDS, MTS, ME, and results from dissolution) of all collected specimens are summarized in Supp. Table 5.

3.4. Bromalites and feeding traces from Lisowice
In total, 231 registered and over 50 unregistered bromalite specimens have been collected from the upper bone-bearing interval, in several exposed sections, which were accessible at Lisowice (Lipie Śląskie clay-pit) from 2007 to 2017 (Supp. Fig. 6). All bromalites were studied (see collection ZPAL V.33) and selected specimens were photographed (Supp. Figs. 18, 19). More than 200 collected specimens were studied under binocular microscope, compared based on shape, size, general content, and appearance (e.g. surface textures, bromalite matrix content). All specimens were measured and screened for organic remains at the surface and on broken areas. Measurements and information on all bromalite morphotypes (M1-M8) are found in Supp. Table 6.

Plant remains are preserved in large, oval-shaped, dark, carbon-rich bromalites. These are attributed to the gigantic, toothless dicynodont *Lisowicia* that indiscriminately fed on a wide range of plants while accidentally swallowing mineral grains. The bromalite mass contains wood fragments, higher plant cuticles, disaggregated higher plant tracheids and black amorphous organic particles. Plant cuticles are the most numerous components of the bromalites (Supp. Figs. 27-30). Cuticles, even fragmentary ones, are well preserved and lack distinctive traces of degradation from digestion. Most cuticles are from conifers, including the abundant *Brachyphyllum* and *Pagiophyllum*-type of foliage. It is possible, however, that some nutritious plants were completely digested by the gastrointestinal system of *Lisowicia* and thus never ended up in the droppings. The bromalite specimens provide direct evidence on the diet of the extinct large therapsid (megaherbivore) and suggest that occasionally received detrital resources. The results of the examination (SEM, EDS, MTS, ME, and results from dissolution) of all collected specimens are summarized in Supp. Table 6.

3.4.1. Synchrotron-scanned bromalites from Lisowice

Eighteen complete or fragmentary phosphatic bromalites were synchrotron scanned at the ESRF, France (Ext. Data Figs. 8-10). Three out of totally ten big elongated bromalites were scanned and showed to contain various fragmented bones and crushed serrated teeth (Supp. Fig. 19; Ext. Data Fig. 8; Qvarnström et al., 2019b). In addition, there are several regurgatilites, which also contain bone fragments (but slightly larger ones than in the coprolites), and isolated bones of dicynodonts with bite marks (Qvarnström et al., 2019b). All these bromalites and bite marks were most probably made by *Smok* and suggest that it was an osteophagous top predator that fed on *Lisowicia bojani*, temnospondyls, fish, and other archosaurs (Qvarnström et al., 2019b).

Other scanned elongated bromalites that lack a spiral structure include two specimens with fish remains and three bromalites with plant remains (Ext. Data Figs. 9). The fish-bearing bromalites was likely produced by a small theropod and the plant-rich ones by ornithischian-like dinosaurs (both known from the body and trace fossil record of the site). CT scans of fragments from possible dicynodont bromalites display numerous plant remains. Bromalites with spiral structures include large amphipolar (spirals evenly distributed across the specimens) bromalites and small heteropolar (spirals confined to one end of the bromalites) ones. The two scanned amphipolar specimens (ZPAL V.33/1202 = COP06 and COP18 = ZPAL V.33/1207; see Supp. Table 6) contain various actinopterygian inclusions and were likely produced by lungfish (Ext. Data Figs. 10). Small heteropolar bromalites also contain actinopterygian remains but were likely produced by the hybodont sharks which are known from numerous microremains.

3.4.2. Dicynodont bromalites from Lisowice

More than 200 specimens (registered and unregistered) of more or less irregular, spherical or oval-shaped bromalites, which all contain plant remains, were collected or observed in the field. These bromalites range from 3 to 17 cm in diameter and has been classified as dicynodont feces (Bajdek et al., 2014). Investigated bromalites were found in the strata rich in plant fossils, like charcoalified tree trunks, coalified compressions of shoots and separated plant parts (i.e. cones, leaves, seeds). The sediment also contains dispersed cuticles (*cuticulae dispersae)*, sometimes forming thin layers of concentrated plant detritus. Herbivorous and carnivorous bromalites with cuticles preserved inside them, are another source of plant remains.

Their morphology is variable; some of them have original, not eroded surfaces and clear cut edges. All specimens are ovoid to spheroidal in shape, usually dark grey and organic rich distinctly different from the host rock and other remains from the site, including bones, phosphatic bromalites of predatory tetrapods, oncoids, inorganic carbonate nodules and septarian concretions or plant remains. All studied bromalites lack bone and other animal remains. Most of the collected bromalites contain small mineral particles, mainly quartz grains and inorganic clasts. The bromalites are in places highly mineralized by pyrite, but the main diagenetic alteration is represented by calcite microspary replacement. Several bromalites contain highly fragmented and macroscopically visible plant material. In total, 3 organic-rich fragments of bromalites ZPAL V.33/1107-1109 (labelled Bromalite 1-3; see Supp. Table 6) were selected from the Lisowice collection and dissolved in with 5% HCl acid and the undissolved fraction was examined.

The HCl-resistant minerals and particles (including authigenic mineralization of pyrite) and organic carbon of one of the studied carbonate-rich bromalite comprise about 30–50% of the mass of the bromalites. The vast majority of clasts are represented by very small quartz grains (between 0.5–0.05 mm based on SEM observations) and also very large grains of sand-sized particles are not quartz grains or pyrite crystals. Numerous specimens contain visible plant tissues (cuticle

fragments and wood fragments), palynomorphs (pollen and spores) and other accumulations of amorphous and dark organic carbon. Two types of organic carbon remains (cuticle fragments and amorphous, dark organic matter) were examined to characterize the carbon and nitrogen isotopic composition of residue and to identify the diet of the bromalite producers. All the investigated samples are phosphate-free and contain a large amount of authigenic sulphide minerals (mainly pyrite), sometimes in the form of characteristic crystal nests, sphere-like objects or are disposed concentrically. In a few bromalites, mineral particles are concentrated in the form of small spherical structures (secondary grains) and some pyrite minerals show pseudomorphs after microbes, probably coccoid bacteria.

All studied specimens were found in organic-rich, grey mudstone and siltstone exposed in the middle and upper part of the section. Most of the collected specimens are fragmented and incomplete; some specimens have been partially reconstructed by fitting broken pieces together. The eight specimens subjected to destructive analysis were first photographed and measured. Four thin sections from partially preserved and organic-rich specimens were made for study of internal structures and possible food remains.

Plant material from dicynodont bromalites

Plant remains preserved in dicynodont bromalites comprise wood fragments, higher plant cuticles, disaggregated higher plant tracheids, and black amorphous organic particles. Higher plant cuticles constitute the most abundant component. The term cuticle here relates to the extracellular layer which overlies the plant epidermis and other cutinised plant parts (i.e., cutinised megaspore membrane). A few miospores were found attached to cuticles surface or trapped between lower and upper cuticle. Cuticles preserved in bromalites are fragmentary. The degradation of the cuticles varies, but it is mainly mechanical degradation rather than damage caused by digestive fluids (Bajdek et al., 2014). Because of the fragmented nature of the cuticles definite identification could not be done. Therefore, cuticles were grouped into morphotypes and when possible, identified to the lowest taxonomical level possible. There are a different number of cuticle specimens in each bromalite residue, but most of the morphotypes are present in each bromalite.

Every cuticle piece was counted as a separate specimen and has a specific number assigned with regard to the bromalite it originates from. The character of the material that can be considered as dispersed plant cuticle limits the description to the structure of the cuticle. Because the gross morphological features could not be determined, in most cases the taxonomical determination is difficult. Therefore, cuticles were grouped into morphotypes and when possible, identified to the lowest taxonomical level.

Specimens from all three studied palaeobotanically bromalites were grouped into morphotypes on the basis of combination of cuticle characters that are consider to have taxonomical importance among gymnosperms (e.g., Harris, 1935; Oldham, 1976; Kerp, 1990; Karasev, 2013): the differentiation of adaxial and abaxial leaf side, the presence of stomata, the orientation, arrangement and density of stomata, the shape and structure of stomata complex, the shape and arrangement of other epidermal cells, the ornamentation of epidermal cells and the presence of trichomes. Firstly, the most complete specimens were selected and described, preferably with upper and lower cuticle preserved and the shape complete enough to determine which plant fragment it represents, and if possible determining leaf shape. Specimens that are more fragmentary were later compared and assigned into morphotypes. The types are characterized by a following alphabet letters (Supp. Figs. 27-30).

Measurements

The investigated material consists of 159 single cuticles from 3 bromalites: 73 cuticles in bromalite 1, 49 cuticles in bromalite 2 and 37 cuticles in bromalite 3 (Supp. Table 6). Measurements of cuticles length, width and surface area were taken using a NIS-Elements software. Length of the cuticle was measured according to the arrangement of epidermal cells, that are indicating a top and a base of an organ (leaf, seed etc.), between the most distal ends. Width of the cuticles was measured perpendicular to epidermal cells rows and the length. Because of the irregular shapes of the cuticles, surface area needed to be estimated using a NIS-Element tool „ellipse" for measuring surface.

The mean length of the cuticles is 2,81 mm, ranging from 0,27 to 10,02 mm. The mean width is 1,85 mm, minimum is equal 0,22 and maximum is 7,53 mm. The mean cuticle surface area is 3,46 mm^2 whereas the biggest area measured is 60,16 mm^2 and the smallest is 0,07 mm^2.

Cuticle identification

Cuticle type A (Supp. Fig. 27:1-3)

Description. Separate leaves assigned to type A has a lanceolate shape and are slightly incurved on adaxial side, with an apex tapering to an acute. A leaf base is usually not preserved or is incomplete. The biggest most complete leaf cuticle is 4,1 mm long and 2,5 mm wide, but the biggest cuticle fragment assigned to type A is 8,0 mm long and 3,6 mm wide. Leaves has a short wing on a ridge formed by elongated marginal cells (Supp. Fig. 27:1). Cuticles on both sides of a leaf are similar.

Epidermal cells are rectangular to polygonal, arranged in longitude rows and are shorter and more isodiametric in stomata rows. Anticlinal walls of epidermal cells are straight and evenly cutinised, slightly rounded in corners. Periclinal walls bear solid papillae in the center of a cell or closer to the acroscopic side.

Stomata are present on both surfaces. Stomatal complexes lay irregularly in parallel longitudinal rows, single stomata wide, separated with c. 1-7 rows of epidermal cells (Supp. Fig. 27:2). Circular to oval in outline stomata complexes are composed of a pair of sunken guard cells and 4-6 subsidiary cells. The size of stomata complexes warries between 46 and 74 mm diameter across. Apertures are randomly orientated. Subsidiary cells are stronger cutinised than epidermal cells, each cell bears a solid papilla on the inner edge, overarching the stomatal pit. In most of the specimens, subsidiary cells have thickened anticlinal walls forming a ridge (Supp. Fig. 27:3). Outer parts of periclinal walls show fine striations. Encircling cells are inconspicuous and incomplete.

Type present in all three bromalites.

Discussion. Cuticles of Type A probably belong to the family Cheirolepidiaceae due to the presence of the thickened rim with papillae surrounding the stomatal pore. Type A specimens resemble *Pagiophyllum* Heer 1881, a foliage morphogenus use for sterile leaves/shoots of conifers from Late Triassic to Upper Cretaceous. Isolated awl-shape leaves fit to the description of *Pagiophyllum* in Harris 1979, Kendall 1948,. Fragmentary cuticles classified into type A differ between each other, but all show characters of the genus. Differences might indicate presence of more than one species of *Pagiophyllum* or be a result of heterophylly in *Pagiophyllum.* Nevertheless, the dispersed and fragmentary nature of the material do not allow more accurate determination of the cuticles. Among *cuticulae dispersae* macerated from the sediment from Lipie Śląskie/Lisowice morphogenus *Pagiophyllum* is very abundant.

Material. Bromalite 1: 04 (fragment), 08 (fragment, epidermal cells more elongated), 09 (fragment), 13, 14, 15 (incomplete leaves), 16 (fragment, papillae more distinctive), 20 (fragment

with margin and base), 21(fragment), 27 (fragment, poorly preserved), 28/29 (fragment), 30 (fragment), 35 (fragment, longer papillae), 36 (fragment with margin), 38 (fragment), 46 (fragment, cells and papillae elongated), 47 (fragment, ridge wing present on the margin), 49 (small fragment, poorly preserved), 50 (fragment), 52 (fragment), 55 (fragment with margin, poorly preserved), 59 (fragment), 69 (fragment), 71 (fragment), 74 (fragment)

Bromalite 2: 02 (fragment, epidermal cells shorter), 03 (fragment, anticlinal walls more visible, stomata present), 04 (fragment, fragment with tip margin), 12 (fragment, conspicuous encircling cells), 16 (fragment, shorter cells), 17 (leaf, no base), 18 (fragment), 19 (isolated leaf, poorly preserved), 26 (fragment, epidermal cells shorter), 27 (fragment with margin, subsidiary cells cutinised only on the inner side, forming papillae), 33 (leaf, no base, no tip), 35 (fragment with margin), 38 (fragment with margin), 39 (fragment, papillae smaller, not on every cell), 48 (leaf), 50 (leaf)

Bromalite 3: 01 (leaf no base, no tip), 02 (leaf, no base), 03 (fragment, cuticle thinner, epidermal cells shorter), 04 (fragment, cuticle slightly thinner), 06 (leaf no base, no tip), 07 (fragment), 08 (fragment, epidermal cells shorter), 10 (fragment with margin), 16 (fragment, slightly longer epidermal cells), 17 (leaf, base incomplete), 25 (fragment with margin), 26 (leaf, fragmentary, no base, no tip), 27 (fragment),

Cuticle type B (Supp. Fig. 27:4-6)

Description. The most complete cuticles assigned to type B morphotype have preserved upper and lower cuticle. Leaves were ovate in proximal view and almost as wide as long, with apex from acute to obtuse (Supp. Fig. 27:4). Leaf base is not preserved. The biggest most complete leaf cuticle is 5,2 mm long and 5,8 mm wide. The smallest leaf is 1,5 mm length and 1,6 mm width.

Epidermal cells, arranged in longitudinal rows converging toward the apex (Supp. Fig. 27:4), are rectangular to polygonal, usually isodiametric. Anticlinal walls are thick and straight. Periclinal walls do not show any papillae. Cells are elongated closer to the margin and are forming a scarious, dentate edge on the free part of a leaf (Supp. Fig. 27:5). Towards the leaf tip area epidermal cells are getting elongated and are equipped with long papillae forming a "hairy-like" apex cover. There are usually 3-4 epidermal cell rows between stomata rows.

Round stomata complexes aligned in rows single stomata wide have sunken guard cells surrounded by 5-7 subsidiary cells. Stomata are separated between each other usually by 3-4 epidermal cells within the row, even when stomata are adjacent, they do not share a subsidiary cell (Supp. Fig. 27:6). The stomata complex size range from ca. 50 to 90 mm of diameter across. Subsidiary cells are strongly cutinised, forming a characteristic ridge, each subsidiary cell has a solid papilla pointed towards the center of stomatal complex.

Type present in all three bromalites.

Discussion. Despite differences in shape of an apex within individual cuticles and incomplete preservation, type B cuticles show similarities to *Brachyphyllum* Lindley & Hutton ex Brongniart, a morphogenus possessing features characteristic for Cheirolepidiaceae family. Isolated cuticles correspond with the description of Kendall (1947), Watson (1988), Ash (1999). The presence of macrofossils with cuticles preserved assigned to genus *Brachyphyllum* identified from the Lipie Śląskie/Lisowice sediments (Wawrzyniak 2010a), makes the diagnosis more reliable.

Material:

Bromalite 1: 01 (fragment, with apex), 02 (leaf), 03 (leaf, apex acute, attenuate), 10 (dark markings on the periclinal walls, on some epidermal cells sculpturing is visible on the periclinal walls), 11 (fragment),12 (no tip),23 (fragment), 24 (no tip), 33 (fragment, poorly preserved), 37

(fragment), 42 (fragment), 43 (fragment, very thick anticlinal walls), 44 (fragment, poorly preserved), 45 (fragment), 48 (fragment), 51 (fragment, margin), 53/54 (fragment, margin), 58 (fragment, tip), 62 (fragment, tip), 65 (fragment), 77 (fragment)

Bromalite 2: 09 (fragment), 10 (leaf, apex rounded, obtuse), 11 (fragment), 13 (apex acute), 14 (fragment), 15 (fragment, apex acute), 20 (apex acute), 23 (apex acute), 24 (fragment), 25 (fragment), 29 (apex acute, attenuate), 30 (fragment, margin), 31 (apex obtuse), 32 (apex acute, attenuate), 34 (apex acute), 36 (fragment), 37 (fragment), 40 (fragment, very thick anticlinal walls), 41 (fragment, tip), 42 (fragment, margin), 43 (fragment), 44 (fragment, margin), 45 (leaf), 46 (fragment, margin), 47 (fragment, tip), 49 (fragment)

Bromalite 3: 05 (apex acute), 12 (fragment), 13 (fragment), 14 (upper and lower cuticles, adaxial?, stomata on both sides avoiding middle part and upper part, seems to be a bit thinner), 19 (fragment, acute tip with scarious edge, subsidiary cells with more distinctive papillae and thicker walls), 21 (fragment), 22 (apex acute), 23 (apex acute), 24 (fragment), 28 (apex acute, some stomata without papillae on subsidiary cells), 29 (apex acute), 35 (fragment), 37 (fragment)

Cuticle type C (Supp. Fig. 27:7-9)

Description. Cuticle fragments representing only one side of a leaf. None of the cuticles assigned to type C has preserved tip or leaf base. The most complete leaf cuticle in this type have preserved only small fragment of leaf margin. The biggest preserved cuticle is 5,5 mm long and 4,5 mm wide.

Cells are arranged loosely in longitudinal rows. Epidermal cells are rectangular, elongated in rows between stomata and more isodiametric in stomata rows. Marginal cells form a slightly scarious leaf ridge. Anticlinal walls are thickened with rounded corners, sometimes irregular pitting and gentle bulging is present. Periclinal walls are smooth, sometimes striae are visible.

Stomata are organised in loose rows single stomata wide (Supp. Fig. 27:7).

Stomata in rows are placed more or less regularly; sometimes stomata are adjacent but they never share a subsidiary cell (Supp. Fig. 27:8). Stomata aperture and guard cells are sunken. Stomata orientated randomly. Rounded subsidiary cells, from 4-7 (usually 5 or 6) together form a „flower" shape stomatal complex. Stomata complexes size is between 45-85 mm diameter across. Subsidiary cells tapering to an aperture of stomata can vary in size. Their anticlinal walls are slightly more cutinised than surrounding epidermal cells and getting thicker towards the pit. Each subsidiary cell possess a small, prominent papillae pointing toward the center of the complex, often overarching the aperture (Supp. Fig. 27:9). Encircling cells are inconspicuous, but if recognizable they do not form a complete circle.

Type present in all three bromalites.

Discussion. Cuticles of type C most probably represent Coniferales. They are similar to type A and B in construction of the stomata complex and general pattern of the epidermis. They show characters that would correspond with the description of *Brachyphyllum* and *Pagiophyllum* from Harris (1979) and Kendall (1948). Despite that, cells of type C are slightly bigger and do not possess any papillae on periclinal walls as in type A. Also, the distribution of the stomata seems to be more loose than in types A and B. From the shape of the cuticles, it might be assumed that leaf was wider at the base and was tapering towards apex resembling *Brachyphyllum*. Similar cuticles were found in the dispersed material from this locality previously (Wawrzyniak 2010a).

Material:

Bromalite 1: 07 (fragment), 18 (fragment, stomata rows discontinuous), 19 (fragment), 26 (fragment), 32 (fragment)

Bromalite 2: 28 (leaf fragment, small fragment of margin)

Bromalite 3: 30 (fragment, lower part of a leaf)

Remarks on type A, B and C. Cuticles of the same characteristics as assigned to morphotypes A, B and C are also found dispersed in the same strata at Lipie Śląskie-Lisowice clay-pit, and the most complete leaves can be correlated with leafy shoots of two conifer genera – *Pagiophyllum* and *Brachyphyllum*, abundant in the Upper Triassic sediments of this locality. Foliage of these types is known from the Upper Triassic and has been recognized in Cupressaceae, Podocarpaceae, Araucariaceae, Cheirolepidiaceae (Meyen, 1987; Stockey, 1994) and Patokaeaceae (Pacyna et al., 2017). *Pagiophyllum*- and *Brachyphyllum*-type foliage can as well originate from the same species of plant, i.e. in *Patokaea* Pacyna, Barbacka et Zdebska. leaves are morphologically variable, some leaves have extended free part, showing similarity to *Pagiophyllum*, whereas other are in *Brachyphyllum*-type (Pacyna et al., 2017). Unfortunately, the fragmented nature of the specimens and lack of organic attachments with reproductive structures unable the definite identification.

The majority of cuticles from Lipie Śląskie-Lisowice show similarities to plants of the Cheirolepidiaceae family (i.e. random orientation of stomata, 4-6 subsidiary cells and distinctive papillae on them (Watson 1988, Ash 1999, Bomfleur et al 2011).

Cuticle type D (Supp. Fig. 27:10-12)

Description. Leaf fragment with partially preserved upper and lower cuticles. The leaf was probably tapered, one natural margin preserved. Both surfaces of a leaf are similar, but stomata are more dense on one side (abaxial?). Leaf margin is smooth (Supp. Fig. 27:10). Epidermal cells are polygonal, sometimes rectangular, arranged in longitudinal loose rows. In stomata rows epidermal cells are shorter. Anticlinal walls are irregularly cutinised, show thickenings, granulations and pitting. Striae and reticulate patterns can be spotted on periclinal walls, but there are no distinctive papillae present. No resin bodies were found.

Stomata are placed irregularly in longitudinal, loose rows. Stomata complexes are round or oval in outline, typically 45-65 mm diameter across. Stomata guard cells are sunken and strongly cutinised, orientated more or less parallel to cell rows (Supp. Fig. 27:11). The guard cells are surrounded by 4-6 (usually 5) subsidiary cells. Subsidiary cells are rather regular in shape and more strongly cutinised than other epidermal cells. Each cell bear a solid papilla, overarching stomata aperture, outer margin of the cell is thickened and elevated above epidermal level (Supp. Fig. 27:12). Sometimes stomata are adjacent but do not share subsidiary cells. Encircling cells are present, but usually they are not forming a complete circle.

Only one specimen, found only in bromalite 1.

Discussion. Described leaf fragment is 1,8 mm long and 1,7 mm wide. It can originate from elongated entire leaf or from a pinnate compound leaf. It is hard to distinguish costal/intercostal zones, or any feature that could indicate possible venation. The density of stomata seems to be higher closer to the narrowing part of the cuticle. The general outline of the cuticle features shows some similarities to Coniferales, but the incomplete preservation and so far lack of similar cuticles identified from the macrofossils of that locality makes diagnosis unreliable.

Material: bromalite 1 specimen: 22

Cuticle type E (Supp. Fig. 27:13-15)

Description. This morphotype describes cuticles of incomplete leaves and cuticle fragments. Leaves were elongated, linear-lanceolate. The most complete leaf has a partially preserved leaf tip that could have been acute to obtuse, when complete (Supp. Fig. 27:13). The leaf base is not

preserved in any specimens. The most complete leaves are: 4,3 mm long/2,3 mm wide and 3,6 mm long/1,4 mm wide. The biggest cuticle is 4,4 mm long and 3,3 mm wide. Margins of leaf is preserved, with occasional minute teeth or marginal cells forming a scarious edge (Supp. Fig. 27:14). Uniformly developed cuticle is moderately thick with strongly cutinised cell walls, sometimes granular ornamentation is present.

Epidermal cells are mostly quadratic and isodiametric, with slightly rounded corners, arranged in loose rows. Epidermal cells in rows alternating stomata rows are slightly more elongated. Anticlinal walls are straight, evenly cutinised, sometimes with bulging projections. Periclinal walls bear a solid papilla in the center of a cell. Most of the papillae are prominent, some are more round and short, some longer and pointed, even hair-like. Longer papillae show striae from tip of the papillae to its base. Usually, 4-7 epidermal cells rows separate stomata rows.

Stomata are distributed irregularly in rows, single stomata wide. Sometimes stomata complexes are adjacent, but never share a subsidiary cell. Irregularly orientated guard cells are thinly cutinised and sunken, surrounded by a ring of 4 - 6 subsidiary cells, each bearing a papilla pointing towards center of a pit (Supp. Fig. 27:15), but rarely overarching it completely. Encircling cells are present in some specimens, usually forming an incomplete ring. Sometimes incomplete second ring of encircling cells is visible.

Type present in all three bromalites.

Discussion. The shape of the most complete cuticles indicate that the leaves were elongated, presumably needle-like shape. Unfortunately, the lack of leaf base precludes reliable leaf shape reconstruction. Additionally, leaves are broadening slightly from the apex. Also, the cuticle pattern is not differentiating towards the leaf base, whereas it is usually at least slightly different closer to the leaf end. Therefore, the cuticle might have been part of a much longer leaf blade. The biggest fragmented cuticle assigned to this type is indicating that the leaves could be much bigger than the ones already found. Cuticle of this type show resemblance to needle-like Coniferales macrofossils, found previously in the Lipie Śląskie sediments (Wawrzyniak 2010a-d), but the cuticle could not be obtained.

Material:
Bromalite 1: 40 (fragment), 41 (fragment, broken into 2 pieces), 64 (leaf), 67 (fragments, leaf torn apart, tip incomplete but could be rounded, acute, upper and lower cuticle, one cuticle slightly thicker), 72 (lower and upper cuticle, one cuticle slightly ticker, no distinctive difference between number of stomata, but that is caused by incomplete and unevenly preserved surface of both cuticles), 75 (fragment), 76 (fragment)
Bromalite 2: 21 (fragment), 22 (fragment, conspicuous stomata rows)
Bromalite 3: 34 (leaf no tip, no base)

Cuticle type F (Supp. Fig. 28:1-3)
Description. In this type gather are only cuticle fragments, none of them have preserved stomata. The biggest fragment is 3,1 mm long and 2,8 mm wide. Epidermal cells are polygonal vaguely differentiated into costal and intercostal fields (Supp. Fig. 28:1,2). Cells in the costal fields are elongated and aligned in longitudinal rows, cells in the intercostal fields are shorter to isodiametric and arranged more irregularly. Corners of epidermal cells are slightly rounded. Anticlinal walls are straight or gently sinuous (undulated) (Supp. Fig. 28: 3). Prominent papillae are developed on periclinal walls only of shorter cells or are present on every epidermal cell; longer cells show a darkening in the middle where the cuticle was probably thicker.

Type present in bromalite 1 and 2.

Discussion. Stomata are the most important feature in the taxonomical identification of cuticles. Hypostomatic (stomata only on abaxial leaf side) and epistomatic (stomata only on adaxial leaf side) plant taxa is not a feature characterizing any particular group among gymnosperms. In addition, in amphistomatic plants number of stomata on upper and lower cuticle can differ. Usually there is a greater number of stomata on the abaxial leaf side, therefore, it cannot be excluded that the cuticles are coming from the adaxial leaf side, because of their fragmentary preservation, and it cannot be define if the plant was amphi-, hypo-, or epistomatous. The curved alignment of cell rows visible in some specimens might indicate that the cuticle could be coming from other parts of the plant, i.e. cone, seed, stem, and not necessarily from the leaf.

Material:

Bromalite 1: 05 (fragment), 68 (fragment), 73 (fragment)

Bromalite 2: 05 (fragment), 06 (fragment), 8 (fragment, no stomata, papillae look like getting bigger and more dense on one side of the cuticle)

Cuticle type G (Supp. Fig. 28:4-6)

Description. Fragments of moderately thick cuticle. The biggest cuticle is 4,3 long and 3,3 wide. Epidermal cells arranged in longitudinal rows are polygonal, sometimes rectangular to isodiametric (Supp. Fig. 28:4). Anticlinal walls are thick and straight, evenly cutinised. Periclinal walls are bearing a central, hollow papillae (Supp. Fig. 28:5), or papillae are absent.

Stomata are distributed in loose rows or scattered. The size of stomata complex differs from 40 to 65 mm diameter across. Guard cells are deeply sunken, orientated irregularly. There are usually 5 subsidiary cells bearing a solid papillae, directed inwards the stomata pit, often overarching the aperture. Anticlinal walls of subsidiary cells are strongly cutinised (Supp. Fig. 28: 6). Encircling cells are unspecialised.

Type present in bromalite 1 and 3.

Discussion. All cuticles are similar in the general pattern of epidermis and type of stomata complex. They show resemblance to conifer cuticles, but their affinity cannot be determined.

Material:

Bromalite 1: 25 (fragment, no papillae), 31 (fragment, part of margin preserved)

Bromalite 3: 09 (fragment), 11 (fragment), 32 (fragment)

Cuticle type H (Supp. Fig. 28:7-9)

Description. Fragments of thick cuticle. Generally uniformed cuticles are similar in size, with length equal 2,4 mm and width 2,3 mm. Cells are arranged in longitudinal rows, stomata rows are intercalated with epidermal cells rows. On one cuticle discernible is a band of slightly elongated, polygonal epidermal cells arranged in ca. 14 rows without stomata (Supp. Fig. 28:7). Polygonal epidermal cells are isodiametric, but single row of elongated, narrow cells is separating stomata rows. Anticlinal walls of epidermal cells are thick and straight, mostly evenly cutinised, sometimes small bulging is present. Periclinal walls show a delicate granulation, papillae are rare and faint, if present they are placed closer to the one side of a cell. Resin bodies are absent, but masses of resin can be spotted between epidermal cells.

Stomata are arranged irregularly in longitudinal rows (Supp. Fig. 28:8). In stomata complex sunken guard cells are usually surrounded by 5 to 6 subsidiary cells. Subsidiary cells do not show stronger cutinisation than epidermal cells, except for their inner margin that usually is ended with flat papillae pointing towards center of the stomata pit (Supp. Fig. 28:9). Subsidiary cells may vary

in shape and size within single stomata. Stomata are orientated irregularly. Encircling cells are not specialized.

Type present in bromalite 2 and 3.

Diagnosis. Only one side of the cuticle is present. No apices, bases or margins are preserved. The intercostal field without stomata might mark the area of cuticle over venation, otherwise cuticles have rather uniformed outline. It indicates that cuticles must be a part of a much bigger leaf blade. The general structure of cuticles show resemblance to conifer type cuticles, but because of lack of natural margins enabling the reconstruction of the natural leaf shape the reliable diagnosis is not possible. This is also the first time this type of cuticle has been recognized in the Lipie Śląskie sediment.

Material:

Bromalite 2: 07 (fragment)

Bromalite 3: 36 (fragment)

Cuticle type I (Supp. Fig. 28:10-12)

Description. Fragments of moderately thick cuticle. Epidermal cells are arranged in longitudinal rows. No venation could be distinguished from cell arrangement. Epidermal cells are rectangular and elongated, more elongated between stomata rows, some twice as wide (Supp. Fig. 28:10). Anticlinal walls are thick and irregularly cutinised with pitting, bulging and granulation (Supp. Fig. 28:12). Periclinal walls are generally smooth, but shorter cells show a faint hollow papillae and sometimes granulation occurs.

Stomata are arranged in vague rows, irregularly distributed within them. Stomata complexes have elliptical shape. Thinly cutinised guard cells are sunken, orientated transversely to obliquely. Guard cells are surrounded usually by 4-5 subsidiary cells. Subsidiary cells are more cutinised than other epidermal cells, especially their inner margin (Supp. Fig. 28:11,12). Anticlinal walls between subsidiary cells are thinner and sometimes hard to distinguish. There are no papillae on subsidiary cells. Sometimes striae is visible overspreading from the center of the pit towards the outer edge of a cell. Adjacent stomata never share a subsidiary cell. Encircling cells are not specialized or form an incomplete circle, but cells are never more cutinised than ordinary epidermal cells.

Type present in bromalites 1 and 3.

Discussion. Two cuticles are similar in general cuticle arrangement. They might originate from the same plant or plant family. Unfortunately, fragmentary character and lack of natural margins that would allow the reconstruction of the original leaf shape unable the reliable diagnosis. Tentatively, cuticles are assigned to Coniferales, as they show features described before by Harris (1979), Stockey (1994).

Material:

Bromalite 1: 39 (fragment)

Bromalite 3: 15 (fragment)

Cuticle type J (Supp. Fig. 29:1-3)

Description. Cuticle fragments and nearly complete cuticles. All cuticles are non-cellular. Shape of the most complete specimens and the characteristic structure indicates that this morphotype might in fact represent cutinised megaspore membrane (Supp. Fig. 29:1). Cuticle surface is sculptured, sometimes with discernible reticulate pattern (Supp. Fig. 29:2). The presence of a thin membrane on one cuticle suggests that this might be (fused?) megaspore membrane with the

nucellar cuticle (Supp. Fig. 29:3). On the thin nucellar cuticle visible are outlines of elongated tetragonal cells with straight anticlinal walls, arranged in longitudinal rows.

Discussion. Because cuticles are isolated and incomplete exact affinity is hard to define, and this type can be assigned to different gymnosperm lineages. Similar structures were also recognized in the carnivorous bromalites from Lipie Śląskie (Zatoń et al., 2015).

Type present in bromalite 1 and 3.

Material:

Bromalite 1: 06 (fragment, fragments of both cuticle, ellipsoidal shape), 17 (fragment), 60 (fragment), 61 (nearly complete cuticle), 66 (nearly complete cuticle)

Bromalite 3:18 (fragment, cuticle with thin membrane), 30 (nearly complete cuticles), 33 (fragment)

Cuticle type K (Supp. Fig. 29:4-6)

Description. Almost complete cuticle and cuticle fragments. The cuticle has an ellipsoidal shape, slightly pointed on one side and more round on the other. Cuticle is thick and robust. Margins of the cuticle are smooth and entire. There are characteristic dark strips formed by sclerenchymatous fibers on the underside lengthwise of the cuticle Supp. Fig. 29:4).

Both cuticles have similar character. Epidermal cells are rectangular, isodiametric with corners rounded, arranged in longitudinal rows. Cells in rows with stomata are bigger and more quadratic than cells in alternate rows (Supp. Fig. 29:5). Anticlinal walls are very thick. Periclinal walls sometimes show granular ornamentation and small papillae.

Stomata are arranged in rows. Guard cells are deeply sunken surrounded by 6-7 subsidiary cells forming a round stomata complex (Supp. Fig. 29:6). Subsidiary cells usually have the same size and shape. They are strongly cutinised with some solid papillae on every cell, directed towards the pit. Encircling cells are unspecialised.

Present in bromalite 1 and 3.

Discussion. Epidermal features including shape of the stomata show resemblance to Coniferales. The shape and general layout of the cuticle suggest it might come from the pollen cone scale.

Material:

Bromalite 1: 56 (almost complete specimen, two attached cuticles), 63 (fragment), 70 (fragment)

Bromalite 3: 31 (fragment)

Palynomorphs

Palynomorphs were found in one of the analyzed samples of the plant-rich bromalites (Bajdek et al., 2014). The identified palynotaxa include *Corollina meyeriana*, *Corollina* sp., *Monosulcites minimus* and *Porcellispora* sp. They could have been ingested by the faeces producer with forage (host plant), or digested accidentally, for example with drinking water. If they represent the consumed plant species, it indicates that the diet of this dicynodont species included gymnosperm plants. Remains of fungi were not found in the material.

Additional comments

Preliminary inferences for the physiology of the Late Triassic dicynodont from the Lisowice locality, in part based on the bromalite material, were presented by Bajdek et al. (2014) who concluded that dicynodonts seem to have had a relatively slow metabolism (i.e. long food retention time) and to have consumed relatively small amounts of forage, based on the facts that (a) the Lisowice dicynodont was toothless and (b) its feces samples reveal a high quality diet (usually no

wood fragments) and (c) except some very few specimens with a woody content, the plant elements have been well decomposed. Moreover, Bajdek et al. (2014) noted the possible metabolic advantage of the gigantism of Lisowice dicynodont, since the capacity of food retention depends on the gut volume. Notwithstanding, Bajdek et al. (2014) presented also a spectrum of preservation state and diagenetic alteration of the bromalite specimens; a rarity of plant remains and organic matter in some of the specimens results from a high degree of pyritization.

3.4.3. Plant remains from *Smok wawelski* bromalites

Ten, medium to large, non-spiral and phosphatic bromalites (12 to 25 cm long) collected from Lisowice (Supp. Fig. 19f-n) are matched, by their dimensions and by association with body fossils and footprints, to *Smok wawelski* (Supp. Fig. 10e). The bromalites contain fragments of plants, mineral grains, large serrated teeth as well as up to 50% of bone fragments (Qvarnström et al., 2019a), with distinct fragmentation and angularity, from several prey taxa. This suggests pronounced osteophagy of their producers (Niedźwiedzki, 2013). Further evidence for bone-crushing behaviour is provided by isolated worn teeth, bone-rich regurgitalites (fossil vomits) and numerous examples of crushed or bite-marked dicynodont bones, all collected from the same bone-bearing beds. Several of the anatomical characters related to osteophagy (Niedźwiedzki et al., 2011; Niedźwiedzki, 2013; Qvarnström et al., 2019a).

The picture of floral assemblage from Lisowice can be supplemented by the data from *Smok wawelski* faeces. Previously investigated carnivorous bromalites from Lisowice reviled some plant cuticles and tracheids. Cuticles are shredded, but otherwise they are not much degraded and some morphological features can be observed. A few cuticles show similarity to *Lepidopteris ottonis* and *Podozamites* leaves (Zatoń et al., 2015).

In total, 4 organic-rich bromalite fragments from specimens ZPAL V.33/340 and 342 (labelled A-D; see Supp. Table 6) were selected from the bromalite collection and dissolved in a mixture of buffered formic acid (3–5%) and acetic acid (2–3%). The solutions with immersed bromalites were heated several times to around 50 °C to accelerate dissolution (approximately 2 h total). The process lasted 2–3 weeks until the mineralized bromalite mass was completely disintegrated. The samples were then washed with distilled water to carry the mineral suspension away. Finally, all larger fragments of plant cuticles observed in undissolved residue were removed and preserved in plastic tubes for future studies.

Plant cuticles were not abundant in the examined material. About 20 slides contain about 120 pieces of cuticles. Some of them represent a large part of leaves, fragments with apices are relatively common. All preserved cuticles are rather thick. Six types of cuticles were distinguished (Supp. Fig. 30). Most of the material belongs to conifer of cheirolepidaceous appearance. According to the leaf shapes, they represent *Brachyphyllum* sp. (Type 1), *Pagiophyllum* sp. 1 and *Pagiophyllum* sp. 2 (Types 2 and 3), undetermined Coniferales gen. et sp. (Type 4), cf. *Nilssonia* sp. (Type 5) and indeterminable cuticle probably of cycadalean affinity (Type 6). In the bromalite of *Smok wawelski*, was found almost complete small leaves that undoubtedly belong to *Brachyphyllum*-type of shoots.

3.5. Bromalites from Marciszów

The material comprises 5 bromalite specimens, which represent herbivore droppings (morphotypes M1-M2). Detailed specimen list and descriptions are provided in the Supp. Table 6. The specimens were collected during excavations at the Marciszow site organized during 2018–2019. All specimens are housed in the Institute of Paleobiology, Poland, and catalogued under registration number ZPAL Mar 1-5 (Supp. Table 6). Specimens are more or less irregular, oval-shaped and comprise small mineral particles. Some are in places highly mineralized by pyrite and contain highly fragmented and macroscopically visible plant material. Preliminary study of two specimens shows the presence of plant tissues and amorphous and dark organic material. All studied specimens were found in organic-rich, mudstone/conglomerate intercalations. The studies specimens are comparable in general appearance (grey, oval, with calcite veins) and chemical composition (calcareous and carbon-rich) to putative dicynodont bromalites described from the Lisowice site (see Bajdek et al., 2014). The results of the examination (SEM, EDS, MTS, ME, and results from dissolution) of all collected specimens are summarized in Supp. Table 6.

3.6. Bromalites from Gromadzice-Rzuchów

A total of 12 bromalites were collected from the section exposed at Gromadzice- Rzuchów. All specimens are housed in the Polish Geological Institute-National Research Institute, Poland, and catalogued under registration number Muz. PGI OS GR/Cop 1-12 (Supp. Table 7). Major part of these bromalites are poorly preserved, elongated, phosphatic or sideritic specimens and cannot be accurately assigned to specific theropod producers (morphotype M3-M4). These bromalites contain fragmnents of bones or fish scales, so they come from predators, and the fact that they co-occur with numerous theropod tracks allows for their general assignment. The diagnosable specimens (5 bromalites) have all been identified as bromalites of large herbivores, most likely sauropodomorphs (morphotype M1-M2), based on a suite of morphological features (oval-shaped or irregular) that includes their overall external morphology and the content of numerous plants remains. These bromalites were compared to other Late Triassic plant-rich specimens documented in Bajdek et al. (2014) as well as bromalite material from the Lower Jurassic of Sołtyków (Barbacka et al., 2022) and some specimens described in the present study. The results of the examination (SEM, EDS, MTS, ME, and results from dissolution) of all collected specimens are summarized in Supp. Table 7.

3.7. Bromalites from Hucisko

Among 16 bromalites obtained from the investigated locality (Supp. Table 8), 5 representative specimens were selected for more detailed investigation. All collected specimens are housed in the Polish Geological Institute-National Research Institute, Poland, and catalogued under registration number Muz PGI OS HU/Cop 1-16. The smallest specimen is 2.3 cm long anteroposteriorly, while the largest ones are in the range of 14 cm in length and 5 cm in their greatest width. All specimens are non-spiral, and two morphotypes (M1-M2) are recognized based on the external morphology of the bromalites. Both the shape and elemental geochemistry of these bromalites (they are phosphate-based) indicate that they are the feces of predators, most likely theropod dinosaurs. The results of the examination (SEM, EDS, MTS, ME, and results from dissolution) of all collected specimens are summarized in Supp. Table 8.

3.8. Bromalites from Sołtyków

The record of bromalites from Sołtyków is unique in terms of the number of specimens collected (over 300) and also their association with an early Hettangian dinosaur-dominated assemblage. In total, 148 registered and over 200 unregistered bromalites have been collected from Sołtyków (during 1996 and 1997) but many specimens are secondarily mineralised by siderite making their contents difficult to study (Supp. Table 9). The issue of sideritization of bromalites and organic matter originating from the digestive tract of vertebrates has been described by Seilacher et al. (2001) and Brachaniec et al. (2022).

All specimens were collected *in situ* from four bromalite-bearing intervals, in several exposed sections, which were well exposed at the Sołtyków clay-pit from 1996 to 1997 (Supp. Fig. 9). All bromalites were catalogued (Muz. PGI OS SO/Cop 1-148) and photographed (Supp. Fig. 20-26). The bromalites were divided into several groups (M1-M11) based on size, composition, and morphology (Supp. Table 9). The smallest specimen is 0.7 cm long anteroposteriorly, while the largest ones are in the range of 34 cm in length and 11 cm in their greatest width. Some specimens that were considered promising were selected for additional studies (thin sections, CT scans, and acid dissolution). Bromalites from Sołtyków have not been synchrotron scanned and preliminary CT scans of two complete bromalites (Muz. PGI OS-221/302 and 306) were not successful as the contrast between inclusions and the highly mineralised bromalite groundmass was not sufficient. Their contents are known from visual inspection, mechanical/chemical preparation of the bromalites, and analysis of the material from dissolved specimens. Completely siderised small bromalites (diameter less than a centimetre) are known from organic-rich lacustrine intervals of the section. These are elongated but of different morphology and length. Some have a somewhat curved or even spiral structure although many morphological features are obscured by the secondary mineralisation. The likely bromalite makers are actinopterygian, and perhaps sarcopterygian, fish of different sizes, which suggests that the diversity of fishes was higher than known from the scarce body fossil record.

The biggest bromalites from Sołtyków are well above 30 centimetres in length (Supp. Fig. 20). Some of these were evidently produced by carnivores as crocodylomorph bones as well fragments of larger bones have been observed as inclusions (Supp. Fig. 32). The likely producers of these bromalites are big theropods which are known from over 55-cm long tridactyl footprints (ichnogenus *Kayentapus* and *Megalosauripus*). Moreover, fish remains occur in somewhat smaller bromalites suggesting that smaller theropods were at least in part piscivorous.

The results of the examination (SEM, EDS, MTS, ME, and results from dissolution) of all collected specimens are summarized in Supp. Table 9.

3.8.1. Plant remains extracted from bromalites

Bromalites of five different morphotypes from Sołtyków contain plant remains (Barbacka et al., 2022). These derive from specimens produced by both herbivores and carnivores. They even occur in large bone-bearing bromalites. Plant remains in carnivore bromalites are commonly better-preserved, suggesting that involuntarily-ingested plant remains were not efficiently digested by carnivores. The rich material of plant cuticles was extracted from seven bromalite samples. In total 14 types of leaf cuticles were recognised, of which some examples are shown in supplementary Supp. Fig. 31. The cuticles show different degree of fragmentation, from distinctive leaf fragments, to very fine undefined debris. The exceptional value of the record is that in eight identified types of cuticle/taxa of total ten leaf fragments with both, upper and lower cuticles are preserved, and they often are characteristic enough for at least generic identification. The diversity of the cuticles from these organic-rich bromalites is different from other floral assemblages from the Holy Cross

Mts. The reason for that is that the macroflora has only sporadically been collected within limited territories, either associated with mining activities in alluvial deposits (Sołtyków) or with drilling (cores). Although we have information about numerous gymnosperm plants in the Lower Jurassic deposits of the Holy Cross Mts., our knowledge about the whole flora is very incomplete. Only one cuticle extracted from the bromalites resembles the cuticle of plant species previously found in Sołtyków (cf. *Podozamites* sp.). Some cuticles belong to taxa which are common in the territory of the northern margin of the Holy Cross Mts. (*Nilssonia*, *Pterophyllum*, *Pseudotorellia*, *Pachypteris, Desmiophyllum harrisii*, Ginkgophyta, Coniferophyta), but they represented different species. Some provide new data of their occurrence in Poland and belong to a new species (*Komlopteris distinctiva*, *Aciphyllum triangulatum*).

Palynological studies were carried out on samples and plant cuticle preparations obtained from six bromalites of herbivorous dinosaurs and on plant cuticle preparations from six bromalites of predatory dinosaurs. Thirty-one taxa of spores and pollen grains were found in samples from bromalites of herbivorous dinosaurs and fourteen taxa from bromalites of predatory dinosaurs, of which four taxa were not present in samples from bromalites of herbivores. The spores and pollen grains belonged to various groups of plants such as bryophytes, clubmosses, horsetails, ferns, seed ferns, bennettites or cycads, ginkgoaleans and conifers. The most numerous samples from dinosaur bromalites were fern spores, microspores of *Aratrisporites minimus* from the clubmoss plant and pollen grains of *Classopollis torosus* from the coniferous plant. Previous studies of the sporomorph assemblage from the outcrop sediment in Sołtyków showed the occurrence of sixty-three taxa (Ziaja, 2006). Of the 35 spore and pollen grain taxa determined from dinosaur bromalite samples, most were also present in sediment samples. The exception are six taxa: *Stereisporites steroides, Stereisporites antiquasporites, Dictyophyllidites harrisii,* cf. *Gleicheniidites umbonatus, Riccisporites tuberculatus* and cf. *Eucommiidites* sp., which were determined only from bromalite samples. The presence of *Aratrisporites minimus* microspores and *Nathorstisporites hopliticus* megapores in the examined samples confirms that the dinosaur bromalites from Sołtyków come from the Hettangian, which is consistent with the age of the outcrop sediment (Pieńkowski, 2004a, b; Ziaja, 2006). The occurrence of sporomorphs in dinosaur bromalites does not necessarily mean that the animals ate the plants that produced these sporomorphs. Spores and pollen grains may have been on the surfaces of other plants or animals eaten by dinosaurs, as well as in the water they drank.

3.9. Bromalite geochemistry

Molecular studies of Mesozoic vertebrate bromalites are rare (see Zatoń et al. 2015). Bromalites from the Late Triassic of Polish Basin have already been the subject of geochemical research. The first study of this type was presented by Zatoń et al. (2015) who obtained the presence of specific biomarkers such as sitosterol and amyrins typical for higher plants or phytanic and pristanic acids, which are characteristic constituents of fish oil in selected bromalites from Poręba and Lisowice sites. Triassic bromalites also showed the presence of other labile organic compounds as sterols, palmitin, stearin or levoglucosan attests for rapid, microbially-mediated mineralization of the faeces at very early stages of diagenesis.

As part of the project, a number of geochemical studies were carried out on bromalites from the Late Triassic and Early Jurassic. The most interesting results for paleoecological considerations are presented in this study. The results of the geochemical examination are summarized in Supp. Tabs. 5, 6 and 9.

3.9.1. Sołtyków bromalites

3.9.1.1. Bulk data and petrographic observations

Most bromalite samples contain moderate to high contents of total organic carbon (TOC), ranging from 0.5 to 42% wt. Only two carbonate samples were organic-poor (Muz PGI OS-221/384 = SOL_9a and SOL_9b), with TOC contents not exceeding 0.1% wt. (Supp. Table 10), likely caused by oxidation (weathering) of the samples, which is also manifested by the presence of macroscopically visible hematite. Total sulphur (TS) content for all bromalites, but one is low or very low (0.1-0.5% wt.) and does not correlate with TOC. In one sample (Muz PGI OS-221/383 = SOL_8a), high TS content is connected with pyrite incrustation (Supp. Table 10).

Extractable organic compounds are present in all samples, apart from two organic-poor bromalites. The distribution of *n*-alkanes is diverse, showing a predominance of short-chain (samples Muz PGI OS-221/376 = SOL_1b and c; Muz PGI OS-221/381 = SOL_6; Muz PGI OS-221/383 = SOL_8a, and b) or long-chain *n*-alkanes (all remaining samples – Supp. Table 11). All samples show a majority of odd-carbon, high-molecular-weight *n*-alkanes, which is exemplified by higher than one values of $CPI_{(25-31)}$ parameter (Supp. Table 11). It is best seen with SOL_1a, b, c (Muz PGI OS-221/376), SOL_3 (Muz PGI OS-221/378), SOL_5 (Muz PGI OS-221/380) and SOL_7 (Muz PGI OS-221/382) samples (values > 1.3; Supp. Fig. 33). Values of pristane to phytane (Pr/Ph) ratio and isoprenoids to *n*-alkanes ratios are highly diverse and non-indicative (Supp. Table 11).

Petrographic observation shows that the main maceral present in bromalites is inertinite (pyrofusinite). These occur as quite large fragments (Supp. Fig. 34) or, more commonly, small crushed, sometimes partially mineralized components (Supp. Fig. 34B). Inertinites are usually characterized by well-preserved wood structures (Supp. Fig. 34A), and radial sections are more common than tangential with characteristic fibrous shapes and pitting within preserved tracheids (Supp. Fig. 34D). Vitrinites were also found in bromalite samples, but they are much rarer.

3.9.1.2. Polycyclic aromatic hydrocarbons (PAHs)

In the aromatic fraction of bromalite extract, dominant compounds are PAHs with distribution typical for pyrolytic processes (Supp. Table 12; Supp. Fig. 35). Unsubstituted PAHs significantly dominates over their methylated derivatives, and five-ring PAHs are one of the most abundant compounds (Supp. Fig. 35). Concentrations of PAHs in bromalites are similar to sedimentary rocks from the Sołtyków site (Marynowski and Simoneit, 2009) and very high compared to other sections where wildfires were also detected (Marynowski and Simoneit, 2009; Marynowski et al., 2011). The highest PAHs concentration was found for the SOL_1a, b, c (Muz PGI OS-221/376), SOL_5 Muz PGI OS-221/380), and SOL_7 (Muz PGI OS-221/382) samples, the same which contain the highest $CPI_{(25-31)}$ values (Supp. Table 12). Perylene, five-ring PAH present in most of the samples, is of non-pyrolytic origin, connected with the fungal activity (Grice et al., 2009).

3.9.1.3. Biomarkers of terrestrial origin

Many terrestrial biomarkers were identified. They are mainly aromatic compounds, including dehydroabietane, 16,17-bisnordehydroabietane, 18-norabieta-6,8,11, 13-tetraene, simonellite, C1 simonellite, diaromatic totarane, 6-isopropyl-2-methyl-1-(4-methylpentyl) naphthalene, 1,2,3,4-tetrahydroretene, retene and 2-methylretene (Supp. Fig. 36). All these compounds are characteristic as primary constituents of conifers (e.g. dehydroabietane) or diagenetic products of diterpenoids from conifer resin (e.g., Ellis et al., 1996; Otto and Simoneit, 2001; Otto and Wilde, 2001; Otto et al., 2007; Paul and Dutta, 2020). Moreover, ferruginol, totarol, and sugiol were found

in the polar fraction of Muz PGI OS-221/376 = SOL_1a-c and Muz PGI OS-221/378 = SOL_3 bromalites.

Other identified polar compounds of terrestrial origin are phenolic derivatives, typical lignin degradation products (e.g., Rybicki et al., 2017). Such compounds as 4-hydroxybenzaldehyde, resorcinol, 4'-hydroxyacetophenone, vanillin, 4-hydroxybenzoic acid, phthalic acid, vanillic acid and homovanillic acid were identified in most of the bromalite studied.

3.9.2. Discussion

Classes of compounds from two different origins were identified in the bromalites from Sołtyków. The first is pyrolytic PAHs, which together with charcoal (inertinite) fragments collectively evidence that burnt plant remains were part of the dinosaurs' diet. In another study, Zatoń et al. (2015) identified levoglucosan in bromalites from Upper Triassic deposits. This compound is specific for biomass burning (Simoneit et al., 1999). Here, many crushed inertinites in all sampled bromalites suggest that charcoal fragments were excreted through the gastrointestinal tract and not mechanically incorporated into the faecal mass after excretion. The fact that many of the plants eaten by the dinosaurs were charred can be explained by the widespread wildfires on the T/J boundary and during the lowermost Jurassic (Marynowski and Simoneit, 2009; Belcher et al., 2010; Petersen and Lindström, 2012; Lindström et al., 2021). It is also possible that dinosaurs intentionally consumed charred plants to clean their gut and detoxify to prevent sickness (Buck and Bratich, 1986; Poage et al., 2000). Activated charcoal effectively treats toxicosis induced by mycotoxins, glycosides, plant alkaloids, and is useful in binding other phytotoxins (Buck and Bratich, 1986).

The second group of compounds is terrestrial organic compounds, including gymnosperm biomarkers, from the primary unburned organic matter. Belz et al. (2020) defined, based on former literature, ternary diagrams of vegetation reconstruction of southern African plants (Supp. Fig. 37 - simplified). Depending on using *n*-alkanes or *n*-alkan-1-ols bromalite samples are located in rain forest area or savannah area (Supp. Fig. 37). Such inconsistency would be connected with diagenetic changes of less stable *n*-alkan-1-ols and preferential degradation of *n*-C_{30} and *n*-C_{32} alkan-1-ols in geological materials and different types of contemporary and Jurassic vegetation. Moreover, part of *n*-alkan-1-ols can originate from burned organic matter (Simoneit, 2002). It seems that *n*-alkanes distribution better reflects the actual sources of the dinosaurs' diet (Supp. Fig. 37), placing it in the rain forest field.

Aromatic diterpenoids were identified as the dominant compounds in some bromalite samples (Muz PGI OS-221/376 = SOL_1a-c; Muz PGI OS-221/378 = SOL_3; Muz PGI OS-221/382 = SOL_7; Supp. Fig. 37). These are conifer biomarkers representative for Cupressaceae, Araucariaceae and Podocarpaceae families. The other characteristic group of biomarkers detected in bromalites are hopanes, compounds diagnostic for bacteria (Peters et al., 2005). The occurrence of these compounds is possibly connected with the bacterial reworking of bromalites before and during mineralization (see Zatoń et al., 2005). Fungal activity is emphasized by the occurrence of perylene; a compound originated from ectomycorrhizal and wood-rot fungi (Grice et al., 2009; Itoh et al., 2012; Marynowski et al., 2013).

Additional References

Achilles, H., 1981. Die rhaetische und liassische Mikroflora Frankens. *Palaeontographica Abteilung B Paläophytologie* **179**, 1–86.

Alvin, K.L., 1982. Cheirolepidiaceae: biology, structure and palaeoecology. *Review*

Palaeobotany Palynology **37**, 71–98.

Antczak, M. Bodzioch, A., 2018. Diversity of fish scales in Late Triassic deposits of Krasiejów (SW Poland). *Paleontological Research* **22**, 91–100.

Argyriou, T., et al. 2016. Exceptional preservation reveals gastrointestinal anatomy and evolution in early actinopterygian fishes. *Scientific Reports* **6**, 18758. https://doi.org/10.1038/srep18758.

Ash, S.R., 1999. An Upper Triassic upland flora from north-central New Mexico, U.S.A. *Review of Palaeobotany and Palynology* **105**, 183–199.

Bajdek, P., et al. 2016. Microbiota and food residues including possible evidence of pre-mammalian hair in Upper Permian coprolites from Russia. *Lethaia* **49**, 455–477. https://doi.org/10.1111/let.12156

Bajdek, P., et al. 2016. Residues from the Upper Permian carnivore coprolites from Vyazniki in Russia - key questions in reconstruction of feeding habits. *Palaeogeography, Palaeoclimatology, Palaeoecology* **482**, 70–82.

Bajdek, P., et al. 2019. Bromalites from a turtle-dominated fossil assemblage from the Triassic of Poland. *Palaeogeography, Palaeoclimatology, Palaeoecology* **520**, 214–228. https://doi.org/10.1016/j.palaeo.2019.02.002

Bajdek, P., et al. 2014. Putative dicynodont coprolites from the Upper Triassic of Poland. *Palaeogeography, Palaeoclimatology, Palaeoecology* **411**, 1–17. http://dx.doi.org/10.1016/j.palaeo.2014.06.013

Barbacka, M., et al. 2007. *Hirmeriella muensteri* (Schenk) Jung from Odrowąż (Poland), with female and male cones, and in situ *Classopolis* pollen grains. *Acta Palaeobotanica* **47**, 339–357.

Barbacka, M., et al. 2010. Taxonomy and palaeoecology of the Early Jurassic macroflora from Odrowąż, central Poland. *Acta Geologica Polonica* **60**, 373–392.

Barbacka, M., et al. 2016. New data about *Matonia braunii* (Göppert) Harris from the Early Jurassic of Poland and its ecology. *Geological Quarterly* **60**, 857–868.

Barbacka, M., et al. 2022. Early Jurassic coprolites: insights into palaeobotany and the feeding behaviour of dinosaurs. *Papers in Palaeontology* **8**, e1425. https://doi.org/10.1002/spp2.1425

Belcher, C.M., et al. 2010. Increased fire activity at the Triassic/Jurassic boundary in Greenland due to climate-driven floral change. *Nature Geoscience* **3**, 426–429.

Belz, L., et al. 2020. The leaf wax biomarker record of a Namibian salt pan reveals enhanced summer rainfall during the Last Glacial-Interglacial Transition. *Palaeogeography, Palaeoclimatology, Palaeoecology* **543**, 109561.

Bomfleur, B., et al. 2011. Fossil sites in the continental Victoria and Ferrar groups (Triassic–Jurassic) of North Victoria Land, Antarctica. Polarforschung **80**, 88–99.

Brachaniec, T., et al. 2022. Comparative actualistic study hints at origins of alleged Miocene coprolites of Poland. *PeerJ* 10, e13652 DOI 10.7717/peerj.13652

Brański, P., 2014. Climatic disaster at the Triassic–Jurassic boundary: a clay minerals and major elements record from the Polish Basin. *Geological Quarterly* **58**, 291–310.

Brusatte, S.L., et al. 2010. The origin and early radiation of dinosaurs. *Earth-Science Reviews* **101**, 68–100.

Brusatte, S.L., et al. 2008. The first 50Myr of dinosaur evolution: macroevolutionary pattern and morphological disparity. *Biology Letters* **4**, 733–736.

Brusatte, S.L., et al. 2011. Footprints pull origin and diversification of dinosaur stem lineage deep into Early Triassic. *Proceedings of the Royal Soceity B* **278**, 1107–1113.

Buck, W.B., Bratich, P.M., 1986. Activated charcoal: Preventing unnecessary death by poisoning. *Veterinary Medicine* **81**, 73–77.

Budziszewska-Karwowska, E., et al. 2010. Bite marks on an Upper Triassic dicynodontid tibia from Zawiercie, Kraków-Częstochowa Upland, southern Poland. *Palaios* **25**, 415–421.

Chin, K., 2002. Analyses of coprolites produced by carnivorous vertebrates. *The Paleontological Society Papers* **8**, 43–50.

Chin, K., 2007. The paleobiological implications of herbivorous dinosaur coprolites from the Upper Cretaceous Two Medicine Formation of Montana: Why Eat Wood? *Palaios* **22**, 554–566.

Clement-Westerhof, J.A., van Konijnenburg-van Cittert, J.H.A., 1991. *Hirmeriella muensteri*: New data on the fertile organs leading to a revised concept of the Cheiroleopiaeae. *Review of Palaeobotany and Palynology* **68**, 147–179.

Czepiński, Ł., et al. 2021. An Upper Triassic terrestrial vertebrate assemblage from the forgotten Kocury locality (Poland) with a new aetosaur taxon. *Journal of Vertebrate Paleontology* **41**, e1898977.

Dzik, J., Sulej, T., 2007. A review of the early Late Triassic Krasiejów biota from Silesia, Poland. *Palaeontologia Polonica* **64**, 3–27.

Dzik, J., et al. 2008a. Zaskakujące uwieńczenie ery gadów ssakokształtnych (A surprising culmination of the era of mammal-like reptiles). *Ewolucja* **3**, 2–21 (in Polish).

Dzik, J., et al. 2008b. A dicynodont-theropod association in the latest Triassic of Poland. *Acta Palaeontologica Polonica* **53**, 733–738.

Ellis, L., et al. 1996. Formation of isohexylalkylaromatic hydrocarbons from aromatization rearrangement of terpenoids in the sedimentary environment: A new class of biomarker. *Geochimica et Cosmochimica Acta* **60**, 4747–4763.

Fijałkowska, A., 1989. Badania sporowo-pyłkowe osadów dolnego liasu w profilu Skarżysko-Kamienna IG 1 (Spore and pollen studies of Lower Liassic sediments in the Skarżysko-Kamienna IG 1 profile). *Kwartalnik Geologiczny* **33**, 199–208 (in Polish, English summary).

Fijałkowska-Mader, A., 1999. Palynostratigraphy, palaeoecology and palaeoclimatology of the Triassic in South-Eastern Poland. *Epicontinental Triassic* **1**, 601–627.

Fijałkowska-Mader, A., 2015. A record of climatic changes in the Triassic palynological spectra from Poland. Geological Quarterly **59**, 615–653.

Fijałkowska-Mader, A., et al. 2015. Keuper palynostratigraphy and palynofacies of the Upper Silesia (southern Poland). *Annales Societatis Geologorum Poloniae* **85**, 637–661. 10.14241/asgp.2015.025

Franz, M., 2008. Litho- und Leitflächenstratigraphie, Chronostratigraphie, Zyklo- und Sequenzstratigraphie des Keupers im östlichen Zentraleuropäischen Becken (Deutschland, Polen) und Dänischen Becken (Dänemark, Schweden). 198 pp. Dissertation zur Erlangung des akademischen Grades doctor rerum naturalium (Dr. rer. nat.) vorgelegt der Naturwissenschaftlichen Fakultät III der Martin-Luther-Universität, Halle-Wittenberg.

Fuglewicz, R., Śnieżek, P., 1980. Megaspory górnego triasu z Lipia Śląskiego koło Lublińca (Upper Triassic megaspores from Lipie Śląskie near Lubliniec). *Przegląd Geologiczny* **28**, 459 (in Polish, English summary).

Fuglewicz, R., 1977. New species of megaspores from the Trias of Poland. *Acta Palaeontologica Polonica* **22**, 405–431.

Geyer, G., Kelber, K.P., 2018. Spinicaudata ("Conchostraca," Crustacea) from the Middle Keuper (Upper Triassic) of the southern Germanic Basin, with a review of Carnian–Norian taxa and suggested biozones. *Paläontologische Zeitschrift* **92**, 1–34.

Gierliński, G., et al. 2004. Tetrapod track assemblage in the Hettangian of Sołtyków, Poland, and its paleoenvironmental background. *Ichnos* **11**, 195–213.

Gordon, C.M., et al. 2020. Distinguishing regurgitalites and coprolites: a case study using a Triassic bromalite with soft tissue of the pseudosuchian archosaur *Revueltosaurus*. *Palaios* **35**, 111–121.

Gorzelak, P., et al. 2010. Pathologies of non-marine bivalve shells from the Late Triassic of Poland. *Lethaia* **43**, 285–289.

Grice, K., et al. 2009. New insights into the origin of perylene in geological samples. *Geochimica et Cosmochimica Acta* **73**, 6531–6543.

Harris, T.M., 1932. The Fossil Flora of Scoresby Sound East Greenland, Part 2: Description of seed plants incertae sedis together with a discussion of certain cycadophyte cuticules. *Meddelelser om Grønland* **85**, 1–112.

Harris, T.M., 1935. The fossil flora of Scoresby Sound, east Greenland. Part 4: Ginkgoales, Coniferales, Lycopodiales and isolated fructifications. *Meddelelser om Grønland* **112**, 1–176.

Harris, T.M., 1937. The fossil flora of Scoresby Sound East Greenland. Part 5. Stratigraphic relations. *Meddelelser om Grønland* **112**, 1–114.

Harris, T.M., 1979. The Yorkshire Jurassic Flora, V. Coniferales. British Museum (Natural History), London, 166 pp.

Heer, O., 1881. Contributions à la flore fossile du Portugal. *Comissão dos Trabalhos Geológicos de Portugal*, *Lisboa*, 51 pp.

Herngreen, G.F.W., 2005. Triassic sporomorphs of NW Europe: taxonomy, morphology and ranges of marker species with remarks on botanical relationship and ecology and comparison with ranges in the Alpine Triassic. *Nederlands Instituut voor Toegepaste Wetenschappen TNO*, *2005*, 1–83.

Huene, F., von., 1932. Die fossile Reptil-Ordnung Saurischia, ihre ntwicklung und Geschichte. *Monographien zur Geologie und Palaeontologie* **1**, 1–361.

Hunt, A.P., Lucas, S.G. 2012a. Classification of vertebrate coprolites and related trace fossils. In: Hunt, A.P., et al. (eds.), *Vertebrate Coprolites*. *New Mexico Museum of Natural History and Science Bulletin* **57**, 137–146.

Hunt, A.P., Lucas, S.G., 2012b. Descriptive terminology of coprolites and recent feces. In: Hunt, A.P., et al. (eds.), *Vertebrate Coprolites*. *New Mexico Museum of Natural History and Science Bulletin* **57**, 153–160.

Hunt, A.P., Lucas, S.G., 2014. Jurassic vertebrate bromalites of the western United States in the context of the global record. *Volumina Jurassica* **XII**, 151–158. http://dx.doi.org/10.5604/17313708.1130139

Cohen, K.M., Finney, S.C., Gibbard, P.L. & Fan, J.-X. (2013; updated). The ICS International Chronostratigraphic Chart. Episodes 36: 199-204.

Irmis, R.B., 2010. Evaluating hypotheses for the early diversification of dinosaurs. *Earth and Environmental Science Transactions of the Royal Society of Edinburgh* **101**, 397–426.

Itoh, N., et al. 2012. Perylene in lake Biwa sediments originating from *Cenococcum geophilum* in its catchment area. *Geochimica et Cosmochimica Acta* **95**, 241–251.

Jarzynka, A., Wawrzyniak, Z., 2012. Cuticle Analysis of the Late Triassic and Middle Jurassic Macroflora from Southern Poland as a Basis of Palaeoenvironmental Reconstructions. *SGC2012. 3rd Students International Geological Conference. April 27–30, 2012. Ivan Franko National University of Lviv. Lviv, Ukraine. Abstracts.* Lviv: Ivan Franko National University of Lviv, 88–89.

Jurkewiczowa, I., 1953. Wycieczka A. Jarugi-Gromadzice-Ostrowiec (Fieldtrip A. Jarugi-Gromadzice-Ostrowiec). In: *Porzewodnik Wycieczkowy Narady Państwowej Służby Geologicznej*, Warszawa, 28–34 (in Polish, English summary).

Jurkiewiczowa, I., 1967. Lias zachodniego obrzeżenia Gór Świętokrzyskich i jego paralelizacja z liasem Wyżyny Krakowsko-Częstochowskiej (The Liassic of the western part of the Mesozoic margin the Świętokrzyskie (Holy Cross) Mountains and its correlation with the Liassic of the Cracow-Częstochowa Area (. *Biuletyn Instytutu Geologicznego* **200**, 5–132 (in Polish, English summary).

Karasev, E.V., 2013. Formal system of dispersed leaf cuticles of Pteridosperms (Peltaspermaceae) from the Permian and Triassic of the Russian Platform. *Paleontological Journal* **47**, 335–349. 10.1134/S0031030113030052

Kendall, M.W., 1947. On five species of *Brachyphyllum* from the Jurassic of Yorkshire and Wiltshire. Annals and Magazine of Natural History, Series **11**, 225–251.

Kendall, M.W., 1948: On six species of *Pagiophyllum* from the Jurassic of Yorkshire and southern England. *Annals and Magazine of Natural History, London* **12**, 73–108.

Kerp, H., 1990. The study of fossil gymnosperms by means of cuticular analysis. *Palaios* **5**, 548–569.10.2307/3514861

Kopik, J., 1970. Retyk (Rhaetian). In: Pożaryski, W., (ed.), Stratygrafia mezozoiku obrzeżenia Gór Świętokrzyskich (The stratigraphy of the Mesozoic in the margin of the Świętokrzyskie Mts.). *Prace Instytutu Geologicznego* **56**, 7–46 (in Polish, English summary).

Kowal-Linka, M., et al. 2019. The youngest detrital zircons from the Upper Triassic Lipie Śląskie (Lisowice) continental deposits (Poland): Implications for the maximum depositional age of the Lisowice bone-bearing horizon. *Palaeogeography, Palaeoclimatology, Palaeoecology* **514**, 487–501.

Kowalski, J., et al. 2019. Preliminary report on the microvertebrate faunal remains from the Late Triassic locality at Krasiejów, SW Poland. *Annales Societatis Geologorum Poloniae* **89**, 291–305.

Kozur, H.W., Weems, R.E., 2010. The biostratigraphic importance of conchostracans in the continental Triassic of the northern hemisphere. *Geological Society, London, Special Publications* **334**, 315–417.

Kürschner, W.M., Herngreen, G.W., 2010. Triassic palynology of central and northwestern Europe: a review of palynofloral diversity patterns and biostratigraphic subdivisions. *Geological Society, London, Special Publications* **334**, 263–283.

Langer, M.C., et al. 2013. Non-dinosaurian Dinosauromorpha. In: Nebitt, S.J., et al. (Eds.), *Anatomy, Phylogeny and Palaeobiology of Early Archosaurs and their Kin. Geological Society, London, Special Publications* **379**, 157–186.

Lindström, S., et al. 2021. Tracing volcanic emissions from the Central Atlantic Magmatic Province in the sedimentary record. *Earth-Science Reviews* **212**, 103444.

Loinaze, V.S.P., et al. 2018. Palaeobotany and palynology of coprolites from the Late Triassic Chañares Formation of Argentina: implications for vegetation provinces and the diet of dicynodonts. *Palaeogeography, Palaeoclimatology, Palaeoecology* **502**, 31–51.

Lucas, S.G., 2015. Age and correlation of late Triassic Tetrapods from Southern Poland. *Annales Societatis Geologorum Poloniae* **85**, 627–635.

Lund, J.J., 1977. Rhaetic to Lower Liassic palynology of the onshore south-eastern North Sea Basin. *Danmarks Geologiske Undersøgelse II. Række* **109**, 1–128.

Marcinkiewicz, T., 1957. Megaspores of the Lias from Praszka, Zawiercie and Święty Krzyż Mountains. *Kwartalnik Geologiczny* **1**, 299–302 (In Polish, English summary).Marcinkiewicz, T., 1981. W sprawie megaspore z Lipia Śląskiego koło Lublińca (On the question of megaspores from Lipie Śląskie). *Przegląd Geologiczny* **39**, 419–420 (in Polish).

Marynowski, L., Simoneit, B.R.T., 2009. Widespread Upper Triassic to Lower Jurassic wildfire records from Poland: evidence from charcoal and pyrolytic polycyclic aromatic hydrocarbons. *Palaios* **24**, 785–798.

Marynowski, L., et al. 2011. First multi-proxy record of Jurassic wildfires from Gondwana: evidence from the Middle Jurassic of the Neuquén Basin, Argentina. *Palaeogeography, Palaeoclimatology and Palaeoecology* **299**, 129–136.

Marynowski, L., et al. 2013. Perylene as an indicator of conifer fossil wood degradation by wood-degrading fungi. *Organic Geochemistry* **59**, 143–151.

Meyen, S.V., 1987. *Fundamentals of Palaeobotany*. London: Chapman and Hall.

Nesbitt, S.J., et al. 2013. The oldest dinosaur? A Middle Triassic dinosauriform from Tanzania. *Biology Letters* **9**, 20120949 https://doi.org/10.1098/rsbl.2008.0441

Niedźwiedzki, G., Budziszewska-Karwowska, E., 2018. A new occurrence of the Late Triassic archosaur Smok in southern Poland. *Acta Palaeontologica Polonica* **68,** 703–712.

Niedźwiedzki, G., 2011. Tropy dinozaurów z wczesnojurajskiego ekosystemu z Sołtykowa w Górach Świętokrzyskich (Dinosaur tracks from the Early Jurassic ecosystem of Sołtyków, Holy Cross Mountains). *Biuletyn Państwowego Instytutu Geologicznego* **447**, 49–98 (in Polish, English summary).

Niedźwiedzki, G., 2013. The large predatory archosaur *Smok wawelski* from the latest Triassic of Poland. (University of Warsaw, 2013). Unpublished PhD Thesis.

Niedźwiedzki, G., et al. 2014. Basal dinosauriform and theropod dinosaurs from the mid–late Norian (Late Triassic) of Poland: implications for Triassic dinosaur evolution and distribution. *Palaeontology* **57**, 1121–1142.

Niedźwiedzki, G., et al. 2012. A large predatory archosaur from the Late Triassic of Poland. *Acta Palaeontologica Polonica* **57**, 267–276.

Niedźwiedzki, G., et al. 2011. Bite traces on dicynodont bones and the early evolution of large terrestrial predators. *Lethaia* **44**, 87–92.

Niedźwiedzki, G., et al. 2016. Reduction of vertebrate coprolite diversity associated with the end-Permian extinction event in Vyazniki region, European Russia. *Palaeogeography, Palaeoclimatology, Palaeoecology* **450**, 77–90.

Northwood, C., 2005. Early Triassic coprolites from Australia and their palaeobiological significance. *Palaeontology* **48**, 49–68.

Ociepa, A.M., et al. 2008. Nowe stanowiska *Lepidopteris ottonis* (Geoppert) Schimper w Polsce (New localities of *Lepidopteris ottonis* (Geoppert) Schimper in Poland). *Kwartalnik AGH Geologia* **34**, 199–200 (in Polish).

Oldham, T., 1976. Flora of the Wealden plant debris beds of England. *Palaeontology* **19**, 473–502

Olempska, E., 2004. Late Triassic spinicaudatan crustaceans from southwestern Poland. *Acta Palaeontologica Polonica* **49**, 429–442.

Orłowska-Zwolińska, T., 1962. Granica między retykiem i liasem w świetle badań sporowo-pyłkowych (The boundary between the Rhaetian and Liassic in the light of spore-pollen studies). *Kwartalnik Geologiczny* **6**, 729–730 (in Polish).

Orłowska-Zwolińska, T., 1983. Palinostratygrafia epikontynentalnych osadów wyższego triasu w Polsce (Palynostratigraphy of epicontinental Upper Triassic deposits in Poland). *Prace Instytutu Geologicznego* **54**, 1–88 (in Polish, English summary).

Orłowska-Zwolińska, T., 1985. Palynological zones of the Polish epicontinental Triassic. *Bulletin de l'Académie Polonaise des Sciences, Série des Sciences de la Terre* **33**, 107–117.

Otto, A., Simoneit, B.R.T., 2001. Chemosystematics and diagenesis of terpenoids in fossil conifer species and sediment from the Eocene Zeitz Formation, Saxony, Germany. *Geochimica et Cosmochimica Acta* **65**, 3505–3527.

Otto, A., Wilde, V., 2001. Sesqui-, di- and triterpenoids as chemosystematic markers in extant conifers – A review. *Botanical Review* **67**, 141–238.

Otto, A., et al. 2007. Terpenoids as chemosystematic markers in selected fossil and extant species of pine (*Pinus*, Pinaceae). *Botanical Journal of the Linnean Society* **154**, 129–140.

Pacyna, G., 2014. Plant remains from the Polish Triassic. Present knowledge and future prospects. *Acta Palaeobotanica* **54**, 3–33.

Pacyna, G., 2019. Sphenopsid and fern remains from the Upper Triassic of Krasiejów (SW Poland). *Annales Societatis Geologorum Poloniae* **89**, 307–316.

Pacyna, G., et al. 2017. A new conifer from the Upper Triassic of southern Poland linking the advanced voltzialean type of ovuliferous scale with *Brachyphyllum-Pagiophyllum*-like leaves. *Review of Palaeobotany and Palynology* **245**, 28–54. http://10.1016/j.revpalbo.2017.05.015

Paul, S., Dutta, S., 2020. Contrasting biomarker signatures of Cretaceous and Early Paleogene coal-bearing sediments during the northward flight of India. In: Biological Consequences of Plate Tectonics: New Perspectives on Post-Gondwana Break-up—A Tribute to Ashok Sahni, Prasad, G.V.R., Patnaik, R. (Eds.), *Vertebrate Paleobiology and Paleoanthropology Series*, Springer Nature, 265–278.

Pawlak, W., et al. 2022. Triassic fish faunas from Miedary (Upper Silesia, Poland) and their implications for understanding paleosalinity. *Palaeogeography, Palaeoclimatology, Palaeoecology* **590**, 110860.

Peters, K.E., et al. 2005. *The Biomarker Guide*. Cambridge University Press, vol. 1 and 2.

Petersen, H.I., Lindström, S., 2012. Synchronous wildfire activity rise and mire deforestation at the Triassic–Jurassic boundary. *PLoS ONE* **7**, e47236.

Pieńkowski, G., 1998. Dinosaur nesting ground from the Early Jurassic fluvial deposits, Holy Cross Mountains (Poland). *Geological Quarterly* **42**, 461–476.

Pieńkowski, G., 1999. Dinosaur nesting ground from the Early Jurassic fluvial deposits, Holy Cross Mountains (Poland): reply and new evidence. *Geological Quarterly* **43**, 379–382.

Pieńkowski, G., 2004a. The epicontinental Lower Jurassic of Poland. *Polish Geological Institute Special Papers* **12**, 1–122.

Pieńkowski, G., 2004b. Sołtyków, Poland: a unique palaeoecological record of the Early Jurassic continental deposits. *Volumina Jurassica* **2**, 1–16.

Pieńkowski, G., et al. 2014. Climatic reversals related to the Central Atlantic magmatic province caused the end-Triassic biotic crisis—evidence from continental strata in Poland. Volcanism, Impacts, and Mass Extinctions: Causes and Effects. *Geological Society of America, Special Papers* **505**, 263–286.

Poage III, G.W., et al. 2000. Activated charcoal attenuates bitterweed toxicosis in sheep. *J. Range Manage* **53**, 73–78.

Qvarnström, M., et al. 2017. Synchrotron phase-contrast microtomography of coprolites generates novel palaeobiological data. *Scientific Reports* **7**, 2723.

Qvarnström, M., et al. 2019b. Beetle-bearing coprolites possibly reveal the diet of a Late Triassic dinosauriform. *Royal Society Open Science* **6**, 181042.

Qvarnström, M., et al. 2021. Exceptionally preserved beetles in a Triassic coprolite of putative dinosauriform origin. *Current Biology* **31**, 3374–3381.

Qvarnström, M., et al. 2022. Scrambled eggs: synchrotron scans of eggs from the earliest Jurassic seem to reveal unusual traits in early dinosaur embryos. In: Ehret, C., et al. (Eds.), *Program and abstract volume of the 82nd meeting of the Society of Vertebrate Paleontology*.

Qvarnström, M., et al. 2019a. Tyrannosaurid-like osteophagy by a Triassic archosaur. *Scientific Reports* **9**, 1–9.

Qvarnström, M., et al. 2016. Vertebrate coprolites (fossil faeces): an underexplored Konservat-Lagerstätte. *Earth-Science Reviews* **162**, 44–57.

Qvarnström, M., et al. 2024. Supplementary figures to "Fossil digestive contents and trophic dynamics record the advent of dinosaur supremacy". Figshare. https://doi.org/10.6084/m9.figshare.26103031

Reymanówna, M., 1992. Two conifers from the Liassic flora of Odrowąż in Poland. In: Kovar-Eder, J., (Ed.), *Palaeovegetational development in Europe and regions relevant to its palaeofloristic evolution. Proceedings of the Pan-European Palaeobotanical Conference. Museum of Natural History, Vienna*, 307–311.

Rogers, R.R., et al. 1993. The Ischigualasto tetrapod assemblage (Late Triassic, Argentina) and ^{40}Ar/^{39}Ar dating of dinosaur origins. *Science* **260**, 794–797.

Rybicki, M., et al. 2017. Age and origin of the well-preserved organic matter in internal sediments from the Silesian-Cracow Lead-Zinc deposits, Southern Poland. *Economic Geology* **112**, 775–798.

Sadlok, G., Wawrzyniak, Z., 2013. Upper Triassic vertebrate tracks from Kraków-Częstochowa Upland, Southern Poland. *Annales Societatis Geologorum Poloniae* **83**, 105–111.

Sadlok, G., 2022. Carnivorous reptile feeding strategies and postmortem food-processing behaviors: tooth traces on bones from the Upper Triassic Grabowa Formation (southern Poland). *Journal of Paleontology* **96**, 1189–1208.

Samsonowicz, J., 1929. Cechsztyn, trias i lias na północnym zboczu Łysogór (Les Zechstein, le Trias et le Liasique sur le versant nord du Massif de S-te Croix). *Sprawozdania Państwowego Instytutu Geologicznego* **5**, 281 pp. (in Polish, French summary).

Schoch, R.R., 2012. A dicynodont mandible from the Triassic of Germany forms the first

evidence of large herbivores in the Central European Carnian. *Neues Jahrbuch fur Geologie und Palaontologie-Abhandlungen* **263**, 119–123.

Seilacher, A., et al. 2001. A fresh look at sideritic “coprolites”. *Paleobiology* **27**, 7–13.

Shukla, U.K., et al. 2010. Facies architecture of the Stuttgart Formation (Schilfsandstein, Upper Triassic), central Germany, and its comparison with modern Ganga system, India. *Palaeogeography, Palaeoclimatology, Palaeoecology* **297**, 110–128.

Simoneit, B.R.T., 2002. Biomass burning - a review of organic tracers for smoke from incomplete combustion. *Applied Geochemistry* **17**, 129–162.

Simoneit, B.R.T., et al. 1999. Levoglucosan, a tracer for cellulose in biomass burning and atmospheric particles. *Atmospheric Environment* **33**, 173–182.

Skawiński, T., et al. 2017. A re-evaluation of the historical ‘dinosaur’remains from the Middle-Upper Triassic of Poland. *Historical Biology* **29**, 442–472.

Skrzycki, P., 2015. New species of lungfish (Sarcopterygii, Dipnoi) from the Late Triassic Krasiejów site in Poland, with remarks on the ontogeny of Triassic dipnoan tooth plates. *Journal of Vertebrate Paleontology* **35**, e964357. https://doi.org/10.1080/02724634.2015.964357

Smith, D.M., Marcot, J.D., 2015. The fossil record and macroevolutionary history of the beetles. *Proceedings of the Royal Society B: Biological Sciences* **282**, 20150060.

Staneczko, K., 2007. Nowe dane paleobotaniczne na temat górnego triasu z Lipia Śląskiego koło Lublińca (południowa Polska) (New paleobotanical data on the Upper Triassic from Lipie Śląskie near Lubliniec (southern Poland)). *Geo−Sympozjum Młodych Badaczy Silesia 2007*, 155–168 (in Polish).

Stockey, R.A., 1994. Mesozoic araucariaceae: Morphology and systematic relationships. *Journal of Plant Research* **107**, 493–502.

Sulej, T., et al. 2011. A new dicynodont-archosaur assemblage from the Late Triassic (Carnian) of Poland. *Earth and Environmental Science Transactions of The Royal Society of Edinburgh* **101,** 261–269. https://doi.org/10.1017/S1755691011020123

Sulej, T., et al. 2012. A new Late Triassic vertebrate fauna from Poland with turtles, aetosaurs, and coelophysoid dinosaurs. *Journal of Vertebrate Paleontology* **32**, 1033–1041.

Sulej, T., et al. 2020. A new early Late Triassic non-mammaliaform eucynodont from Poland. *Historical Biology* **32**, 80–92. https://doi.org/10.1080/08912963.2018.1471477

Sulej, T., et al. 2021. Eucynodont teeth from the Late Triassic of Krasiejów, Southern Poland. *Historical Biology* **33**, 3633–3640.

Sulej, T., Niedźwiedzki, G., 2019. An elephant-sized Late Triassic synapsid with erect limbs. *Science* **363**, 78–80.

Świło, M., et al. 2014. Mammal-like tooth from the Upper Triassic of Poland. *Acta Palaeontologica Polonica* **59**, 815–820. http://dx.doi.org/10.4202/app.00016.2013

Szczygielski, T., Sulej, T., 2023. *Woznikella triradiata* n. gen., n. sp. – a new kannemeyeriiform dicynodont from the Late Triassic of northern Pangea and the global distribution of Triassic dicynodonts. *Comptes Rendus Palevol* **22**, 279–406.

Szczygielski, T., Sulej, T., 2016. Revision of the Triassic European turtles *Proterochersis* and *Murrhardtia* (Reptilia, Testudinata, Proterochersidae), with the description of new taxa from Poland and Germany. *Zoological Journal of the Linnean Society* **177**, 395–427.

Szulc, J., et al. 2006. The Upper Triassic crenogenic limestones in Upper Silesia (southern

Poland) and their paleoenvironmental context. In: Alonso-Zarza, A.M., Tanner, L.H. (Eds.). *Paleoenvironmental record and applications of calcretes and palustrine carbonates*. *Geological Society of America, Special Paper* **416**, 133–151.

Szulc, J., et al. 2015. How many Upper Triassic bone-bearing levels are there in Upper Silesia (Southern Poland)? A critical overview of stratigraphy and facies. *Annales Societatis Geologorum Poloniae* **85**, 587–626.

Tweet, J.S., et al. 2008. Probable gut contents within a specimen of Brachylophosaurus canadensis (Dinosauria: Hadrosauridae) from the Upper Cretaceous Judith River Formation of Montana. *Palaios* **23**, 625–636.

Watson, J., 1988. The Cheirolepidiaceae. In: Beck, Ch., (Ed.), *Origin and evolution of Gymnosperms*. Columbia University Press, New York, 382–447.

Wawrzyniak, Z., Ziaja, J., 2009. Wstępne wyniki badań górnotriasowej makroflory Lipia Śląskiego, Polska (Preliminary results of research of the Upper Triassic macroflora from Lipie Śląskie, Poland). *Kwartalnik AGH Geologia* **35**, 105–106 (In Polish, English abstract).

Wawrzyniak, Z., Ziaja, J., 2010. The Upper Triassic Coniferales from Lipie Śląskie (Poland). In: *8th European Palaeobotany-Palynology Conference, 6–10 July, Budapest, Hungary, Program and Abstracts*, 252.

Wawrzyniak, Z., 2010a. Tafonomia późnotriasowej flory z Lipia Śląskiego: 42. In: Dubicka, Z., et al. (Eds.), *II Konferencja Naukowa Młodzi w Paleontologii, Materiały konferencyjne,* Warszawa (in Polish).

Wawrzyniak, Z., 2010b. Późnotriasowa flora z Lipia Śląskiego (Late Triassic flora from Lipie Śląskie). Unpublished MSc Thesis, Wydział Nauk o Ziemi, Uniwersytet Śląski, Sosnowiec, (in Polish, English abstract).

Wawrzyniak, Z., 2010c. Późnotriasowe kutykule z Lipia Śląskiego k. Lublińca (Late Triassic cuticles from Lipie Śląski near Lubliniec). In: *Materiały z XXI Konferencji Naukowej Sekcji Paleontologicznej PTG „Kopalne biocenozy w czasie i przestrzeni", Żarki – Letnisko*, 85 (in Polish).

Wawrzyniak, Z., 2010d. What could eat dicynodonts (Dicynodontia, Therapsida) from Lipie Śląskie? (Upper Silesia, Poland) – preliminary results of research of the Late Triassic macroflora. In: Nowakowski D. (Ed.), *Morphology and Systematics of Fossil Vertebrates*. DN Publisher, Wrocław, 118–124.

Wawrzyniak, Z., 2011. The Upper Triassic cuticles from Lipie Śląskie (South Poland). In: Bąk, M., et al. (Eds.), *Integrating Microfossil Records from the Oceans and Epicontinental Seas. Grzybowski Foundation Special Publication* **17**, 140–141.

Wawrzyniak, Z., 2023. Late Triassic Flora of Southern Poland. Późnotriasowa flora południowej Polski. Unpublished PhD Thesis.

Wawrzyniak, Z., Filipiak, P., 2023. Fossil floral assemblage from the Upper Triassic Grabowa Formation (Upper Silesia, southern Poland). *Annales Societatis Geologorum Poloniae* **93**, 165–193.

Wcisło-Luraniec, E., 1991. The Lower Liassic flora from Odrowąż in Poland and its ecosystem. *Contributions from the Palaeontological Museum, University of Oslo* **364**, 69–70.

Zatoń, M., et al. 2015. Coprolites of Late Triassic carnivorous vertebrates from Poland: an integrative approach. *Palaeogeography, Palaeoclimatology, Palaeoecology* **430**, 21–46.

Zatoń, M., et al. 2005. Late Triassic charophytes around the bone-bearing bed at Krasiejów (SW

Poland) – palaeoecological and environmental remarks. *Acta Geologica Polonica* **55**, 283–293.

Zalewski, J., et al. (in prep.) Late Triassic freshwater sharks from of Lisowice-Lipie Śląskie (Poland).

Ziaja, J., 2006. Lower Jurassic spores and pollen grains from Odrowąż, Mesozoic margin of the Holy Cross Mountains, Poland. *Acta Palaeobotanica* **46**, 3–83.
