# Supplementary material for: Digestive contents and food webs record the advent of dinosaur supremacy
Source: Nature. 2024 Nov 27;636(8042):397–403. doi: 10.1038/s41586-024-08265-4 (PMC11634772; doi:10.1038/s41586-024-08265-4)
Supplement: Supplementary file 2 — Reporting Summary [file 41586_2024_8265_MOESM2_ESM.pdf]

nature portfolio

Corresponding author(s): Martin Qvarnström and Grzegorz Niedźwiedzki

Last updated by author(s): Sep 11, 2024

Reporting Summary

Nature Portfolio wishes to improve the reproducibility of the work that we publish. This form provides structure for consistency and transparency in reporting. For further information on Nature Portfolio policies, see our Editorial Policies and the Editorial Policy Checklist.

Statistics

For all statistical analyses, confirm that the following items are present in the figure legend, table legend, main text, or Methods section.

n/a	Confirmed	
☒	☐	The exact sample size (n) for each experimental group/condition, given as a discrete number and unit of measurement
☒	☐	A statement on whether measurements were taken from distinct samples or whether the same sample was measured repeatedly
☒	☐	The statistical test(s) used AND whether they are one- or two-sided<br>*Only common tests should be described solely by name; describe more complex techniques in the Methods section.*
☒	☐	A description of all covariates tested
☒	☐	A description of any assumptions or corrections, such as tests of normality and adjustment for multiple comparisons
☒	☐	A full description of the statistical parameters including central tendency (e.g. means) or other basic estimates (e.g. regression coefficient) AND variation (e.g. standard deviation) or associated estimates of uncertainty (e.g. confidence intervals)
☒	☐	For null hypothesis testing, the test statistic (e.g. F, t, r) with confidence intervals, effect sizes, degrees of freedom and P value noted<br>*Give P values as exact values whenever suitable.*
☐	☐	For Bayesian analysis, information on the choice of priors and Markov chain Monte Carlo settings
☒	☐	For hierarchical and complex designs, identification of the appropriate level for tests and full reporting of outcomes
☒	☐	Estimates of effect sizes (e.g. Cohen's d, Pearson's r), indicating how they were calculated

Our web collection on statistics for biologists contains articles on many of the points above.

Software and code

Policy information about availability of computer code

Data collection	Software used at beamline ID19, European Synchrotron Radiation Facility (France), NIST 17, NIS-Elements software
Data analysis	e.g. VGStudio MAX version 3.1, Illustrator/Photoshop, CorelDraw, various software associated to microscopy and geochemistry

For manuscripts utilizing custom algorithms or software that are central to the research but not yet described in published literature, software must be made available to editors and reviewers. We strongly encourage code deposition in a community repository (e.g. GitHub). See the Nature Portfolio guidelines for submitting code & software for further information.

Data

Policy information about availability of data

All manuscripts must include a data availability statement. This statement should provide the following information, where applicable:

- Accession codes, unique identifiers, or web links for publicly available datasets
- A description of any restrictions on data availability
- For clinical datasets or third party data, please ensure that the statement adheres to our policy

Reconstructed image stacks of the synchrotron-scanned bromalites will upon publication be publicly available in ESRF's heritage database for palaeontology, evolutionary biology and archaeology: http://paleo.esrf.eu/. Geological/palaeobotanical samples, all studied bromalite specimens, and bone with bite marks are housed in the scientific collection at the Polish Geological Institute-National Research Institute (Warszawa, Kielce; acronym Muz. PGI; Muz. PGI OS), Institute of Paleobiology, Polish Academy of Sciences (Warszawa; acronym ZPAL), in the collections of research results at the University of Silesia (Sosnowiec; palaeobotanical

data), in Paleobotanical collection Palaeozoic and Mesozoic of the National Biodiversity Collection – Herbarium KRAM at W. Szafer Institute of Botany, Polish Academy of Sciences, Cracow, Poland (KRAM) and Jagiellonian University (Kraków; palaeobotanical data).

Research involving human participants, their data, or biological material

Policy information about studies with human participants or human data. See also policy information about sex, gender (identity/presentation), and sexual orientation and race, ethnicity and racism.

Reporting on sex and gender	*Use the terms sex (biological attribute) and gender (shaped by social and cultural circumstances) carefully in order to avoid confusing both terms. Indicate if findings apply to only one sex or gender; describe whether sex and gender were considered in study design; whether sex and/or gender was determined based on self-reporting or assigned and methods used. Provide in the source data disaggregated sex and gender data, where this information has been collected, and if consent has been obtained for sharing of individual-level data; provide overall numbers in this Reporting Summary. Please state if this information has not been collected. Report sex- and gender-based analyses where performed, justify reasons for lack of sex- and gender-based analysis.*
Reporting on race, ethnicity, or other socially relevant groupings	*Please specify the socially constructed or socially relevant categorization variable(s) used in your manuscript and explain why they were used. Please note that such variables should not be used as proxies for other socially constructed/relevant variables (for example, race or ethnicity should not be used as a proxy for socioeconomic status). Provide clear definitions of the relevant terms used, how they were provided (by the participants/respondents, the researchers, or third parties), and the method(s) used to classify people into the different categories (e.g. self-report, census or administrative data, social media data, etc.) Please provide details about how you controlled for confounding variables in your analyses.*
Population characteristics	*Describe the covariate-relevant population characteristics of the human research participants (e.g. age, genotypic information, past and current diagnosis and treatment categories). If you filled out the behavioural & social sciences study design questions and have nothing to add here, write "See above."*
Recruitment	*Describe how participants were recruited. Outline any potential self-selection bias or other biases that may be present and how these are likely to impact results.*
Ethics oversight	*Identify the organization(s) that approved the study protocol.*

Note that full information on the approval of the study protocol must also be provided in the manuscript.

Field-specific reporting

Please select the one below that is the best fit for your research. If you are not sure, read the appropriate sections before making your selection.

☐ Life sciences ☐ Behavioural & social sciences ☒ Ecological, evolutionary & environmental sciences

For a reference copy of the document with all sections, see nature.com/documents/nr-reporting-summary-flat.pdf

Ecological, evolutionary & environmental sciences study design

All studies must disclose on these points even when the disclosure is negative.

Study description	Study of fossil specimens across the Upper Triassic to Lower Jurassic interval in the Polish Basin
Research sample	Hundreds of fossils with direct evidence of feeding (including coprolites (fossil droppings), regurgitalites (fossil regurgitates), and bite-marked bones) plant fossils, and geological samples.
Sampling strategy	All different kinds of bromalites were analysed in order to study all possible ecological interactions
Data collection	The bromalites derive from natural or artificial Upper Triassic and Lower Jurassic sites exposures located in Silesia and Holy Cross Mts. in the Polish Basin area (see Supp. Fig. 1). A total of 532 bromalites have been collected from eight fossiliferous sites (Supp. Tabs. 2-9). The specimens were collected between 1996 and 2017 by G.N., T.S., K.O., G.Pi., and M.Q.
Timing and spatial scale	Fieldwork: 1996 and 2017. Synchrotron data were collected in two scanning sessions during 2016. Data analysis collection and analyses have been ongoing since.
Data exclusions	No data was excluded from the analysis
Reproducibility	All methods are described carefully, samples are stored in appropriate collections, and imaging data will be publically available.
Randomization	Not relevant for this study of fossil specimens
Blinding	Not relevant for this study of fossil specimens

Did the study involve field work? ☒ Yes ☐ No

Field work, collection and transport

Field conditions	Fieldwork was conducted during many field seasons, predominantly during summer months.
Location	Upper Triassic and Lower Jurassic sites exposures located in Silesia and Holy Cross Mts. in the Polish Basin area (see manuscript for details)
Access & import/export	Necessary permits were acquired from local governments for the fieldwork at the sites.
Disturbance	Disturbance was minimal during fieldwork

Reporting for specific materials, systems and methods

We require information from authors about some types of materials, experimental systems and methods used in many studies. Here, indicate whether each material, system or method listed is relevant to your study. If you are not sure if a list item applies to your research, read the appropriate section before selecting a response.

Materials & experimental systems

n/a	Involved in the study	
☒	☐	Antibodies
☒	☐	Eukaryotic cell lines
☐	☒	Palaeontology and archaeology
☒	☐	Animals and other organisms
☒	☐	Clinical data
☒	☐	Dual use research of concern
☒	☐	Plants

Methods

n/a	Involved in the study	
☒	☐	ChIP-seq
☒	☐	Flow cytometry
☒	☐	MRI-based neuroimaging

Palaeontology and Archaeology

Specimen provenance	The specimens derive from Upper Triassic and Lower Jurassic sites exposures located in Silesia and Holy Cross Mts. in the Polish Basin area (see manuscript for details). Permits from the local government were obtained for fieldwork.
Specimen deposition	Geological/palaeobotanical samples, all studied bromalite specimens, and bone with bite marks are housed in the scientific collection at the Polish Geological Institute-National Research Institute (Warszawa, Kielce; acronym Muz. PGI; Muz. PGI OS), Institute of Paleobiology, Polish Academy of Sciences (Warszawa; acronym ZPAL), in the collections of research results at the University of Silesia (Sosnowiec; palaeobotanical data), in Paleobotanical collection Palaeozoic and Mesozoic of the National Biodiversity Collection – Herbarium KRAM at W. Szafer Institute of Botany, Polish Academy of Sciences, Cracow, Poland (KRAM) and Jagiellonian University (Kraków; palaeobotanical data).
Dating methods	No new dating data are provided

☐ Tick this box to confirm that the raw and calibrated dates are available in the paper or in Supplementary Information.

Ethics oversight	*Identify the organization(s) that approved or provided guidance on the study protocol, OR state that no ethical approval or guidance was required and explain why not.*

Note that full information on the approval of the study protocol must also be provided in the manuscript.

Plants

Seed stocks	*Report on the source of all seed stocks or other plant material used. If applicable, state the seed stock centre and catalogue number. If plant specimens were collected from the field, describe the collection location, date and sampling procedures.*
Novel plant genotypes	*Describe the methods by which all novel plant genotypes were produced. This includes those generated by transgenic approaches, gene editing, chemical/radiation-based mutagenesis and hybridization. For transgenic lines, describe the transformation method, the number of independent lines analyzed and the generation upon which experiments were performed. For gene-edited lines, describe the editor used, the endogenous sequence targeted for editing, the targeting guide RNA sequence (if applicable) and how the editor was applied.*
Authentication	*Describe any authentication procedures for each seed stock used or novel genotype generated. Describe any experiments used to assess the effect of a mutation and, where applicable, how potential secondary effects (e.g. second site T-DNA insertions, mosiacism, off-target gene editing) were examined.*
