# Supplementary material for: Digestive contents and food webs record the advent of dinosaur supremacy
Source: Nature. 2024 Nov 27;636(8042):397–403. doi: 10.1038/s41586-024-08265-4 (PMC11634772; doi:10.1038/s41586-024-08265-4)
Supplement: Supplementary file 3 — Supplementary Tables 1–13. These contain information on the synchrotron scanning parameters (Table 1), floras and faunas of studied localities (Table 2), lists of studied specimens (Tables 3–9), geochemical data (Tables 10–12) and sedimentological and taphonomic characteristics of the sites (Table 13). [file 41586_2024_8265_MOESM3_ESM.pdf]

Supplementary Tables 1-13 for:

Digestive contents and food webs record the advent of dinosaur supremacy

Martin Qvarnström[1]*, Joel Vikberg Wernström[1,2], Zuzanna Wawrzyniak[3], Maria Barbacka[4,5], Grzegorz Pacyna[6], Artur Górecki[6], Jadwiga Ziaja[5], Agata Jarzynka[7], Krzysztof Owocki[8], Tomasz Sulej[8], Leszek Marynowski[3], Grzegorz Pieńkowski[9,#], Per E. Ahlberg[1] & Grzegorz Niedźwiedzki[1,9]*

[1]Department of Organismal Biology, Evolutionary Biology Centre, Uppsala University, Norbyvägen 18A, 752 36 Uppsala, Sweden; [2]The Arctic University Museum of Norway (UMAK), UiT The Arctic University of Norway, Lars Thørings veg 10, 9006, Tromsø, Norway; [3]Institute of Earth Sciences, Faculty of Natural Sciences, University of Silesia in Katowice, Będzińska 60, 41-200 Sosnowiec, Poland; [4]Hungarian Natural History Museum, Botany Department, 1431 Budapest, Pf. 137, Hungary; [5]W. Szafer Institute of Botany, Polish Academy of Sciences, Lubicz 46, 31-512 Kraków, Poland,; [6]Institute of Botany, Department of Taxonomy, Phytogeography and Palaeobotany, Faculty of Biology, Jagiellonian University, Gronostajowa 3, 30-387 Kraków, Poland; [7]Institute of Geological Sciences, Polish Academy of Sciences, Research Centre in Kraków, Senacka 1, 30-002 Kraków, Poland; [8]Institute of Paleobiology, Polish Academy of Sciences, Twarda 51/55, 00-818 Warsaw, Poland; [9]Polish Geological Institute – National Research Institute, Rakowiecka 4, 00-975 Warsaw, Poland

#Deceased April 19, 2023

*Corresponding authors. Email: martin.qvarnstrom@ebc.uu.se; grzegorz.niedzwiedzki@ebc.uu.se

SUPPLEMENTARY TABLES

Supplementary Table 1. Synchrotron scan parameters. Bromalite were scanned using propagation phase-contrast synchrotron microtomography (PPC-SRµCT) as a part of project ES145 at beamline ID19 of the European Synchrotron Radiation Facility (ESRF) in Grenoble, France.

**VOXEL SIZE (µM)**	6,54	13,83	6,36	13,4
**OPTIC**	Hasselblad 100mm / 100mm	Hasselblad 100mm / 210mm	Hasselblad 100mm/100mm diaphragm 20mm	Hasselblad 100mm / 210mm diaphragm 20 mm
**AVERAGE ENERGY (KEV)**	111	126	112	113
**FILTERS (MM)**	Al 5.6<br>Cu 5	Al 5.6<br>Cu 6	Al 2.8<br>Cu 6	Al 2.8<br>Cu 6
**PROPAGATION DISTANCE (MM)**	2800	2800	2800	2800
**SENSOR**	sCMOS PCO edge 5.5	sCMOS PCO edge 5.5	sCMOS PCO edge 5.5	sCMOS PCO edge 5.5
**SCINTILLATOR**	GGG:Eu 1000 µm	GGG:Eu 1000 µm	LuAG:Ce 500	GGG:Eu 1000 µm
**INSERTION DEVICE**	W150	W150	W150	W150
**ID GAP (MM)**	48	39	51	50
**PROJECTION NUMBER**	6000	6000	6000	6000
**SCAN GEOMETRY**	360°, half-acquisition, vertical series 5mm / 3.8 mm	360°, half-acquisition, vertical series 5mm / 3.8 mm	360°, half-acquisition, vertical series 4 mm	360°, half-acquisition, vertical series 4 mm
**SUBFRAME TIME**	0,02	0,01	0,05	0,02
**EXPOSURE TIME (S)**	0,08	0,05	0,05	0,02
**ACCUMULATION LEVEL**	4	5	1	1
**TIME PER SCAN (MIN)**	9,6	6,6	6,1	3,6
**RECONSTRUCTION**	phase retrieval	phase retrieval	phase retrieval	phase retrieval

Supplementary Table 2: Composition of floras and faunas in the Late Triassic and earliest Jurassic of Poland. The numbers given next to the taxon names refer to the identifications shown in Figs. 1 and 3 (main text).

Locality	Age/Assemblage	Fossil record	Described plant taxa	Described vertebrate taxa/ichnotaxa/fossils	Major studies
**Krasiejów**	Middle-Late Carnian (Late Triassic); Krasiejów-Woźniki assemblage	Plants (macroremains, palynomorphs preserved in bromalites); invertebrates (bivalves, ostracods, conchostracans, insects, cyclidans); fish (sharks, actinopterygians, sarcopterygians); tetrapods (temnospondyls, archosauromorphs, therapsids); trace fossils (bromalites, tetrapod tracks, invertebrate traces, bite marks).	*Neocalamites merianii*<br>*Sphenopteris* sp.<br>*Glyptolepis* sp.<br>*Pseudohirmerella* sp.<br>*Pachylepis* sp.<br>*Desmiophyllum* sp.<br>*Pterophyllum* sp.	**Trace fossil record:**<br><br>Tetrapod tracks<br>[*Brachychirotherium* isp. (13, 14),<br>*Atreipus* isp. (12),<br>Tetrapoda track indet. (?)]<br><br>Bromalites, morphotypes M1-M7<br>(for more details see Supp. Tab. 3)<br><br>Bite marks (osteoderms of<br>*Stagonolepis*, *Silesunio* shells)<br><br>**Body fossil record:**<br><br>*Lonchidion* sp. (3)<br>Siemionotiformes indet. (1)<br>Polypteriformes indet. (2)<br>Semionotidae indet. (1)<br>Palaeoniscidae indet. (1)<br>Redfieldiidae indet. (1)<br>? Acanthopterygii indet. or<br>? Euteleostei indet. (1)<br>cf. *Saurichthys* sp. (2)<br>*Gyrolepis* sp. (1)<br>cf. *Severnichthys* sp. (2)<br>cf. *Arganodus* sp. (4)<br>Dipnoa indet. (4)<br>*Ptychoceratodus roemeri* (4)<br>Coelacanthiformes indet. (5)<br>*Metoposaurus krasiejowensis* (7)<br>*Cyclotosaurus intermedius* (8)<br>Temnospondyli indet. 1 (?)<br>Temnospondyli indet. 2 (?)<br>Temnospondyli indet. 3 (?)<br>Temnospondyli indet. 4 (?)<br>Rhynchocephalia indet. 1<br>(10)Rhynchocephalia indet. 2 (10)<br>cf. *Planocephalosaurus* sp. 1 (10)<br>cf. *Planocephalosaurus* sp. 2 (10)<br>cf. *Diphydontosaurus* sp. (10)<br>Sphenodontia indet. (10)<br>cf. *Clevosaurus* sp. (10)<br>cf. Tanystropheidae indet. (?)<br>Archosauriformes indet. 1 (?)<br>Archosauriformes indet. 2 (?)<br>Archosauriformes indet. 3 (?)<br>Archosauriformes indet. 4 (?)<br>Archosauriformes indet. 5 (?)<br>Archosauriformes indet. 6 (?)<br>Archosauriformes indet. 7 (?)<br>cf. *Protecovasaurus* sp. (?)<br>cf. Crocodylomorpha indet. (?)<br>Pseudosuchia indet. 1 (?)<br>Pseudosuchia indet. 2 (?)<br>cf. Sauropodomorpha indet. (?)<br>cf. *Revueltosaurus* sp. (?)<br>Theropoda indet. (?)<br>*Polonodon* sp. (15)<br>*Ozimek volans* (11)<br>*Paleorhinus* sp. (9)<br>*Stagonolepis olenkae* (13)<br>*Polonosuchus silesiacus* (14)<br>*Silesaurus opolensis* (12)	Dzik et al. 2000,<br>Dzik 2001, 2003<br>Sulej 2002, 2003, 2004, 2005, 2007, 2010, Dzik & Sulej 2004, 2007, 2016, Olempska 2004, Sulej & Majer 2005, Zatoń et al. 2005, Barycka 2007, Konietzko-Meier & Wawro 2007, Piechowski & Dzik 2010, Skawina 2010, 2013, Skawina & Dzik 2011, Bodzioch & Kowal-Linka 2012 Skrzycki 2015, Antczak & Bodzioch 2018,<br>Kowalski et al. 2019, Sulej et al. 2021,<br>this study
**Woźniki**	Middle-Late Carnian (Late Triassic);	Plants (macroremains, palynomorphs in bromalites);	No plant fossils	**Trace fossil record:**	Sulej et al. 2011, 2020; Szczygielski & Sulej 2023, this study

SUPPLEMENTARY TABLES

	Krasiejów-Woźniki assemblage	invertebrates (bivalves, ostracods, conchostracans, cyclidans); fish (sharks, actinopterygians, sarcopterygians); tetrapods (temnospondyls, archosauromorphs, therapsids); trace fossils (bromalites, tetrapod tracks, invertebrate traces).		Tetrapod tracks<br>[Chirotheriidae indet. (9, 13, 14),<br>cf. *Atreipus* isp. (12),<br>cf. *Grallator* isp. (?12),<br>cf. *Brachychirotherium* isp. (13, 14),<br>cf. *Apatopus* isp. (9),<br>Tetrapoda track indet. (?)]<br><br>Bromalites, morphotypes M1-M3<br>(for more details see Supp. Tab. 4)<br><br>**Body fossil record:**<br><br>*Nemacanthus* sp. (3)<br>Hybodontiformes indet. (3)<br>Actinopterygii indet. (1)<br>Dipnoa indet. (4)<br>Temnospondyli indet. (?)<br>Plagiosauridae indet. (6)<br>*Cyclotosaurus* sp. (8)<br>*Ozimek* sp. (11)<br>Phytosauria indet. (9)<br>Aetosauria indet. (13)<br>Silesauridae indet. (12)<br>*Woznikella triradiata* (16)<br>*Polonodon woznikensis* (15)	
**Poręba**	Middle-Late Norian (Late Triassic); Poręba -Kocury assemblage	Plants (macroremains, palynomorphs); invertebrates (bivalves, ostracods); fish (sharks, actinopterygians, sarcopterygians); tetrapods (temnospondyls, turtles, archosauromorphs); trace fossils (bromalites, invertebrate traces, bite marks).	**Plants in sediments:**<br><br>cf. *Brachyphyllum* sp.<br>cf. *Pagiophyllum* sp.<br>cf. *Patokaea* sp.<br>*Agathoxylon* cf. *keuperianum*	**Trace fossil record:**<br><br>Bromalites, morphotypes M1-M6<br>(for more details see Supp. Tab. 5)<br><br>Bite marks (isolated bones of Tetrapoda indet.)<br><br>**Body fossil record:**<br><br>Hybodontiformes indet. (3)<br>cf. *Lissodus* sp. (3)<br>Actinopterygii indet. (1)<br>*Ceratodus* sp. (4)<br>*Ptychoceratodus* sp. (4)<br>Coelacanthiformes indet. (5)<br>Temnospondyli indet. (?)<br>*Cyclotosaurus* sp. (8)<br>*Proterochersis porebensis*<br>Archosauromorpha indet. A (?)<br>Archosauromorpha indet. B (?)<br>Aetosauria indet. (13)<br>Silesauridae indet. (12)<br>Theropoda indet. (*Tawa*-like) (18)<br>Neotheropoda indet. (19)<br>Tetrapoda indet. A (?)<br>Tetrapoda indet. B (?)	Sulej et al. 2012, Niedźwiedzki et al. 2014, Pacyna 2014, Bajdek et al. 2019, this study
**Kocury**	Middle-Late Norian (Late Triassic); Poręba -Kocury assemblage	Invertebrates (bivalves); fish (sarcopterygians); tetrapods (turtles, archosauromorphs).	No plant fossils	**Body fossil record:**<br><br>*Termatosaurus albertii* (?)<br>*Velocipes guerichi* (19)<br>*Kocurypelta silvestris* (13)<br>Archosauromorpha indet. (?)<br>Archosauria indet. (?)<br>cf. *Metaceratodus* sp. (4)<br>*Proterochersis* cf. *porebensis* (17)	Czepiński et al. 2021
**Lisowice**	Late Norian-earliest Rhaetian (Late Triassic); Lisowice - Marciszów assemblage	Plants (macroremains, palynomorphs); invertebrates (bivalves, ostracods, conchostracans, insects); fish (sharks, actinopterygians, sarcopterygians); tetrapods (temnospondyls, archosauromorphs, therapsids); trace fossils (bromalites, tetrapod	**Plants in sediments:**<br><br>*Equisetites* sp.<br>cf. *Dioonitocarpidium* sp.<br>*Brachyphyllum* sp.<br>cf. *Pagiophyllum* sp.<br>cf. *Hirmeriella* sp.<br>*Lepidopteris ottonis*<br>cf. *Nilssonia* sp.<br>cf. *Androstrobus* sp.<br>cf. *Beania* sp.<br>*Elatocladus* sp.	**Trace fossil record:**<br><br>Tetrapod tracks<br>[Archosauria track indet. (?),<br>Dicynodontia track indet. (16),<br>Tetrapoda track indet. (?),<br>Theropoda track indet. A (18),<br>Theropoda track indet. B (19),<br>Theropoda track indet. C (25),<br>Theropoda track indet. D (26),<br>Ornihischia track indet. (24)]	Dzik et al. 2008a,b<br>Niedźwiedzki et al. 2011, 2012<br>Wawrzyniak 2010a-d<br>Niedźwiedzki 2013<br>Pieńkowski et al. 2014<br>Pacyna 2014,<br>Zatoń et al. 2015<br>Qvarnström et al. 2019<br>Sulej & Niedźwiedzki 2019,

SUPPLEMENTARY TABLES

		tracks, invertebrate traces, bite marks).	Carpolithes sp.<br>*Peltaspermum rotula*<br>*Podozamites* sp.<br>*Neocalamites* sp.<br>cf. *Dictyophyllum* sp.<br>cf. *Stachyotaxus septentrionalis*<br>cf. *Schmeissneria* sp.<br>*Agathoxylon keuperianum*	Bromalites, morphotypes M1-M8 (for more details see Supp. Tab. 6)<br><br>Bite marks (bones od *Lisowicia*, *Smok*, *Thikia* shells)<br><br>**Body fossil record:**<br><br>*Lisowicia bojani* (16)<br>?Pterosauria indet. (21)<br>?Sharovipterygidae indet. (11)<br>?Ornithischia indet. (24)<br>Theropoda indet. A (18)<br>Theropoda indet. B (19)<br>Theropoda indet. C (25)<br>Crocodylomorpha indet.(22)<br>Thalattosauria indet. (20)<br>Sphenodontia indet. (10)<br>Diapsida indet. (?)<br>*Hallautherium* sp. (23)<br>Cynodontia indet. (15)<br>*Cyclotosaurus* sp. (8)<br>*Gerrothorax* sp. (6)<br>*Smok wawelski* (26)<br>Paleoniscidae indet. (1)<br>Coelacanthiformes indet.(5)<br>Dipnoi indet. (4)<br>*Ptychoceratodus* sp. (4)<br>Rhomphaiodon sp. (3)<br>Hybodont. (3)	Warzyniak, 2023, this study
**Marciszów**	Late Norian-earliest Rhaetian (Late Triassic); Lisowice - Marciszów assemblage	Plants (macroremains, palynomorphs); invertebrates (bivalves, ostracods, conchostracans); fish (sharks, actinopterygians, sarcopterygians); tetrapods (temnospondyls, archosauromorphs, therapsids); trace fossils (bromalites, tetrapod tracks, invertebrate traces, bite marks).	**Plants in sediments:**<br><br>*Equisetites* sp.<br>*Neocalamites* sp.<br>cf. *Neocalamites* sp<br>cf. *Elatocladus* sp.<br>cf. *Pagiophyllum* sp.<br>cf. *Brachyphyllum* sp.<br>*Agathoxylon keuperianum*<br>*Agathoxylon* cf. *keuperianum*<br>Gymnospermae indet.	**Trace fossil record:**<br><br>Tetrapod tracks [?Archosauromorpha track indet. (?), Archosauria track indet. (?), ?Dicynodontia track indet. (16), Tetrapoda track indet. (?)]<br><br>Bromalites, morphotypes M1-M2 (for more details see Supp. Tab. 6)<br><br>Bite marks (bones of dicynodont)<br><br>**Body fossil record:**<br><br>Paleoniscidae indet. (1)<br>Coelacanthiformes indet. (5)<br>Dipnoi indet. (4)<br>Dicynodontia indet. (16)<br>Archosauria indet. (?)<br>*Smok* sp. (26)	Budziszewska-Karwowska et al. 2010<br>Sadlok & Wawrzyniak 2013<br>Niedźwiedzki & Budziszewska-Karwowska 2018<br>Sadlok 2022<br>Wawrzyniak & Filipiak 2023
**Gromadzice-Rzuchów**	Middle-Late Rhaetian (latest Triassic); Gromadzice-Rzuchów assemblage	Plants (macroremains); invertebrates (bivalves, conchostracans); fish (actinopterygians); tetrapods (temnospondyls, archosauromorphs); trace fossils (bromalites, tetrapod tracks, invertebrate traces).	**Plants in sediments:**<br><br>*Lepidopteris ottonis*<br>*Hirmeriella* sp.<br>*Czekanowskia* sp.<br>*Cladophlebis* sp.<br>*Dictyophyllum* sp.<br>*Neocalamites* sp.<br>*Equisetites* sp.<br>*Nilssonia* sp.<br>cf. *Podozamites* sp.<br>cf. *Brachyphyllum* sp.<br>cf. *Stachyotaxus* sp.	**Trace fossil record:**<br><br>Tetrapod tracks [*Grallator* isp. (18), *Anchisauripus* isp. (19), *Kayentapus* isp. (25), *Eubrontes* isp. (26), *Anomoepus* isp. (24), cf. *Tetrasauropus* isp. (27), cf. *Evazoum* isp. (28), cf. *Chirotherium* isp. (28), Tetrapoda track indet. (?)]<br><br>Bromalites, morphotypes M1-M4 (for more details see Supp. Tab. 7)<br><br>**Body fossil record:**<br><br>Paleoniscidae indet. (1)<br>*Cyclotosaurus* sp. (8)	This study
**Hucisko**	Latest Rhaetian-early Hettangian (latest Triassic-earliest	Plants (macroremains, palynomorphs); invertebrates (conchostracans);	**Plants in sediments:**<br><br>*Hirmeriella muensteri*	**Trace fossil record:**	This study

	Jurassic); Sołtyków-Hucisko assemblage	tetrapods (archosauromorphs); trace fossils (bromalites, bite marks).	*Thaumatopteris brauniana*<br>*Czekanowskia* sp.	Bromalites, morphotypes M1-M2 (for more details see Supp. Tab. 8)<br><br>**Body fossil record:**<br><br>Theropoda indet. (?25)<br>Sauropodomorpha indet. (?)	
**Sołtyków**	Latest Rhaetian-early Hettangian (latest Triassic-earliest Jurassic); Sołtyków-Hucisko assemblage	Plants (macroremains, palynomorphs); invertebrates (bivalves, ostracods, conchostracans, insects); fish (sharks, actinopterygians); tetrapods (lepidosauromorphs, turtles, archosauromorphs, therapsids in trace fossil record; archosauromorphs in body fossil record); trace fossils (bromalites, tetrapod tracks, tetrapod burrows, invertebrate traces, bite marks, putative dinosaur nests, eggs, eggshels).	**Plants in sediments:**<br><br>*Hirmeriella muensteri*<br>*Podozamites* cf. *schenkii*<br>*Podozamites* sp.<br>*Swedenborgia* sp.<br>*Schmeissneria microstachys*<br>*Piroconites kuespertii*<br>*Neocalamites lehmannianus*<br>*Odrolepis liassica*<br>*Thaumatopteris brauniana*<br>*Goepertella microloba*<br>*Dictyophyllum* sp.<br>*Todites princeps*<br>*Phlebopteris angustiloba*<br>*Pachypteris lanceolata*<br>*Sagenopteris nilssoniana*<br>*Caytonia* sp.<br>*Otozamites brevifolius*<br>*Pterophyllum* sp.<br>*Paracycas minuta*<br>*Cladophlebis* sp.<br><br>**Plants in bromalites:**<br><br>*Pachypteris papillosa*<br>*Komlopteris distinctiva*<br>*Ptilozamites cycadea*<br>*Nilssonia* sp.<br>cf. *Pterophyllum* sp.<br>Ginkgophyta gen. et sp. indet.<br>cf. *Podozamites* sp.<br>*Aciphyllum triangulatum*<br>*Brachyphyllum* sp.<br>?Pinophyta gen. et sp. indet.<br>cf. *Pseudotorellia* sp.<br>*Desmiophyllum harrisii*	**Trace fossil record:**<br><br>Tetrapod tracks<br>[cf. *Stenonyx* isp. (?),<br>*Grallator* isp. (18),<br>*Anchisauripus* isp. (19),<br>*Kayentapus* isp./*Eubrontes* isp. (25),<br>cf. *Megalosauripus* isp. (29),<br>*Anomoepus* isp. (24),<br>*Delatorrichnus* isp. (31),<br>cf. *Tetrasauropus* isp. (27),<br>cf. *Otozoum* isp. (28),<br>*Parabrontopodus* isp. (30),<br>*Ameghinichnus* isp. (23),<br>*Dicynodontipus* isp. (15),<br>*Rhynchosauroides* isp. (10),<br>Pterosaur track indet. (21),<br>*Batrachopus* isp. (22),<br>Protosuchidae track indet. (22),<br>Turtle track indet. (17)]<br><br>Bromalites, morphotypes M1-M11 (for more details see Supp. Tab. 9)<br><br>Tetrapod burrows (several morphotypes)<br><br>Dinosaur nest structures<br><br>Dinosaur eggs<br><br>Eggshels<br><br>Bite marks (bones of tetrapods)<br><br>**Body fossil record:**<br><br>Paleoniscidae indet. A (1)<br>Paleoniscidae indet. B (1)<br>Theropoda indet. (19/20)<br>Crocodylomorpha indet. (22)<br>Dinosaur embryos (?)	Gierliński et al. 2001, 2004, Pieńkowski 1999, 2004a,b, Ziaja 2006, Niedźwiedzki 2011, Barbacka et al. 2007, 2010, 2022, Qvarnström et al. 2022, this study

Supplementary Table 3: List of synchrotron-scanned (SC) and other studied bromalite specimens from Krasiejów (n=127). Methods used in the research – SEM (scannig electron microscopy), EDS (energy-dispersive X-ray spectroscopy), MTS (microscopic observations of thin sections), ME (microscopic examinations of residual materials from dissolution), GCH (geochemical studies).

Specimen(s)	Shape/Morphotype (M1-M7)	Assigned to	Dimensions MaLxMaWxMiW (mm)	Identified inclusions	Comments
**ZPAL AbIII/3412**	Scroll, M1	*Ptychoceratodus*	47x34.5x25	Unidentified globules.	SC. Surface observations.
**ZPAL AbIII/3401**	Scroll. M1	*Ptychoceratodus*		Semi-articulated fish remains, bivalves.	SC. Studied by Qvarnström et al.[33]
**ZPAL AbIII/3413**	Scroll, M1	*Ptychoceratodus*		Bivalves, ostracods, and many small fragments.	SC. Surface observations.
**ZPAL AbIII/3414**	(Likely) Scroll, ?M1	*Ptychoceratodus*	>51,5x38x27	Plant fragments, small bones.	SC. Surface observations.
**ZPAL AbIII/3415**	Scroll, M1	*Ptychoceratodus*	55x35x22.5	Fish remains.	SC. Surface observations.
**ZPAL AbIII/3416**	Scroll, M1	*Ptychoceratodus*	66x41x30	Fish remains, spiral structures.	SC. Surface observations.
**ZPAL AbIII/3417b**	Big, irregular, M2	*Polonosuchus*		Fish remains, temnospondyl skull fragment, and other bones.	SC. Likely a regurgitalite
**ZPAL AbIII/3417a**	Big, irregular, M2	*Polonosuchus*	76x38x21	Fish remains, temnospondyl skull fragment, small vertebrae and plant cuticles.	SC. Likely a regurgitalite
**ZPAL AbIII/3418**	Oval, M3	Temnospondyl	24x19x15.5	Fish scales.	SC. Surface observations.
**ZPAL AbIII/3419**	Oval, M3	Temnospondyl	35x21.5x16	Beetle elytron and plant fragments.	SC. Surface observations.
**ZPAL AbIII/3420**	Oval, M3	Temnospondyl	32,5x22.5x21	Many small spicules of unknown origin.	SC. Surface observations.
**ZPAL AbIII/3421**	Oval, M3	Temnospondyl	31.5x26x20	Plant cuticles and fish scales.	SC. Surface observations.
**ZPAL AbIII/3422**	Oval, M3	Temnospondyl	34x22x17.5	Abundant fish remains.	SC. Surface observations.
**ZPAL AbIII/3423**	Oval, M3	Temnospondyl	31x21x19	Mineralized “bubbles” and small inclusions.	SC. Surface observations.
**ZPAL AbIII/3424**	Oval, M3	Temnospondyl	46x36.5x28		SC. Surface observations.
**ZPAL AbIII/3425**	Oval, M3	Temnospondyl	34x24.5x22		SC. Surface observations.
**ZPAL AbIII/3426**	Oval, M3	Temnospondyl	32x19.5x17.5	Plant fragments.	SC. Surface observations.
**ZPAL AbIII/3427**	Oval, M3	Temnospondyl	35.5x28.5x22	Ostracod, mineralized cracks and “bubbles”.	SC. Surface observations.
**ZPAL AbIII/3428**	Oval, ?M (?M3)	*Silesaurus?*	>17x32x29	An articulated beetle.	SC. Surface observations.
**ZPAL AbIII/3890**	Small elongated, M7	Fish	-	Small inclusions.	SC. Surface observations.
**ZPAL AbIII/3429**	Elongate, M4	*Silesaurus*?	45x18x13	Remains of beetles (elytra and a pronotum) and fish.	SC. Surface observations.
**ZPAL AbIII/3430**	Elongate, M4	?	4816.5x12.5	Burrows in the bromalite matrix.	SC. Surface observations
**ZPAL AbIII/3431**	Elongate, M4	*Silesaurus*?	26x14x9	Remains of beetles (elytra) and fish.	SC. Surface observations Many small fragments. Surface observations.
**ZPAL AbIII/3432**	Elongate, M4	*Paleorhinus?*	43x21.5x12.5	Fish scales and a structure with denticles.	SC. Surface observations
**ZPAL AbIII/3433**	Elongate, M4	*Paleorhinus*	50.5x23x19	Abundant fish remains.	SC. Surface observations
**ZPAL AbIII/3434**	Elongate, M4	*Stagonolepis?*	>45x24x18	Plant cuticles and arthropod remains including elytra.	SC. Surface observations
**ZPAL AbIII/3435**	Elongate, M4	*Stagonolepis?*	>40x25x21.5	Possible arthropod remains.	SC. Surface observations
**ZPAL AbIII/3436**	Elongate, M4	*Silesaurus*	>19x21.5x17	Abundant arthropod remains including beetle elytra, a possible pectinate antenna, and a mandible element.	SC. Surface observations
**ZPAL AbIII/3437**	Elongate, flat, ?M	?	66,5x20.5x13	Small inclusions.	SC. Surface observations.
**ZPAL AbIII/3438**	Elongate, flat, ?M	*Paleorhinus?*	42x24.5x>14	Abundant fish remains.	SC.
**ZPAL AbIII/3439**	Elongate, flat, ?M	*Silesaurus*?	>33,5x21.5x9	Beetle body, insect appendage, and many small inclusions.	SC. Surface observations.
**ZPAL AbIII/3440**	Elongate, ?M	*Paleorhinus*?	50.5x28x12	Abundant fish remains and other structures.	SC. Surface observations.
**ZPAL AbIII/3502**	Elongate, flat, ?M	?	33x18.5x9	Small inclusions.	SC. Surface observations.

SUPPLEMENTARY TABLES

**ZPAL AbIII/3503**	Elongate, flat, ?M	?	57x25x11.5	Small inclusions.	SC. Surface observations.
**ZPAL AbIII/3504**	Elongate, flat, ?M	*Silesaurus*?	55x24x14	Arthropod remains including beetle elytra and crustacean. One possible bone fragment.	SC. Surface observations.
**ZPAL AbIII/3505**	Elongate, flat, ?M	*Paleorhinus?*	>17x12x6	Abundant fish remains.	SC. Surface observations.
**ZPAL AbIII/3506**	Elongate, flat, ?M	?	>32x17x6	Small inclusions.	Surface observations.
**ZPAL AbIII/3507**	Elongate, flat, ?M	?	40x19.5x13	Small inclusions.	SC. Surface observations.
**ZPAL AbIII/3408**	Elongate, irregular surface, M4	*Silesaurus*	>41x37x26.5	Two semi-articulated beetles, at least 14 elytra, an ostracod, a possible midline fish scale, and a big structure with denticles (possible plant).	SC. Studied by Qvarnström et al.[29]. Not complete.
**ZPAL AbIII/3409**	Elongate, irregular surface, M4	*Silesaurus*	40x19x10.5	A few elytra, fish scales, and many unidentified fragments of insects and/or plants.	SC.Studied by Qvarnström et al.[29]
**ZPAL AbIII/3411**	Elongate, irregular surface, M4	*Silesaurus*	>38.5x20.5x12	A few elytra, swirl-shaped possible insect fragment, and a beetle thorax element.	SC. Studied by Qvarnström et al.[29]
**ZPAL AbIII/3402**	Elongate, irregular surface, M4	*Silesaurus*	54.5x22x19.5	At least 25 elytra including three big ones, two beetle pronotums, two beetle tibiae, and a carabid prosternum.	SC.Studied by Qvarnström et al.[29, 33]
**ZPAL AbIII/3410**	Elongate, irregular surface, M4	*Silesaurus*	31x16x11	Around 10 elytra, a semi-articulated beetle body, a beetle thorax element, and some unidentified beetle fragments.	SC. Studied by Qvarnström et al.[29]
**ZPAL AbIII/3508**	Elongated spiral bromalite, M5	Sarcopterygian fish	29.5x14x12	Various fish remains, elytra, a charophyte gyrogonite, an ostracod, an arthropod appendage and an enigmatic worm-like inclusion of unknown origin.	SC. Surface observations.
**ZPAL AbIII/3509**	Elongated spiral bromalite, M5	Sarcopterygian fish	28.5x13x9	Various fish remains including semi-articulated fins and chunks of scales.	SC. Surface observations.
**ZPAL AbIII/3510**	Small, spiral structure, M6	Fish/Hybodont shark	18.5x8x8	Fish scales, ostracods, tapeworm proglottids.	SC. Surface observations.
**ZPAL AbIII/3511**	Small, spiral structure, M6	Fish/Hybodont shark	18.5x8.5x8	Fish scales, ornamented dermal bone, fish bones, possible seed.	SC. Surface observations.
**ZPAL AbIII/3512**	Small, spiral structure, M6	Hybodont shark?	12	Fish scales, fish bones, ostracod.	SC. Surface observations.
**ZPAL AbIII/3513**	Elongate, little flattened, ?M	Fish?	>28.5x16x12	Several articulated and disarticulated ostracods.	SC. Surface observations.
**ZPAL AbIII/3514**	Elongate, little flattened, ?M	Fish?	38.5x15x11.5	Arthropod cuticle, charophyte gyrogonite, bivalve.	SC. Surface observations.
**ZPAL AbIII/3515**	Small, bullet-shaped	?	12,5x9x7	Small inclusions.	SC. Surface observations.
**ZPAL AbIII/3518**	Small, bullet-shaped, ?M	?	9x6x5.5	Small inclusions.	SC. Surface observations.
**ZPAL AbIII/3517**	Small elongated, tapered ends, ?M	?	19x8.5x6	Small inclusions.	SC. Surface observations.
**ZPAL AbIII/3516**	Small elongated, tapered ends, ?M	?	17.5x8.5x7	Small inclusions.	SC. Surface observations.
**ZPAL AbIII/3519**	Not complete, ?M	?	-	Fish remains	SC. Surface observations.
**ZPAL AbIII/3520**	Not complete, ?M	*Silesaurus?*	-	Well-preserved complete beetles and many isolated beetle remains.	SC. Cracks with infill. Likely a fragment of an elongated bromalite. Studied by Qvarnström et al.[30]
**ZPAL AbIII/3521**	Oval?, ?M	Temnospondyl?	-	Beetle elytra, fish scales, algae.	SC. Cracks, nodules.
**ZPAL AbIII/3522**	Indet, ?M	?	-		SC. Surface observations..
**ZPAL AbIII/3523**	Flat specimen, ?M	?	-	Numerous plant cuticles, mineralised cracks and spherical structures.	SC. Surface observations.
**ZPAL AbIII/3524**	Indet, ?M	?	-		SC. Surface observations.
**ZPAL AbIII/3525**	Indet, ?M	*Silesaurus?*	-	A beetle elytron, flat segmented inclusion (plant or possibly a cestode), a possible small bone, and mineralised spherical structures.	SC. The specimen is in a concretion.
**ZPAL AbIII/3526**	Indet, ?M	?	-	Numerous secondarily mineralised spherical structures.	SC. Surface observations.
**ZPAL AbIII/3527**	Indet, ?M	?	-	Small inclusions.	Surface observations.
**Unregistered**	Elongated, M4	*Paleorhinus*	61x22x19	Numerous fish remains.	Surface observations. SEM, EDS, MTS, ME, GCH. Specimen dissolved.

SUPPLEMENTARY TABLES

**ZPAL AbIII/3891**	Small elongated, M7	Fish	-	Small inclusions.	Surface observations.
**ZPAL AbIII/3892**	Small elongated, M7	Fish	-	Small inclusions.	Surface observations.
**ZPAL AbIII/3893**	Oval, M3	Temnospondyl?	-	Small inclusions, fish scales, bone fragments.	Surface observations.
**ZPAL AbIII/3894**	Oval, M3	Temnospondyl?	-	Small inclusions, fish scales.	Surface observations.
**ZPAL AbIII/3895**	Oval, M3	Temnospondyl?	-	Small inclusions, plant remains, ?bone fragments.	Surface observations.
**ZPAL AbIII/3897**	Oval, M3	Temnospondyl?	-	Small inclusions, fish scales.	Surface observations.
**ZPAL AbIII/3898**	Oval, M3	Temnospondyl?	-	Small inclusions, fish remains.	Surface observations.
**ZPAL AbIII/3899**	Oval, M3	Temnospondyl?	-	Small inclusions, fish remains.	Surface observations.
**ZPAL AbIII/3900**	Oval, M3	Temnospondyl?	-	Small inclusions, fish teeth, palynomorphs.	Surface observations.
**ZPAL AbIII/3901**	Oval, M3	Temnospondyl?	-	Small inclusions, bone fragments, fish scale.	Surface observations.
**ZPAL AbIII/3902**	Oval, M3	Temnospondyl?	-	Small inclusions, fish remains.	Surface observations.
**ZPAL AbIII/3903**	Not complete, ?M	?	-	Small inclusions, fish scales.	Surface observations.
**ZPAL AbIII/3904**	Oval, M3	Temnospondyl?	-	Small inclusions, coccoid structures, fish remains.	Surface observations.
**ZPAL AbIII/3905**	Oval, M3	Temnospondyl?	-	Small inclusions, fish remains.	Surface observations.
**ZPAL AbIII/3906**	Small elongated, M7	Fish	-	Small inclusions, ostracods.	Surface observations.
**ZPAL AbIII/3907**	Small elongated, M7	Fish	-	Small inclusions, ostracods, coccoid structures. .	Surface observations.
**ZPAL AbIII/3908**	Elongated, M4	*Paleorhinus*	-	Numerous fish remains (teeth, scales and tiny bones).	Surface observations.
**ZPAL AbIII/3909**	Large elongated, tapered, M4	*Polonosuchus*	-	Bone fragments, temnospondyl remains, ?aetosaur osteoderm fragments.	Surface observations.
**Unregistered**	Elongated, M4	*Paleorhinus*	-	Numerous fish remains.	Surface observations. Specimen dissolved.
**Unregistered**	Elongated, M4	*Paleorhinus*	51x23x14	Numerous fish remains.	Surface observations. SEM, EDS, MTS, ME, GCH. Specimen dissolved.
**Unregistered**	Small elongated, M7	Fish	<10	Small inclusions, ostracods.	Surface observations.
**Unregistered**	Small elongated, M7	Fish	<10	Small inclusions, ostracods.	Surface observations.
**Unregistered**	Small elongated, M7	Fish	<10	Small inclusions, ostracods.	Surface observations.
**Unregistered**	Small elongated, M7	Fish	<10	Small inclusions, ostracods.	Surface observations.
**Unregistered**	Oval, M3	Temnospondyl?	-	Small inclusions, fish remains.	Surface observations.
**Unregistered**	Oval, M3	Temnospondyl?	-	Small inclusions, fish remains.	Surface observations.
**Unregistered**	Small elongated, M7	Fish	<10	Small inclusions.	Surface observations.
**Unregistered**	Oval, M3	Temnospondyl?	-	Small inclusions, fish scales, bone fragments.	Surface observations. Specimen dissolved.
**Unregistered**	Small elongated, M7	Fish	<10	Small inclusions, fish scales.	Surface observations.
**Unregistered**	Small elongated, M7	Fish	<10	Small inclusions.	Surface observations.
**Unregistered**	Small elongated, M7	Fish	<10	Small inclusions, fish scales.	Surface observations.
**Unregistered**	Small elongated, M7	Fish	<10	Small inclusions.	Surface observations.
**Unregistered**	Small elongated, M7	Fish	<10	Small inclusions, fish remains.	Surface observations.
**Unregistered**	Small elongated, M7	Fish	<10	Small inclusions.	Surface observations.
**Unregistered**	Small elongated, M7	Fish	<10	Small inclusions, fish scale.	Surface observations. SEM, EDS, ME. Specimen dissolved.
**Unregistered**	Oval, M3	Temnospondyl?	-	Small inclusions, fish remains.	Surface observations.
**Unregistered**	Oval, M3	Temnospondyl?	-	Small inclusions, fish scales.	Surface observations.
**Unregistered**	Small elongated, M7	Fish	<10	Small inclusions.	Surface observations.
**Unregistered**	Small elongated, M7	Fish	<10	Small inclusions, fish remains.	Surface observations.
**Unregistered**	Small elongated, M7	Fish	<10	Small inclusions.	Surface observations.
**Unregistered**	Oval, M3	Temnospondyl?	42x34x32	Small inclusions, fish scales, bone fragments.	Surface observations.
**Unregistered**	Oval, M3	Temnospondyl?	37x28x27	Small inclusions, fish scales.	Surface observations.
**Unregistered**	Oval, M3	Temnospondyl?	-	Small inclusions.	Surface observations.
**Unregistered**	Oval, M3	Temnospondyl?	-	Small inclusions, fish scales.	Surface observations.
**Unregistered**	Small elongated, M7	Fish	<10	Small inclusions.	Surface observations.
**Unregistered**	Small elongated, M7	Fish	<10	Small inclusions.	Surface observations.
**Unregistered**	Scroll, small elongated, M6	Hybodont shark	<10	Small inclusions.	Surface observations.
**Unregistered**	Scroll, small elongated, M6	Hybodont shark	<10	Small inclusions.	Surface observations.
**Unregistered**	Small elongated, M7	Fish	<10	Small inclusions.	Surface observations.
**Unregistered**	Small elongated, M7	Fish	<10	Small inclusions.	Surface observations.
**Unregistered**	Small elongated, M7	Fish	<10	Small inclusions, ostracods.	Surface observations.
**Unregistered**	Small elongated, M7	Fish	<10	Small inclusions.	Surface observations.
**Unregistered**	Small elongated, M7	Fish	<10	Small inclusions.	Surface observations.
**Unregistered**	Oval, M3	Temnospondyl?	31x19x21	Small inclusions, bone fragments.	Surface observations. SEM, EDS, ME. Specimen dissolved.
**Unregistered**	Oval, M3	Temnospondyl?	37x29x23	Small inclusions, fish scales.	Surface observations.
**Unregistered**	Small elongated, M7	Fish	<10	Small inclusions.	Surface observations.
**Unregistered**	Small elongated, M7	Fish	<10	Small inclusions, ostracods.	Surface observations.
**Unregistered**	Small elongated, M7	Fish	<10	Small inclusions.	Surface observations.

SUPPLEMENTARY TABLES

**Unregistered**	Scroll, small elongated, M6	Hybodont shark	<10	Small inclusions, fish scales.	Surface observations. SEM, EDS, ME. Specimen dissolved.
**Unregistered**	Small elongated, M7	Fish	<10	Small inclusions.	Surface observations.
**Unregistered**	Small elongated, M7	Fish	<10	Small inclusions.	Surface observations.
**Unregistered**	Small elongated, M7	Fish	<10	Small inclusions.	Surface observations.
**Unregistered**	Scroll, small elongated, M6	Hybodont shark	<10	Small inclusions, fish remains.	Surface observations.

Supplementary Table 4: List of studied bromalite specimens from Woźniki (n=15). Methods used in the research – SEM (scannig electron microscopy), EDS (energy-dispersive X-ray spectroscopy), MTS (microscopic observations of thin sections), ME (microscopic examinations of residual materials from dissolution), GCH (geochemical studies).

Specimen(s)	Shape/Morphotype (M1-M3)	Assigned to	Dimensions MaLxMaW (mm)	Identified inclusions	Comments
**ZPAL V.34/110**	Elongated, massive, M1	Tetrapoda indet./ Temnospondyl?	60x36	Small inclusions, fish remains.	Surface observations.
**ZPAL V.34/111**	Elongated, massive, M1	Tetrapoda indet./ Temnospondyl?	71x38	Small inclusions, fish scales.	Surface observations.
**ZPAL V.34/112**	Elongated, massive, M1	Tetrapoda indet./ Temnospondyl?	65x34	Fish scales, bone fragments.	Surface observations. ME, specimen dissolved.
**ZPAL V.34/113**	Oval, elongated, M2	Tetrapoda indet.	37x18	Small inclusions.	
**ZPAL V.34/114**	Oval, elongated, M2	Tetrapoda indet.	34x21	Fish scales, plant remains, palynomorphs.	Surface observations. ME, specimen dissolved.
**ZPAL V.34/115**	Oval, elongated, M2	Tetrapoda indet.	28x16	Small inclusions.	Surface observations.
**ZPAL V.34/116**	Elongated, spiral, M3	Fish, ?sarcopterygian	25x8	Small inclusions.	Surface observations.
**ZPAL V.34/117**	Elongated, spiral, M3	Fish, ?sarcopterygian	21x6	Small inclusions.	ME, specimen dissolved
**ZPAL V.34/118**	Oval, elongated, M2	Tetrapoda indet.	32x11	Small inclusions.	Surface observations.
**ZPAL V.34/119**	Oval, elongated, M2	Tetrapoda indet.	26x11	Small inclusions.	Surface observations.
**ZPAL V.34/120**	Oval, elongated, M2	Tetrapoda indet.	30x14	Small inclusions.	Surface observations.
**ZPAL V.34/121**	Oval, elongated, M2	Tetrapoda indet.	31x14	Small inclusions.	Surface observations.
**ZPAL V.34/122**	Small, spiral elongated, M3	Hybodont shark	23x6	Small inclusions.	Surface observations.
**ZPAL V.34/123**	Small, spiral elongated, M3	Hybodont shark	28x12	Small inclusions.	Surface observations.
**ZPAL V.34/124**	Small, spiral elongated, M3	Hybodont shark	24x10	Fish remains.	ME, specimen dissolved

Supplementary Table 5: List of studied bromalite specimens from Poręba (n=46). Methods used in the research – SEM (scannig electron microscopy), EDS (energy-dispersive X-ray spectroscopy), MTS (microscopic observations of thin sections), ME (microscopic examinations of residual materials from dissolution), GCH (geochemical studies).

Specimen(s)	Shape/Morphotype (M1-M6) A-D terminology by Bajdek et al. (2019)	Assigned to	Dimensions MaLxMaWxMiW (mm)	Identified inclusions	Comments
**ZPAL V.39/406**	Scroll, slightly tapered along the length, M1(A)	Hybodont shark	31×17×16	Invertebrate eggs.	Surface observations, MTS. Studied by Bajdek et al. (2019): Stereo microscope, thin sections.
**ZPAL V.39/407**	Scroll, slightly tapered along the length, M1(A)	Hybodont shark	20×10×8.5	Abundant fish scales.	Surface observations. Studied by Bajdek et al. (2019): Stereo microscope.
**ZPAL V.39/408**	Cylindrical, tapered along the length, M2(B)	Turtle	53×17×17	Strongy acid-etched bones including probable fish scales; plant remains.	Surface observations, MTS. Studied by Bajdek et al. (2019): Stereo microscope, thin sections.
**ZPAL V.39/409**	Cylindrical, one extremity rounded, M2(B)	Turtle	33×19×17	An poorly-identifible remain possibly representing a fish bone.	Surface observations, MTS. Studied by Bajdek et al. (2019): Stereo microscope, thin sections.
**ZPAL V.39/410**	Cylindrical, slightly tapered along the length, M2(B)	Turtle	18×11×9	Plant remains; invertebrate eggs.	Surface observations, SEM, EDS, MTS. Studied by Bajdek et al. (2019): Stereo microscope, thin sections, SEM/EDS.
**ZPAL V.39/411**	Cylindrical, slightly flattened, one side irregular, M3(C)	Theropod	30×20×15	Abundant poorly acid-etched fish scales and rare bone fragments.	Surface observations. Studied by Bajdek et al. (2019): Stereo microscope.
**ZPAL V.39/412**	Oval, slightly flattened, one side irregular, M3(C)	Theropod	47.5×30.5×24	Abundant poorly acid-etched fish scales and bones.	Surface observations. Studied by Bajdek et al. (2019): Stereo microscope.
**ZPAL V.39/413**	Tear-shaped, surface slightly irregular, M4(D)	?Dicynodont, ?aetosaur	-	Plant remains, invertebrate eggs, possible cyanobacteria.	Surface observations, SEM, EDS, MTS. Studied by Bajdek et al. (2019): Stereo microscope, thin sections, SEM/EDS.
**ZPAL V.39/372 COP01**	Scroll, large, tapered along the length, M6 (morphotype 2 in Zatoń et al. 2015)	Sarcopterygian fish	37×12	Small inclusions.	Surface observations, SEM, EDS, MTS, GCH. Studied by Zatoń et al. (2015): transmitted-light microscopy, SEM, palynology, TOC and TS.
**ZPAL V.39/372 COP16**	Large, elongated, tapered along the length, M3 (morphotype 2 in Zatoń et al. 2015)	Theropod	142×49	Plant remains, arthropod cuticle, bone fragments.	Surface observations, SEM, EDS, MTS, GCH. Studied by Zatoń et al. (2015): transmitted-light microscopy, SEM, palynology, inorganic geochemistry, XRD, TOC and TS, biomarkers.
**ZPAL V.39/372 COP20**	Cylindrical, tapered along the length, M2	Turtle	45x27	Plant remains, fish remains.	Surface observations.
**ZPAL V.39/372 COP21**	Oval, M4 (D)	?Dicynodont, ?aetosaurs	-	Small inclusions. Plant remains.	Surface observations.
**ZPAL V.39/372 COP22**	Small elongated, M5	Fish	MaW<10	Small inclusions.	Surface observations.
**ZPAL V.39/372 COP23**	Large elongated, M3	Theropod	47x31	Plant remains, fish scales and bone fragments.	Surface observations, SEM, EDS, MTS, ME.
**ZPAL V.39/372 COP24**	Small elongated, M5	Fish	MaW<10	Small inclusions.	Surface observations.
**ZPAL V.39/372 COP25**	Small elongated, M5	Fish	MaW<10	Small inclusions.	Surface observations.
**ZPAL V.39/372 COP26**	Large elongated, M3	Theropod	127×43	Fish scales and bone fragments.	Surface observations.
**ZPAL V.39/372 COP27**	Small elongated, M5	Fish	MaW<10	Small inclusions.	Surface observations.
**ZPAL V.39/372 COP28**	Large elongated, M3	Theropods	106×39	Fish scales and bone fragments.	Surface observations.
**ZPAL V.39/372 COP29**	Oval, M4(D)	?Dicynodont, ?aetosaurs	-	Plant remains.	Surface observations.

SUPPLEMENTARY TABLES

**ZPAL V.39/372 COP30**	Small elongated, M5	Fish	MaW<10	Small inclusions.	Surface observations.
**ZPAL V.39/372 COP31**	Small elongated, M5	Fish	MaW<10	Small inclusions.	Surface observations.
**ZPAL V.39/372 COP32**	Oval, M4(D)	?Dicynodont, ?aetosaur	-	Plant remains.	Surface observations.
**ZPAL V.39/372 COP33**	Oval, M4(D)	?Dicynodont, ?aetosaur	-	Plant remains.	Surface observations.
**ZPAL V.39/372 COP34**	Oval, M4(D)	?Dicynodont, ?aetosaur	-	Plant remains.	Surface observations.
**ZPAL V.39/372 COP35**	Scroll, slightly tapered along the length, M1(A)	Hybodont shark	26×12	Abundant fish scales.	Surface observations.
**ZPAL V.39/372 COP36**	Large elongated, M3	Theropod	91×23	Fish scales and bone fragments.	Surface observations.
**ZPAL V.39/372 COP37**	Large elongated, M3	Theropod	101×29	Fish scales and bone fragments.	Surface observations.
**ZPAL V.39/372 COP38**	Large elongated, M3	Theropod	69×21	Fish scales and bone fragments.	Surface observations.
**ZPAL V.39/372 COP39**	Small elongated, M5	Fish	MaW<10	Small inclusions.	Surface observations.
**ZPAL V.39/372 COP40**	Small elongated, M5	Fish	MaW<10	Small inclusions.	Surface observations.
**ZPAL V.39/372 COP41**	Small elongated, M5	Fish	MaW<10	Small inclusions.	Surface observations.
**ZPAL V.39/372 COP42**	Large elongated, M3	Theropod	98×26	Fish scales and bone fragments.	Surface observations.
**ZPAL V.39/372 COP43**	Large elongated, M3	Theropod	76×23	Fish scales and bone fragments.	Surface observations.
**ZPAL V.39/372 COP44**	Large elongated, M3	Theropod	81×28	Fish scales and bone fragments.	Surface observations.
**ZPAL V.39/372 COP45**	Large elongated, M3	Theropod	93×21	Fish scales and bone fragments.	Surface observations.
**ZPAL V.39/372 COP46**	Oval, M4(D)	?Dicynodont, ?aetosaur	-	Plant remains.	Surface observations.
**ZPAL V.39/372 COP47**	Oval, M4(D)	?Dicynodont, ?aetosaur	-	Plant remains.	Surface observations.
**ZPAL V.39/372 COP48**	Cylindrical, M4(D)	?Dicynodont, ?aetosaur	-	Plant remains.	Surface observations.
**ZPAL V.39/372 COP49**	Large elongated, M3	Theropod	72×22	Fish scales and bone fragments.	Surface observations.
**ZPAL V.39/372 COP50**	Cylindrical, tapered along the length, M2	Turtle	57x28	Plant remains, fish remains.	Surface observations.
**ZPAL V.39/372 COP51**	Small elongated, M5	Fish	MaW<10	Small inclusions.	Surface observations.
**ZPAL V.39/372 COP52**	Small elongated, M5	Fish	MaW<10	Small inclusions.	Surface observations.
**ZPAL V.39/372 COP53**	Small elongated, M5	Fish	MaW<10	Small inclusions.	Surface observations.
**ZPAL V.39/372 COP54**	Small elongated, M5	Fish	MaW<10	Small inclusions.	Surface observations.
**ZPAL V.39/372 COP55**	Small elongated, M5	Fish	MaW<10	Small inclusions.	Surface observations.

Supplementary Table 6: List of scanned (sc) and studied bromalite specimens from Lisowice (n=231) and Marciszów (n=5). Methods used in the research – SEM (scannig electron microscopy), EDS (energy-dispersive X-ray spectroscopy), MTS (microscopic observations of thin sections), ME (microscopic examinations of residual materials from dissolution), GCH (geochemical studies).

Specimen(s)	Shape/Morpho type (M1-M8 – Lisowice; M1-M2 Marciszów)	Assigned to	Dimensions (mm)	Identified inclusions	Comments
**ZPAL V.33/340 (A, B fragments)**	Big, elongate	*Smok*	125	Bone fragments, microbial structures.	Studied by Qvarnström et al. [31]. Surface observations. SEM, EDS, MTS, ME, GCH. Specimen dissolved.
**ZPAL V.33/341**	Big, elongate	*Smok*	87x31	Various bones (including a temnospondyl one), a serrated tooth, pyritized microbial colonies, gas bubbles.	SC. Studied by Qvarnström et al. [31]. Surface observations.
**ZPAL V.33/342 (C, D fragments)**	Big, elongate, M1	*Smok*	94	Bone fragments, microbial structures.	Studied by Qvarnström et al. [31] and Zatoń et al. (2015). Surface observations.
**ZPAL V.33/343**	Big, elongate, M1	*Smok*	130	Bone fragments. Strongly pyritized.	Studied by Qvarnström et al. [31]
**ZPAL V.33/344**	Big, elongate, M1	*Smok*	92x33	Various bones, serrated teeth (a big and a small one), pyritized microbial colonies, gas bubbles.	SC. Studied by Qvarnström et al. [31]. Surface observations.
**ZPAL V.33/345**	Big, elongate, M1	*Smok*	91x29	Various bones (including ribs and a juvenile dicynodont. Pyritized microbial colonies, gas bubbles.	SC. Studied by Qvarnström et al. [31]. Surface observations.
**ZPAL V.33/346**	Big, elongate, M1	*Smok*	116x39	Various bones (some very dicynodont-like), microbially-induced tunnelling and surface attacks.	Studied by Qvarnström et al. [31]. Surface observations. SEM, EDS, MTS, ME, GCH. Specimen dissolved.
**ZPAL V.33/600**	Big, elongate, M1	*Smok*	118x37	An enamel-stripped tooth of *S. wawelski*, fish remains, bone fragments, microbial structures.	Studied by Qvarnström et al. [31]. Surface observations. SEM, EDS, MTS, ME, GCH. Specimen dissolved.
**ZPAL V.33/604**	Big, elongate, M1	*Smok*	176x52	Bone fragments, microbial structures.	Studied by Qvarnström et al. [31]. Surface observations. SEM, EDS, MTS, ME, GCH. Specimen dissolved.
**ZPAL V.33/1890**	Big, elongate, M1	*Smok*	250x68	Bone fragments, microbial structures.	Studied by Qvarnström et al. [31]. Surface observations. SEM, EDS, MTS, ME, GCH. Specimen dissolved.
**ZPAL V.33/704**	Irregular	*Smok* (regurgitalite)	-	Big bone fragments with sharp margins.	Studied by Qvarnström et al. [31]. Surface observations.
**ZPAL V.33/1306**	Irregular	*Smok* (regurgitalite)	-	Gnawed bone fragments and phosphatized soft tissue.	Studied by Qvarnström et al. [31]. Surface observations.
**ZPAL V.33/1270 COP02 (fragment)**	?Big, elongate, M1	*Smok*	(not complete specimen)	Fish scales and phosphatized tissue.	Studied by Zatoń et al. (2015): palynology. Specimen dissolved.
**ZPAL V.33/1270 COP03 (fragment)**	Elongate, M2	Theropod	(not complete specimen)	Small inclusions.	Studied by Zatoń et al. (2015): SEM, TOC and TS. Specimen dissolved.
**ZPAL V.33/1270 COP04**	?Spiral, M3	Lungfish/ Coelacanth	41x17	Abundant semi-articulates fish remains including tooth plates, scales, and possible soft tissue. Bone fragments.	SC. Studied by Zatoń et al. (2015): transmitted-light microscopy, SEM, TEM, STXM, inorganic geochemistry, XRD, TOC and TS.
**ZPAL V.33/1270 COP05**	Elongate, M2	?Theropod	34x16	Plant remains, arthropod cuticle, degraded bone fragments. Mineralized spheres and cracks.	SC. Studied by Zatoń et al. (2015): transmitted-light microscopy, SEM, palynology, inorganic geochemistry, XRD, TOC and TS, biomarkers.
**ZPAL V.33/1270 COP06**	?Amphipolar, M4	Lungfish/ Coelacanth	87x24	Abundant fish remains including scales and fin lepidotrichia. Rare plant remains, ostracode carapace, bone fragments.	SC. Studied by Zatoń et al. (2015): transmitted-light microscopy, palynology, inorganic geochemistry, XRD, TOC and TS, biomarkers.
**ZPAL V.33/1270 COP07 (fragment)**	Elongate, M2	Theropod	(not complete specimen)	Plant remains, degraded bone fragments.	Studied by Zatoń et al. (2015): transmitted-light microscopy, palynology, inorganic geochemistry, XRD, TOC and TS.
**ZPAL V.33/1270 COP08 (fragment)**	?Elongate, M1 or M2	?Theropod/ ?*Smok* 51x42	(not complete specimen)	Many plant remains. Bone fragments.	SC. Studied by Zatoń et al. (2015): transmitted-light

SUPPLEMENTARY TABLES

					microscopy, SEM, palynology, inorganic geochemistry, XRD.<br><br>Specimen in fragments. Part of the specimen was dissolved.
**ZPAL V.33/1270 COP09**	Elongate, M2	Theropod	76x41	Scarce fish scales? Plant remains, bone fragments. Cracks.	Studied by Zatoń et al. (2015): SEM, palynology, inorganic geochemistry, XRD, biomarkers.
**ZPAL V.33/1270 COP10**	Elongated, M4	Ornithischian-like dinosaur	81x22	An ostracod and small inclusions. Plant remains, arthropod cuticle, degraded bone fragments. Mineralized cracks.	Studied by Zatoń et al. (2015): transmitted-light microscopy, SEM, palynology, inorganic geochemistry, XRD. Small part was scanned.
**ZPAL V.33/1270 COP11**	Irregular , M5	? Theropod	56x37	Poorly preserved ?bone fragments. Phosphatic areas with carbon-rich areas.	Pyritized. Studied by Zatoń et al. (2015): transmitted-light microscopy. SEM/EDS.
**ZPAL V.33/1270 COP12**	Elongate, bulbous, M4	Ornithischian-like dinosaur	107x53	Large plant fragments, small bone fragments.	SC. Studied by Zatoń et al. (2015): transmitted-light microscopy. Specimen in several fragments.
**ZPAL V.33/1270 COP013**				Plant remains, degraded bone fragments.	Studied by Zatoń et al. (2015): transmitted-light microscopy.
**ZPAL V.33/1270 COP14**	Elongate, M2	? Theropod	72x26	Bone fragments. Fish scales, plant cuticles.	SC. Studied by Zatoń et al. (2015): transmitted-light microscopy, SEM, TOC and TS, biomarkers.
**ZPAL V.33/1270 COP015**	Irregular/oval; M7	?*Lisowicia*	39x26	Plant remains.	Pyritized. Studied by Zatoń et al. (2015): transmitted-light microscopy.
**ZPAL V.33/1270 COP18**	Amphipolar, M4	Lungfish	29	Abundant fish remains including scales, large bones and tooth plates. Rare plant remains, bone fragments.	Studied by Zatoń et al. (2015): transmitted-light microscopy, SEM, palynology, inorganic geochemistry, XRD, biomarkers.
**ZPAL V.33/1270 COP019 (fragment)**	Elongated, M2	Theropod	(not complete specimen)	Plant remains. Fish scales.	Studied by Zatoń et al. (2015): transmitted-light microscopy. Specimen dissolved.
**ZPAL V.33/1342a**	Fragmentary, ?M	?	-	Abundant plant fragments.	Surface observations.
**ZPAL V.33/1342b**	Fragmentary, ?M	?	-	Abundant plant fragments.	Surface observations.
**ZPAL V.33/1343 (Lisowice-no1)**	Elongate, M4	Ornithischian dinosaur	-	Plant fragments, thorns.	SC. Surface observations.
**ZPAL V.33/1344 (Lisowice-no2)**	Spiral. Amphipolar?	Lungfish?/ Coelacanth?	-	Abundant fish remains. Articulated fins and numerous scales.	Spiral
**ZPAL V.33/1345**	Elongate, M2	Theropod	-	Bone fragments.	Surface observations.
**ZPAL V.33/1346**	Elongate, ?M	-	Ca 10 mm in diameter	Two invertebrate burrows but no food inclusions.	Surface observations.
**ZPAL V.33/1347**	Oval to elongate, M4	Ornithischiandi nosaur	Ca 20x15 mm	Abundant small inclusions, perhaps representing very fragmented plant remains.	Surface observations.
**ZPAL V.33/1348**	Heteropolar?, M8	Hybodont	Ca 4 mm in diameter	Tiny spherical voids and small unidentified inclusions.	Surface observations.
**ZPAL V.33/1037 Lisowice A**	Irregular/oval, M7	*Lisowicia*	-	Plant cuticles.	Surface observations.
**ZPAL V.33/1038**	Irregular/oval, M7	*Lisowicia*	-	Plant cuticles.	Surface observations.
**ZPAL V.33/1039**	Irregular/oval, M7	*Lisowicia*	-	Plant cuticles.	Surface observations.
**ZPAL V.33/1040**	Irregular/oval, M7	*Lisowicia*	-	Plant cuticles.	Surface observations.
**ZPAL V.33/1041**	Irregular/oval, M7	*Lisowicia*	-	Plant cuticles.	Surface observations.
**ZPAL V.33/1101**	Irregular/oval, M7	*Lisowicia*	-	Plant cuticles, small inlusions, clasts.	Surface observations, SEM, EDS, MTS, MT, GCH, specimen dissolved. Studied by Bajdek et al. (2014).
**ZPAL V.33/1102**	Irregular/oval, M7	*Lisowicia*	-	Plant cuticles, small inlusions, clasts.	Surface observations, SEM, EDS, MTS, MT, GCH, specimen dissolved. Studied by Bajdek et al. (2014).
**ZPAL V.33/1103**	Irregular/oval, M7	*Lisowicia*	-	Plant cuticles, small inlusions, clasts.	Surface observations, SEM, EDS, MTS, MT, GCH, specimen dissolved. Studied by Bajdek et al. (2014).
**ZPAL V.33/1104**	Irregular/oval, M7	*Lisowicia*	-	Plant cuticles, small inlusions, clasts.	Surface observations, SEM, EDS, MTS, MT, GCH, specimen dissolved. Studied by Bajdek et al. (2014).
**ZPAL V.33/1105**	Irregular/oval, M7	*Lisowicia*	-	Plant cuticles, small inlusions, clasts.	Surface observations, SEM, EDS, MTS, MT, GCH, specimen

SUPPLEMENTARY TABLES

					dissolved. Studied by Bajdek et al. (2014).
**ZPAL V.33/1106**	Irregular/oval, M7	*Lisowicia*	-	Plant cuticles, small inlusions, clasts.	Surface observations, SEM, EDS, MTS, MT, GCH, specimen dissolved. Studied by Bajdek et al. (2014).
**ZPAL V.33/1107 (Bromalite 1)**	Irregular/oval, M7	*Lisowicia*	-	Plant cuticles, small inlusions, clasts. Plant material studied in details.	Surface observations, SEM, EDS, MTS, MT, GCH, specimen dissolved. Studied by Bajdek et al. (2014).
**ZPAL V.33/1108 (Bromalite 2)**	Irregular/oval, M7	*Lisowicia*	-	Plant cuticles, small inlusions, clasts. Plant material studied in details.	Surface observations, SEM, EDS, MTS, MT, GCH, specimen dissolved. Studied by Bajdek et al. (2014).
**ZPAL V.33/1109 (Bromalite 3)**	Irregular/oval, M7	*Lisowicia*	-	Plant cuticles, small inlusions, clasts. Plant material studied in details.	Surface observations, SEM, EDS, MTS, MT, GCH, specimen dissolved. Studied by Bajdek et al. (2014).
**ZPAL V.33/1110**	Irregular/oval, M7	*Lisowicia*	-	Plant cuticles, small inlusions, clasts.	Surface observations, SEM, EDS, MTS, MT, GCH, specimen dissolved. Studied by Bajdek et al. (2014).
**ZPAL V.33/1111**	Irregular/oval, M7	*Lisowicia*	-	Plant cuticles.	Surface observations, SEM, EDS, MTS, MT, GCH, specimen dissolved. Studied by Bajdek et al. (2014).
**ZPAL V.33/1112**	Irregular/oval, M7	*Lisowicia*	-	Plant cuticles.	Surface observations.
**ZPAL V.33/1113**	Irregular/oval, M7	*Lisowicia*	-	Plant cuticles.	Surface observations.
**ZPAL V.33/1114**	Irregular/oval, M7	*Lisowicia*	-	Plant cuticles.	Surface observations.
**ZPAL V.33/1115**	Irregular/oval, M7	*Lisowicia*	-	Plant cuticles.	Surface observations.
**ZPAL V.33/1116**	Irregular/oval, M7	*Lisowicia*	-	Plant cuticles.	Surface observations.
**ZPAL V.33/1270 COP20**	Irregular/oval, M7	*Lisowicia*	-	Plant cuticles	Surface observations.
**ZPAL V.33/1270 COP21**	Irregular/oval, M7	*Lisowicia*	-	Plant cuticles.	Surface observations.
**ZPAL V.33/1270 COP22**	Irregular/oval, M7	*Lisowicia*	-	Plant cuticles.	Surface observations.
**ZPAL V.33/1270 COP23**	Irregular/oval, M7	*Lisowicia*	-	Plant cuticles.	Surface observations.
**ZPAL V.33/1270 COP24**	Irregular/oval, M7	*Lisowicia*	-	Plant cuticles.	Surface observations.
**ZPAL V.33/1270 COP25**	Irregular/oval, M7	*Lisowicia*	-	Plant cuticles.	Surface observations.
**ZPAL V.33/1270 COP26**	Irregular/oval, M7	*Lisowicia*	-	Plant cuticles.	Surface observations.
**ZPAL V.33/1270 COP27**	Irregular/oval, M7	*Lisowicia*	-	Plant cuticles.	Surface observations.
**ZPAL V.33/1270 COP28**	Irregular/oval, M7	*Lisowicia*	-	Plant cuticles.	Surface observations.
**ZPAL V.33/1270 COP29**	Irregular/oval, M7	*Lisowicia*	-	Plant cuticles.	Surface observations.
**ZPAL V.33/1270 COP30**	Irregular/oval, M7	*Lisowicia*	-	Plant cuticles.	Surface observations.
**ZPAL V.33/1270 COP31**	Irregular/oval, M7	*Lisowicia*	-	Plant cuticles.	Surface observations.
**ZPAL V.33/1270 COP32**	Irregular/oval, M7	*Lisowicia*	-	Plant cuticles.	Surface observations.
**ZPAL V.33/1270 COP33**	Irregular/oval, M7	*Lisowicia*	-	Plant cuticles.	Surface observations.
**ZPAL V.33/1270 COP34**	Irregular/oval, M7	*Lisowicia*	-	Plant cuticles.	Surface observations.
**ZPAL V.33/1270 COP35**	Irregular/oval, M7	*Lisowicia*	-	Plant cuticles.	Surface observations.
**ZPAL V.33/1270 COP37**	Irregular/oval, M7	*Lisowicia*	-	Plant cuticles.	Surface observations.
**ZPAL V.33/1270 COP38**	Irregular/oval, M7	*Lisowicia*	-	Plant cuticles.	Surface observations.
**ZPAL V.33/1270 COP39**	Irregular/oval, M7	*Lisowicia*	-	Plant cuticles.	Surface observations.
**ZPAL V.33/1270 COP40**	Irregular/oval, M7	*Lisowicia*	-	Plant cuticles.	Surface observations.
**ZPAL V.33/1270 COP41**	Irregular/oval, M7	*Lisowicia*	-	Plant cuticles.	Surface observations.
**ZPAL V.33/1270 COP42**	Irregular/oval, M7	*Lisowicia*	-	Plant cuticles.	Surface observations.

SUPPLEMENTARY TABLES

**ZPAL V.33/1270 COP43**	Irregular/oval, M7	*Lisowicia*	-	Plant cuticles.	Surface observations.
**ZPAL V.33/1270 COP44**	Irregular/oval, M7	*Lisowicia*	-	Plant cuticles.	Surface observations.
**ZPAL V.33/1270 COP45**	Irregular/oval, M7	*Lisowicia*	-	Plant cuticles.	Surface observations.
**ZPAL V.33/1270 COP46**	Irregular/oval, M7	*Lisowicia*	-	Plant cuticles.	Surface observations.
**ZPAL V.33/1270 COP47**	Irregular/oval, M7	*Lisowicia*	-	Plant cuticles.	Surface observations.
**ZPAL V.33/1270 COP48**	Irregular/oval, M7	*Lisowicia*	-	Plant cuticles.	Surface observations.
**ZPAL V.33/1270 COP49**	Oval, M8	Temnospondyl	59x46	Fish remains. Small inclusions. Plant cuticles.	Surface observations.
**ZPAL V.33/1270 COP50**	Oval, M8	Temnospondyl	37x25	Small inclusions. Plant cuticles.	Surface observations.
**ZPAL V.33/1270 COP51**	Oval, M8	Temnospondyl	45x39	Fish remains. Small inclusions. Plant cuticles.	Surface observations.
**ZPAL V.33/1270 COP52**	Elongate/Irregular surface, M2	Theropod	36x10	Fish remains, plant cuticules.	Surface observations.
**ZPAL V.33/1270 COP53**	Elongate/Irregular surface, M2	Theropod	47x12	Fish remains, bone fragments, plant cuticules.	Surface observations.
**ZPAL V.33/1270 COP54**	Elongate, M4	Ornithischian-like dinosaur	53x17	Plant fragments.	Surface observations.
**ZPAL V.33/1270 COP55**	Elongate, M4	Ornithischian-like dinosaur	62x15	Plant fragments.	Surface observations.
**ZPAL Mar 1 (Marciszów)**	Irregular/oval (M1)	*Lisowicia*	-	Plant cuticles.	Surface observations.
**ZPAL Mar 2 (Marciszów)**	Irregular/oval (M1)	*Lisowicia*	-	Plant cutiules.	Surface observations.
**ZPAL Mar 3 (Marciszów)**	Irregular/elongated (M2)	*Lisowicia*	-	Plant cuticles.	Surface observations.
**ZPAL Mar 4 (Marciszów)**	Irregular/oval (M2)	*Lisowicia*	-	Plant cuticles.	Surface observations.
**ZPAL Mar 5 (Marciszów)**	Irregular/oval (M2)	*Lisowicia*	-	Plant cuticles.	Surface observations.

Supplementary Table 7: List of studied bromalite specimens from Gromadzice-Rzuchów (n=12). Methods used in the research – SEM (scannig electron microscopy), EDS (energy-dispersive X-ray spectroscopy), MTS (microscopic observations of thin sections), ME (microscopic examinations of residual materials from dissolution), GCH (geochemical studies).

Specimen(s)	Shape/Morphotype (M1-M4)	Assigned to	Dimensions MaLxMaWxMiW(mm)	Identified inclusions	Comments
**Muz PGI OS-221/ GR/Cop 1**	Oval, large bromalite M1	Sauropodomorph	-	Plant remains.	Surface observations. Sideritic bromalite.
**Muz PGI OS-221/ GR/Cop 2**	Oval, large bromalite M1	Sauropodomorph	-	Plant remains.	Surface observations. Sideritic bromalite.
**Muz PGI OS-221/ GR/Cop 3**	Oval, large bromalite M1	Sauropodomorph	-	Plant remains.	Surface observations. Sideritic bromalite.
**Muz PGI OS-221/ GR/Cop 4**	Oval, large bromalite M1	Sauropodomorph	-	?	Surface observations. Sideritic bromalite.
**Muz PGI OS-221/ GR/Cop 5**	Irregular, large bromalite M2	Sauropodomorph	-	Plant remains.	Surface observations. Organic-rich specimen.
**Muz PGI OS-221/ GR/Cop 6**	Elongated, small, M3	Theropod	38×19×17	Fish remains.	Surface observations. Phosphatic bromalite.
**Muz PGI OS-221/ GR/Cop 7**	Elongated, small, M3	Theropod	46×18×16	?	Surface observations. Phosphatic bromalite.
**Muz PGI OS-221/ GR/Cop 8**	Elongated, small, M3	Theropod	60×26×35	Fish remains.	Surface observations. Phosphatic bromalite.
**Muz PGI OS-221/ GR/Cop 9**	Elongated, small, M3	Theropod	83×29×35	?	Surface observations. Phosphatic bromalite.
**Muz PGI OS-221/ GR/Cop 10**	Elongated, small, M3	Theropod	67×28×35	Fish remains.	Surface observations. Phosphatic bromalite.
**Muz PGI OS-221/ GR/Cop 11**	Elongated, large, M4	Theropod	112×43×37	Bone fragments.	Surface observations, SEM, EDS, MTS, ME, GCH. Phosphatic bromalite.
**Muz PGI OS-221/ GR/Cop 12**	Elongated, large, M4	Theropod	141×56×47	?Bone remains.	Surface observations. Sideritic bromalite.

Supplementary Table 8: List of studied bromalite specimens from Hucisko (n=16). Methods used in the research – SEM (scannig electron microscopy), EDS (energy-dispersive X-ray spectroscopy), MTS (microscopic observations of thin sections), ME (microscopic examinations of residual materials from dissolution), GCH (geochemical studies).

Specimen(s)	Shape/Morphotype (M1-M2)	Assigned to	Dimensions MaLxMaWxMiW (mm)	Identified inclusions	Comments
**Muz PGI OS-221/HU/Cop1**	Elongated, small, M1	Theropod	23×6×4	?	Surface observations. Sideritic bromalite.
**Muz PGI OS-221/HU/Cop2**	Elongated, small, M1	Theropod	33×10×11	Fish remains, bone fragments.	Surface observations, SEM, EDS, MTS. Phosphatic bromalite.
**Muz PGI OS-221/HU/Cop3**	Elongated, small, M1	Theropod	26×9×8	Fish remains.	Surface observations. Phosphatic bromalite.
**Muz PGI OS-221/HU/Cop4**	Elongated, small, M1	Theropod	23×11×8	?	Surface observations. Sideritic bromalite.
**Muz PGI OS-221/HU/Cop5**	Elongated, small, M1	Theropod	26×11×9	?	Surface observations. Sideritic bromalite.
**Muz PGI OS-221/HU/Cop6**	Elongated, small, M1	Theropod	24×14×8	?	Surface observations. Sideritic bromalite.
**Muz PGI OS-221/HU/Cop7**	Elongated, large, M2	Theropod	83×50×34	?	Surface observations. Sideritic bromalite.
**Muz PGI OS-221/HU/Cop8**	Elongated, large, M2	Theropod	71×27×35	?	Surface observations. Sideritic bromalite.
**Muz PGI OS-221/HU/Cop9**	Elongated, large, M2	Theropod	91×34×31	Bone remains. Plant remains.	Surface observations. Phosphatic bromalite.
**Muz PGI OS-221/HU/Cop10**	Elongated, large, M2	Theropod	98×31×28	? Bone fragments.	Sideritic bromalite
**Muz PGI OS-221/HU/Cop11**	Elongated, large, M2	Theropod	78×29×19	Bone remains.	Surface observations, SEM, EDS, MTS. Phosphatic bromalite.
**Muz PGI OS-221/HU/Cop12**	Elongated, large, M2	Theropod	97×37×32	?	Sideritic bromalite
**Muz PGI OS-221/HU/Cop13**	Elongated, large, M2	Theropod	92×35×32	? Bone fragments.	Sideritic bromalite, SEM, EDS, GCH.
**Muz PGI OS-221/HU/Cop14**	Elongated, large, M2	Theropod	87×29×22	?	Sideritic bromalite
**Muz PGI OS-221/HU/Cop15**	Elongated, large, M2	Theropod	112×51×33	Fish remains.	Surface observations, SEM, EDS, MTS, GCH. Phosphatic bromalite.
**Muz PGI OS-221/HU/Cop16**	Elongated, large, M2	Theropod	140×37×31	Fish remains. Bone remains. Plant remains.	Surface observations. Phosphatic bromalite.

Supplementary Table 9: List of studied bromalite specimens from Sołtyków (n=148). Methods used in the research – SEM (scannig electron microscopy), EDS (energy-dispersive X-ray spectroscopy), MTS (microscopic observations of thin sections), ME (microscopic examinations of residual materials from dissolution), GCH (geochemical studies).

Specimen(s)	Shape/Morphotype (M1-M11)	Assigned to	Dimensions MaLxMaWxMiW (mm)	Identified inclusions	Comments
**Numerous specimens Muz PGI OS-221/298 (1-100); (100 specimens)**	Small elongated bromalites, straight or curved, segmented, M1, M2	Fish (small to medium actinopterygian).	<15 mm in diameter TL = 7-50 mm	Small inclusions. ?Plant and other organic remains, ?ostracods.	Surface observations. Completely or partially sideritised or pyritised. A few specimens studied by methods SEM, EDS, ME, GCH.
**Muz PGI OS-221/299**	Big, elongated bromalite, M3	Theropod (large-sized).	330×112×95	Bone remains. Numerous small bone fragments. Plant remains (cuticules and palynomorphs).	Partially sideritic bromalite. Studied by methods SEM, EDS. Specimen dissolved.
**Muz PGI OS-221/300**	Big, elongated bromalite, M3	Theropod (large-sized).	272×117×102	Large bone, observation based on broken surface.	Surface observations. Sideritic bromalite. Studied by methods SEM, EDS.
**Muz PGI OS-221/306**	Big, elongated bromalite, M3	Theropod (large-sized).	291×113×89	Bones (poorly preserved).	Surface observations. CT-scanned, sideritic bromalite.
**Muz PGI OS-221/341**	Elongated, M4	Theropod (large-sized).	259×115×91	Bone on the bromalite surface.	Surface observations. CT-scanned, sideritic bromalite.
**Muz PGI OS-221/391**	Elongated, M4	Theropod (large-sized).	221×85×35	Bone found on broken surface.	Surface observations. CT-scanned, sideritic bromalite.
**Muz PGI OS-221/303**	Elongated, M5	Theropod (medium-sized).	220×82×35	Fish remains (scales, teeth), bone fragments.	Surface observations. Phosphatic bromalite. Studied by methods SEM, EDS.
**Muz PGI OS-221/304**	Elongated, M5	Theropod (medium-sized).	216×81×35	Fish remains, bone fragments	Phosphatic bromalite. Studied by methods SEM, EDS.
**Muz PGI OS-221/345**	Elongated, M4	Theropod (medium-sized).	124×72×53	Fish remains.	Surface observations. Phosphatic bromalite. Studied by methods SEM, EDS.
**Muz PGI OS-221/346**	Elongated, M4	Theropod (medium-sized).	134×68×59	Fish remains, bone fragments.	Surface observations. Phosphatic bromalite. Studied by methods SEM, EDS.
**Muz PGI OS-221/402**	Elongated, M4	Theropod (medium-sized).	110×49×35	Fish remains, bone fragments.	Surface observations. Phosphatic bromalite.
**Muz PGI OS-221/439**	Elongated, M4	Theropod (medium-sized).	125×47×35	Bone fragments, plant remains.	Surface observations. Phosphatic bromalite.
**Muz PGI OS-221/305**	Elongated, fusiform, M6	Ornithischian	-	Plant remains.	Surface observations. Sideritic bromalite.
**Muz PGI OS-221/307**	Elongated, M6	Ornithischian	-	Plant remains.	Surface observations. Sideritic bromalite
**Muz PGI OS-221/321**	Elongated, fusiform M6	Ornithischian	-	Plant remains.	Surface observations. Sideritic bromalite
**Muz PGI OS-221/327**	Elongated, M6	Ornithischian	-	Plant remains.	Surface observations. Sideritic bromalite
**Muz PGI OS-221/328**	Elongated, M6	Ornithischian	-	Plant remains.	Surface observations. Sideritic bromalite
**Muz PGI OS-221/332**	Elongated, M6	Ornithischian	-	Plant remains.	Surface observations. Sideritic bromalite
**Muz PGI OS-221/335**	Elongated, M6	Ornithischian	-	Plant remains.	Surface observations. Sideritic bromalite
**Muz PGI OS-221/336**	Elongated, M6	Ornithischian	-	Plant remains.	Surface observations. Sideritic bromalite
**Muz PGI OS-221/337**	Elongated, M6	Ornithischian	-	Plant remains.	Surface observations. Sideritic bromalite

SUPPLEMENTARY TABLES

**Muz PGI OS-221/338**	Elongated, large, M7	Sauropodomorph	-	Plant remains.	Surface observations. Sideritic bromalite
**Muz PGI OS-221/342**	Elongated, large, M7	Sauropodomorph	-	Plant remains.	Surface observations. Sideritic bromalite
**Muz PGI OS-221/343**	Elongated, M7	Sauropodomorph	-	Plant remains.	Surface observations. Sideritic bromalite
**Muz PGI OS-221/344**	Elongated, irregular M8	Sauropodomorph	-	Plant remains.	Surface observations. Sideritic bromalite
**Muz PGI OS-221/345**	Elongated, irregular M8	Sauropodomorph	-	Plant remains.	Surface observations. Sideritic bromalite
**Muz PGI OS-221/348**	Elongated, irregular M8	Sauropodomorph	-	Plant remains.	Surface observations. Sideritic bromalite
**Muz PGI OS-221/349**	Elongated, irregular, M8	Sauropodomorph	-	Plant remains.	Surface observations. Sideritic bromalite
**Muz PGI OS-221/350**	Elongated, large, M7	Sauropodomorph	-	Plant remains.	Surface observations. Sideritic bromalite
**Muz PGI OS-221/351**	Elongated, large, M7	Sauropodomorph	-	Plant remains.	Surface observations. Sideritic bromalite
**Muz PGI OS-221/352**	Elongated, M6	Ornithischian	99×41×39	Plant remains.	Surface observations. Sideritic bromalite
**Muz PGI OS-221/353**	Elongated, M6	Ornithischian	113×42×31	Plant remains.	Surface observations. Sideritic bromalite
**Muz PGI OS-221/354**	Elongated, M6	Ornithischian	106×40×32	Plant remains.	Surface observations. Sideritic bromalite
**Muz PGI OS-221/355**	Elongated, M6	Ornithischian	109×48×46	Plant remains.	Surface observations. Sideritic bromalite
**Muz PGI OS-221/356**	Elongated, M6	Ornithischian	134×45×37	Plant remains.	Surface observations. Sideritic bromalite
**Muz PGI OS-221/357**	Elongated, M6	Ornithischian	128×47×39	Plant remains.	Surface observations. Sideritic bromalite
**Muz PGI OS-221/358**	Elongated, M6	Ornithischian	139×49×31	Plant remains.	Surface observations. Sideritic bromalite.
**Muz PGI OS-221/359**	Elongated, M6	Ornithischian	98×43×32	Plant remains.	Surface observations. Sideritic bromalite.
**Muz PGI OS-221/360**	Elongated, M6	Ornithischian	145×46×34	Plant remains.	Surface observations. Sideritic bromalite.
**Muz PGI OS-221/361**	Elongated, M6	Ornithischian	123×34×25	Plant remains.	Surface observations. Sideritic bromalite.
**Muz PGI OS-221/362**	Elongated, M6	Ornithischian	67×33×23	Plant remains.	Surface observations. Sideritic bromalite.
**Muz PGI OS-221/363**	Elongated, M6	Ornithischian	49×38×31	Plant remains.	Surface observations. Sideritic bromalite.
**Muz PGI OS-221/364**	Oval, irregular, M9	Sauropodomorph	56×39×21	Plant remains.	Surface observations. Sideritic bromalite.
**Muz PGI OS-221/365**	Elongated, M6	Ornithischian	67×34×29	Plant remains.	Surface observations. Sideritic bromalite.
**Muz PGI OS-221/366**	Elongated, M6	Ornithischian	73×32×20	Plant remains.	Surface observations. Sideritic bromalite.
**Muz PGI OS-221/367**	Elongated, M6	Ornithischian	73×37×28	Plant remains.	Surface observations. Sideritic bromalite.
**Muz PGI OS-221/368**	Elongated, M6	Ornithischian	59×34×31	Plant remains.	Surface observations. Sideritic bromalite.
**Muz PGI OS-221/369**	Elongated, M6	Ornithischian	65×32×35	Plant remains.	Surface observations. Sideritic bromalite.
**Muz PGI OS-221/370**	Elongated, M6	Ornithischian	75×36×27	Plant remains.	Surface observations. Sideritic bromalite.
**Muz PGI OS-221/371**	Elongated, M6	Ornithischian	79×32×31	Plant remains.	Surface observations. Sideritic bromalite.
**Muz PGI OS-221/372**	Elongated, M6	Ornithischian	121×34×22	Not studied.	Surface observations. Sideritic bromalite.
**Muz PGI OS-221/373**	Elongated, M6	Ornithischian	57×24×10	Not studied.	Surface observations. Sideritic bromalite.
**Muz PGI OS-221/376 SOL1**	Elongated, M6	Ornithischian	80×27×12	Plant remains, organic clasts, dark clasts.	Surface observations. Sideritic bromalite, specimen dissolved.
**Muz PGI OS-221/377 SOL2**	Elongated, M6	Ornithischian	67×23×21	Plant remains, dark clasts	Surface observations. Sideritic bromalite, specimen dissolved.
**Muz PGI OS-221/378 SOL3**	Elongated, M6	Ornithischian	95×27×25	Plant remains.	Surface observations. Sideritic bromalite, specimen dissolved.
**Muz PGI OS-221/379 SOL4**	Elongated, M6	Ornithischian	65×29×12	Plant remains.	Surface observations. Sideritic bromalite, specimen dissolved.
**Muz PGI OS-221/380 SOL5**	Elongated, large, M7	Sauropodomorph	93×26×19	Plant remains.	Surface observations. Sideritic bromalite, specimen dissolved.
**Muz PGI OS-221/381 SOL6**	Elongated, M6	Ornithischian	97×23×16	Plant remains.	Surface observations. Sideritic bromalite¸ specimen dissolved.
**Muz PGI OS-221/382 SOL7**	Elongated, massive, M8	Sauropodomorph	78×21×21	Plant remains.	Surface observations. Sideritic bromalite, specimen dissolved.

**Muz PGI OS-221/383 SOL8**	Elongated, massive, M8	Sauropodomorph	96×29×22	Plant remains.	Surface observations. Sideritic bromalite, specimen dissolved.
**Muz PGI OS-221/384 SOL9**	Elongated, M6	Ornithischian	108×26×26	Plant remains.	Surface observations. Sideritic bromalite, specimen dissolved.
**Muz PGI OS-221/385 SOL10**	Elongated, M6	Ornithischian	95×22×21	Plant remains.	Surface observations. Sideritic bromalite, specimen dissolved.
**Muz PGI OS-221/386**	Elongated, M6	Ornithischian	89×21×20	Plant remains.	Surface observations. Sideritic bromalite.
**Muz PGI OS-221/387**	Elongated, M6	Ornithischian	113×27×21	Plant remains.	Surface observations. Sideritic bromalite.
**Muz PGI OS-221/388**	Elongated, M6	Ornithischian	106×29×23	Plant remains.	Surface observations. Sideritic bromalite.
**Muz PGI OS-221/389**	Elongated, M6	Ornithischian	102×24×21	Plant remains.	Surface observations. Sideritic bromalite.
**Muz PGI OS-221/390**	Elongated, M6	Ornithischian	89×28×18	Plant remains.	Surface observations. Sideritic bromalite.
**Muz PGI OS-221/391**	Elongated, M6	Ornithischian	45×27×12	Plant remains.	Surface observations. Sideritic bromalite.
**Muz PGI OS-221/392**	Elongated, M6	Ornithischian	49×29×16	Not studied.	Surface observations. Sideritic bromalite.
**Muz PGI OS-221/393**	Oval, M9	Sauropodomorph	-	Plant remains.	Surface observations. Sideritic bromalite.
**Muz PGI OS-221/394**	Oval, M9	Sauropodomorph	-	Plant remains.	Surface observations. Sideritic bromalite.
**Muz PGI OS-221/395**	Elongated, M4	Theropod (medium-sized).	-	Bone remains.	Surface observations. Sideritic bromalite.
**Muz PGI OS-221/396**	Elongated, M4	Theropod (medium-sized).	-	Bone remains.	Surface observations. Sideritic bromalite.
**Muz PGI OS-221/397**	Elongated, M4	Theropod (medium-sized).	-	Fish and plant remains.	Surface observations. Sideritic bromalite.
**Muz PGI OS-221/398**	Elongated, M4	Theropod (medium-sized).	-	Poorly preserved inclusions.	Surface observations. Sideritic bromalite.
**Muz PGI OS-221/399**	Elongated, M4	Theropod (medium-sized).	-	Fish remains.	Surface observations. Sideritic bromalite.
**Muz PGI OS-221/400**	Elongated, M4	Theropod (medium-sized).	-	Fish remains.	Surface observations. Sideritic bromalite.
**Muz PGI OS-221/401**	Elongated, M4	Theropod (medium-sized).	-	Not studied	Surface observations. Sideritic bromalite.
**Muz PGI OS-221/403**	Elongated, M4	Theropod (medium-sized).	-	Bone remains.	Surface observations. Sideritic bromalite.
**Muz PGI OS-221/404**	Elongated, M4	Theropod (medium-sized).	-	Bone remains.	Surface observations. Sideritic bromalite.
**Muz PGI OS-221/405**	Elongated, M4	Theropod (medium-sized).	-	Bone remains.	Surface observations. Sideritic bromalite.
**Muz PGI OS-221/406**	Oval, M9	Sauropodomorph	-	Plant remains.	Surface observations. Sideritic bromalite.
**Muz PGI OS-221/407**	Oval, M9	Sauropodomorph	-	Plant remains.	Surface observations. Sideritic bromalite.
**Muz PGI OS-221/408**	Oval, M9	Sauropodomorph	-	Plant remains.	Surface observations. Sideritic bromalite.
**Muz PGI OS-221/409**	Elongated, large, M7	Sauropodomorph	-	Not studied.	Surface observations. Sideritic bromalite.
**Muz PGI OS-221/410**	Elongated, large, M7	Sauropodomorph	-	Plant remains.	Surface observations. Sideritic bromalite.
**Muz PGI OS-221/411**	Elongated, M6	Ornithischian	-	Plant remains.	Surface observations. Sideritic bromalite.
**Muz PGI OS-221/412**	Elongated, M6	Ornithischian	-	Plant remains.	Surface observations. Sideritic bromalite.
**Muz PGI OS-221/413**	Elongated, large, M7	Sauropodomorph	-	Plant remains.	Surface observations. Sideritic bromalite.
**Muz PGI OS-221/414**	Elongated, M6	Ornithischian	-	Plant remains.	Surface observations. Sideritic bromalite.
**Muz PGI OS-221/415**	Oval, M9	Sauropodomorph	-	Plant remains.	Surface observations. Sideritic bromalite.
**Muz PGI OS-221/416**	Oval, M9	Sauropodomorph	-	Plant remains.	Surface observations. Sideritic bromalite.
**Muz PGI OS-221/417**	Elongated, large, M7	Sauropodomorph	-	Not studied.	Surface observations. Sideritic bromalite.
**Muz PGI OS-221/418**	Elongated, large, M7	Sauropodomorph	-	Plant remains.	Surface observations. Sideritic bromalite.
**Muz PGI OS-221/419**	Elongated, M6	Ornithischian	-	Plant remains.	Surface observations. Sideritic bromalite.
**Muz PGI OS-221/420**	Elongated, large, M7	Sauropodomorph	-	Plant remains.	Surface observations. Sideritic bromalite.
**Muz PGI OS-221/421**	Elongated, M6	Ornithischian	-	Plant remains.	Surface observations. Sideritic bromalite.
**Muz PGI OS-221/422**	Elongated, M6	Ornithischian	-	Plant remains.	Surface observations. Sideritic bromalite.

**Muz PGI OS-221/423**	Elongated, M6	Ornithischian	-	Plant remains.	Surface observations. Sideritic bromalite.
**Muz PGI OS-221/424**	Oval, M9	Sauropodomorph	-	Plant remains.	Surface observations. Sideritic bromalite.
**Muz PGI OS-221/425**	Oval, M9	Sauropodomorph	-	Plant remains.	Surface observations. Sideritic bromalite.
**Muz PGI OS-221/426**	Elongated, large, M7	Sauropodomorph	-	Plant remains.	Surface observations. Sideritic bromalite.
**Muz PGI OS-221/427**	Elongated, M 6	Ornithischian	-	Plant remains.	Surface observations. Sideritic bromalite.
**Muz PGI OS-221/428**	Oval, M9	Sauropodomorph	-	Plant remains.	Surface observations. Sideritic bromalite.
**Muz PGI OS-221/429**	Elongated, large, M7	Sauropodomorph	-	Plant remains.	Surface observations. Sideritic bromalite.
**Muz PGI OS-221/430**	Scroll, M10	Large actinopterygian/ hybodont shark	-	Plant remains.	Surface observations. Sideritic bromalite.
**Muz PGI OS-221/431**	Scroll, M10	Large actinopterygian/ hybodont shark	-	Plant remains.	Surface observations. Sideritic bromalite.
**Muz PGI OS-221/432**	Scroll, M10	Large actinopterygian/ hybodont shark	-	Plant remains.	Surface observations. Sideritic bromalite.
**Muz PGI OS-221/433**	Scroll, M10	Large actinopterygian/ hybodont shark	-	Plant remains.	Surface observations. Sideritic bromalite.
**Muz PGI OS-221/434**	Scroll, M11	Small actinopterygian/ hybodont shark	-	Plant remains.	Surface observations. Sideritic bromalite.
**Muz PGI OS-221/435**	Elongated, fusiform, M6	Ornithischian	-	Not studied.	Surface observations. Sideritic bromalite.
**Muz PGI OS-221/436**	Elongated, M6	Ornithischian	-	Not studied.	Surface observations. Sideritic bromalite.
**Muz PGI OS-221/437**	Elongated, M6	Ornithischian	-	Plant remains.	Surface observations. Sideritic bromalite.
**Muz PGI OS-221/438**	Oval, M9	Sauropodomorph	-	Plant remains.	Surface observations. Sideritic bromalite.
**Muz PGI OS-221/440**	Elongated, massive, M8	Sauropodomorph	-	Plant remains.	Surface observations. Sideritic bromalite.
**Muz PGI OS-221/441**	Elongated, M6	Ornithischian	-	Plant remains.	Surface observations. Sideritic bromalite.
**Muz PGI OS-221/442**	Elongated, M6	Ornithischian	-	Plant remains.	Surface observations. Sideritic bromalite.
**Muz PGI OS-221/443**	Elongated, M6	Ornithischian	-	Plant remains.	Surface observations. Sideritic bromalite.
**Muz PGI OS-221/444**	Elongated, M6	Ornithischian	-	Plant remains.	Surface observations. Sideritic bromalite.
**Muz PGI OS-221/445**	Elongated, M6	Ornithischian	-	Plant remains.	Surface observations. Sideritic bromalite.
**Muz PGI OS-221/446**	Elongated, massive M8	Sauropodomorph	-	Plant remains.	Surface observations. Sideritic bromalite.
**Muz PGI OS-221/447**	Elongated, massive M8	Sauropodomorph	-	Plant remains.	Surface observations. Sideritic bromalite.
**Muz PGI OS-221/448**	Elongated, M6	Ornithischian	-	Not studied.	Surface observations. Sideritic bromalite.
**Muz PGI OS-221/449**	Elongated, large, M7	Sauropodomorph	-	Plant remains.	Surface observations. Sideritic bromalite.
**Muz PGI OS-221/450**	Elongated, large, M7	Sauropodomorph	-	Plant remains.	Surface observations. Sideritic bromalite.
**Muz PGI OS-221/451**	Elongated, large, M7	Sauropodomorph	-	Plant remains.	Surface observations. Sideritic bromalite.
**Muz PGI OS-221/452**	Oval, M9	Sauropodomorph	-	Plant remains.	Surface observations. Sideritic bromalite.
**Muz PGI OS-221/453**	Oval, M9	Sauropodomorph	-	Plant remains.	Surface observations. Sideritic bromalite.
**Muz PGI OS-221/454**	Oval, M9	Sauropodomorph	-	Plant remains.	Surface observations. Sideritic bromalite.
**Muz PGI OS-221/455**	Oval, M9	Sauropodomorph	-	Plant remains.	Surface observations. Sideritic bromalite.
**Muz PGI OS-221/456**	Oval, M9	Sauropodomorph	-	Plant remains.	Surface observations. Sideritic bromalite.
**Muz PGI OS-221/457**	Elongated, M6	Ornithischian	-	Plant remains.	Surface observations. Sideritic bromalite.
**Muz PGI OS-221/458**	Elongated, large, M7	Sauropodomorph	-	Plant remains.	Surface observations. Sideritic bromalite.
**Muz PGI OS-221/459**	Oval, M9	Sauropodomorph	-	Plant remains.	Surface observations. Sideritic bromalite.
**Muz PGI OS-221/460**	Elongated, M6	Ornithischian	-	Plant remains.	Surface observations. Sideritic bromalite.

SUPPLEMENTARY TABLES

**Muz PGI OS-221/461**	Oval, M9	Sauropodomorph	-	Plant remains.	Surface observations. Sideritic bromalite.
**Muz PGI OS-221/462**	Elongated, fusiform, M6	Ornithischian	-	Plant remains.	Surface observations. Sideritic bromalite.
**Muz PGI OS-221/463**	Elongated, fusiform, M6	Ornithischian	-	Not studied	Surface observations. Sideritic bromalite.
**Muz PGI OS-221/464**	Elongated, M6	Ornithischian	-	Not studied	Surface observations. Sideritic bromalite.
**Muz PGI OS-221/465**	Elongated, M6	Ornithischian	-	Not studied	Surface observations. Sideritic bromalite.

Supplementary Table 10: Bulk geochemical data of bromalites from Sołtyków. Specimens Muz PGI OS-221/376-384 (SOL_1-9), for more details see Supp. Tab. 9.

**Sample**	**TIC [%]**	**TC[%]**	**TS[%]**	**TOC[%]**
SOL_1a	2.0	5.7	0.1	**3.7**
SOL_1b	1.3	5.9	0.1	**4.6**
SOL_1c	1.1	5.9	0.1	**4.8**
SOL_2	0.0	0.5	0.0	**0.5**
SOL_3	0.0	10.5	0.1	**10.5**
SOL_4	0.0	41.7	0.3	**41.7**
SOL_5	0.0	6.5	0.1	**6.5**
SOL_6	0.0	0.7	0.0	**0.7**
SOL_7	0.0	4.9	0.1	**4.9**
SOL_8a	0.9	2.1	4.7	**1.2**
SOL_8b	1.0	2.0	0.5	**1.0**
SOL_9a	10.6	10.7	0.0	**0.1**
SOL_9b	10.8	11.0	0.0	**0.1**

TIC = total inorganic carbon; TC = total carbon; TS = total sulphur; TOC = total organic carbon

Supplementary Table 11: Organic parameters based on the distribution of aliphatic hydrocarbons for the bromalite samples from Sołtyków. Specimens Muz PGI OS-221/376-384 (SOL_1-9), for more details see Supp. Tab. 9.

Sample	CPI	$CPI_{(25-31)}$	Pr/Ph	Pr/*n*-C_{17}	Ph/*n*-C_{18}	SCh/LCh
SOL_1a	1.18	1.83	1.12	1.19	0.42	0.75
SOL_1b	1.02	1.72	0.99	0.86	0.32	1.39
SOL_1c	1.03	1.61	0.94	0.88	0.35	1.14
SOL_2	1.01	1.21	1.15	0.62	0.31	0.45
SOL_3	2.03	2.20	4.73	4.68	0.56	0.19
SOL_4	1.03	1.25	0.94	0.52	0.32	0.98
SOL_5	1.10	1.33	3.97	2.90	0.48	0.78
SOL_6	0.94	1.09	1.45	0.46	0.28	1.18
SOL_7	1.17	1.37	2.89	2.70	0.46	0.61
SOL_8a	1.07	1.15	2.59	0.88	0.58	2.19
SOL_8b	1.04	1.10	2.48	0.80	0.48	2.59
SOL_9a	tr	tr	tr	tr	tr	tr
SOL_9b	tr	tr	tr	tr	tr	tr

CPI = carbon preference index
$CPI_{(25–31)}$ = carbon preference index: $(C_{25} + C_{27} + C_{29}) + (C_{27} + C_{29} + C_{31})/2(C_{26} + C_{28} + C_{30})$;
SCh/LCh = $(nC_{17}+nC_{18}+nC_{19})/(nC_{27}+nC_{28}+nC_{29})$; Pr = Pristane; Ph = Phytane; tr = traces

Supplementary Table 12: Concentrations (μm/g TOC) of most abundant PAHs identified in bromalite samples (in elution order). Specimens Muz PGI OS-221/376-384 (SOL_1-9), for more details see Supp. Tab. 9.

SAMPLES	Phen	Fl	Py	B*a*A	Chr & Triph	B*b*Fl	B*e*P	B*a*P	Per	B[*ghi*]Pe	Cor	PAHs1	PAHs2
SOL_1a	1.30	2.68	10.27	2.48	4.79	18.25	16.45	15.15	13.03	35.86	2.00	54.34	84.21
SOL_1b	1.52	2.05	5.32	1.54	3.42	9.63	7.78	5.92	4.97	6.77	6.52	31.39	36.21
SOL_1c	1.86	3.22	7.58	2.37	5.17	19.30	16.70	14.61	13.07	36.00	2.46	55.43	83.16
SOL_2	0.22	0.35	0.55	0.60	0.70	1.25	1.15	1.00	0.90	2.00	0.20	4.20	5.35
SOL_3	0.54	0.48	0.74	0.51	1.16	2.14	1.21	1.15	0.54	1.90	0.43	5.44	6.84
SOL_4	5.21	0.30	0.33	0.68	0.79	1.45	1.26	1.03	0.97	0.97	0.25	4.67	4.33
SOL_5	7.31	2.63	4.87	10.54	10.24	16.28	15.93	8.83	0.30	3.50	1.60	53.17	37.71
SOL_6	0.15	0.12	0.45	0.60	0.74	0.86	0.81	0.77	0.69	0.95	0.25	3.29	3.40
SOL_7	4.45	1.79	3.00	11.07	10.56	19.00	17.63	9.10	0.21	2.66	1.44	58.24	36.99
SOL_8a	0.17	0.14	0.49	0.69	0.84	0.93	0.88	0.72	0.20	0.54	0.05	3.37	3.27
SOL_8b	0.10	0.21	0.54	0.78	0.94	0.83	0.80	0.74	0.22	0.84	0.15	3.27	2.87
SOL_9a	nd	nd	nd	nd	nd	nd	nd	nd	nd	nd	nd	nd	nd
SOL_9b	nd	nd	nd	nd	nd	nd	nd	nd	nd	nd	nd	nd	nd

Phen = phenanthrene; Fl = fluoranthene; Py = pyrene; B*a*A = benz[*a*]anthracene; Chr = chrysene; Triph = triphenylene; B*b*Fl = benzo[*b*]fluoranthene;
B*e*P = benzo[*e*]pyrene; B*a*P = benzo[*a*]pyrene; Per = perylene; B[*ghi*]Pe = benzo[*ghi*]perylene; Cor = coronene
PAHs1=B*a*A+B*b*Fl+B*e*P+B*a*P+Cor (Marynowski and Simoneit, 2009), PAHs2=Fl+Py+B*b*Fl+B*a*P+B[*ghi*]P+Cor (Finklestein et al., 2005).
nd = not detected

Supplementary Table 13: Sedimentological and taphonomic characteristics of fossil assemblages with bromalites.

Locality	Age/Assemblage/Lithostratigraphy	Sedimentology/ Palaeoenvironmental interpretation	Bromalite occurrences	Total number of studied bromalites and the ratio of specimens poor to those rich in plant matter	Recognized processes of fossilization and diagenesis
**Krasiejów**	Middle-Late Carnian (Late Triassic).<br><br>Krasiejów-Woźniki assemblage.<br><br>Lithostratigraphy: Grabowa Formation or Drawno Beds (informal unite).	The fossil-bearing succession is composed of gravelly sandstones siltstones and mudstones/claystones.<br><br>These deposits represent a low-energy anastomosing river system in a tropical semiarid or arid climate.	Bromalites co-occur with two, major fossil assemblages (so-called the Upper and Lower bone-beds). Finds of bromalite are rare in both levels.	127 bromalites (including one regurgitatlite with numerous bone fragments). **100%** of the collected bromalites represent phosphate- or carbonate-rich specimens. There are no bromalites with a larger mass of plant remains, but isolated plant cuticles and palynomorphs have been identified in carbonate-rich specimens.	Phosphatization, early diagenesis with P, Ca, Fe participation supported by microbial mineralization processes. Lithification before sediment compaction. **Preserved inclusions:** bones, fish scales, teeth and phosphatized soft tissue, arthropod remains, palynomorphs and plant cuticles.
**Woźniki**	Middle-Late Carnian (Late Triassic).<br><br>Krasiejów-Woźniki assemblage.<br><br>Lithostratigraphy: Grabowa Formation or Drawno Beds (informal unite).	The fossil-bearing succession is composed of gravelly sandstones, siltstones and mudstones/claystones.<br><br>Succession represents a low-energy anastomosing river system in a tropical semiarid or arid climate.	Two intervals with bromalites have been identified in the section. Bromalites co-occur with tetrapod bones. In both intervals, bromalites are rare.	15 bromalites.<br>**100%** of the collected bromalites represent phosphate or carbonate fossils. There are no bromalites with a larger mass of plant remains, isolated plant cuticles and palynomorphs were identified in phosphate and carbonate specimen.	Phosphatization, early diagenesis with the P, Ca, Fe participation supported by microbial mineralization processes. Lithification before sediment compaction. **Preserved inclusions:** bones, scales, teeth and phosphatized soft tissue, palynomorphs and plant cuticles.
**Poręba**	Middle-Late Norian (Late Triassic).<br><br>Poręba-Kocury assemblage.<br><br>Lithostratigraphy: Grabowa Formation or Zbaszynek Beds (informal unite).	The fossil-bearing succession is composed of conglomerates, sandstones, siltstones, mudstones/claystones.<br><br>Succession represents anastomosing river system in a tropical semiarid climate.	The interval with bromalites overlaps with the upper interval with bones. The bromalites are abundant.	46 bromalites, have been found in the conglomerate and mudstone intervals and are associated with micro- and macroremains of plant and bone fragments.<br>About **50%** of the studied bromalites show content of plant remains or local accumulations of plant cuticles.	Phosphatization, early diagenesis with the P, Ca, Fe participation supported by microbial mineralization processes. Lithification before sediment compaction. **Preserved inclusions:** bones, fish scales, teeth and phosphatized soft tissue, palynomorphs, plant fragments and plant cuticles.
**Kocury**	Middle-Late Norian (Late Triassic).<br><br>Poręba-Kocury assemblage.<br><br>Lithostratigraphy: Grabowa Formation or Zbaszynek Beds (informal unite).	The fossil-bearing succession is composed of conglomerates, sandstones, siltstones, mudstones/claystones.<br><br>Succession represents anastomosing river system in a tropical semiarid climate.	No bromalite finds.	---	---
**Lisowice**	Late Norian-earliest Rhaetian (Late Triassic).<br><br>Lisowice-Marciszów assemblage.	The fossil-bearing succession is composed of conglomerates, sandstones, siltstones, mudstones/claystones.	The interval with bromalites overlaps with the upper interval with bones. The	Nearly 300 bromalites have been found in the conglomeratic beds and mudstones/claystones intervals rich in plant remains. About **70%** of the studied	Phosphatization, early diagenesis with the P, Ca, Fe participation supported by microbial

SUPPLEMENTARY TABLES

	Lithostratigraphy: Grabowa Formation or Zbaszynek Beds/Wielichowo Beds (informal unites).	Succession represents anastomosing river system in a tropical semiarid to humid climate.	bromalites are abundant.	bromalites show content of plant remains or local accumulations of plant cuticles.	mineralization processes.Lithification before sediment compaction. **Preserved inclusions:** bones, scales, teeth and phosphatized soft tissue, palynomorphs, plant fragments and plant cuticles.
**Marciszów**	Late Norian-earliest Rhaetian (Late Triassic).<br><br>Lisowice-Marciszów assemblage.<br><br>Lithostratigraphy: Grabowa Formation or Zbaszynek Beds/Wielichowo Beds (informal unites).	The fossil-bearing succession is composed of conglomerates, sandstones, siltstones, mudstones/claystones.<br><br>Succession represents anastomosing river system in a tropical semiarid to humid climate.	Specimens collected from a mound near the excavation and their position in the section are not specified.	5 bromalites, all represent plant-rich specimens.	Lithification before sediment compaction. **Preserved inclusions:** palynomorphs, plant fragments and plant cuticles.
**Gromadzice-Rzuchów**	Middle-Late Rhaetian (latest Triassic).<br><br>Gromadzice-Rzuchów assemblage.<br><br>Lithostratigraphy: Parszów Beds (informal unite).	The fossil-bearing succession is composed of sandstones siltstones and mudstones/claystones.<br><br>These deposits represent anastomosing river system in a tropical humid climate.	Bromalites co-occur with plant and invertebrate fossils (ostracods and bivalves). Specimens are poorly preserved and are rare.	12 bromalites. About **60%** of the studied bromalites show content of plant remains or accumulations of plant cuticles.	Phosphatization, carbonatization, secondary sideritization. Lithification before sediment compaction. **Preserved inclusions:** bones, fish scales, plant remains and plant cuticles.
**Hucisko**	Latest Rhaetian-early Hettangian (latest Triassic-earliest Jurassic).<br><br>Sołtyków-Hucisko assemblage.<br><br>Lithostratigraphy: Zagaje Formation.	The bone and bromalite-bearing beds are mudstones, claystones and sandstones with numerous charcoal fragments.<br><br>These strata represents anastomosing river system in a tropical humid climate.	Bromalites co-occur with well-preserved vertebrate remains. All bromalites are collected from the bone-bearing bed, are preserved as phosphatic or secondary siderized specimens and are abundant.	16 bromalites. **100%** of the bromalites represent phosphate- or carbonate-rich specimens (in major part specimens are secondary sideritized).	Phosphatization, secondary sideritization. Lithification before sediment compaction. **Preserved inclusions:** bones, scales, teeth, and plant cuticles.
**Sołtyków**	Latest Rhaetian-early Hettangian (latest Triassic-earliest Jurassic).<br><br>Sołtyków-Hucisko assemblage.<br><br>Lithostratigraphy: Zagaje Formation.	The fossil-bearing succession is composed of sandstones, mudstones, siltstones and mudstones/claystones.<br><br>These strata were deposited in a meandering river system in a tropical humid climate.	Bromalites co-occur with various fossils (plant roots, plant fragments, vertebrate remains). Four intervals with bromalites have been recognized. In all intervals, bromalites are abundant.	About 300 bromalites. **60%** of the studied (148) bromalites show substantial content of plant remains or accumulations of plant cuticles.	Phosphatization, carbonatization, secondary sideritization. Lithification before sediment compaction. **Preserved inclusions:** bones, fish scales, fish teeth, palynomorphs, plant fragments and plant cuticles.
